# Neutral iridium catalysts with chiral phosphine-carboxy ligands for asymmetric hydrogenation of unsaturated carboxylic acids†
†Electronic supplementary information (ESI) available: For CIF data of (*S*)-**2d**, experimental procedures, and characterization data. CCDC 1498903. For ESI and crystallographic data in CIF or other electronic format see DOI: 10.1039/c6sc03764j



**DOI:** 10.1039/c6sc03764j

**Published:** 2016-11-15

**Authors:** Shuang Yang, Wen Che, Hui-Ling Wu, Shou-Fei Zhu, Qi-Lin Zhou

**Affiliations:** a State Key Laboratory and Institute of Elemento-Organic Chemistry , Nankai University , Tianjin 300071 , China . Email: sfzhu@nankai.edu.cn ; Email: qlzhou@nankai.edu.cn; b Collaborative Innovation Center of Chemical Science and Engineering (Tianjin) , Tianjin 300071 , China

## Abstract

We developed neutral iridium catalysts with chiral spiro phosphine-carboxy ligands (SpiroCAP) for asymmetric hydrogenation of unsaturated carboxylic acids. Different from the cationic Crabtree-type catalysts, the iridium catalysts with chiral spiro phosphine-carboxy ligands are neutral and do not require the use of a tetrakis[3,5-bis(trifluoromethyl)phenyl]borate (BAr_F_^–^) counterion, which is necessary for stabilizing cationic Crabtree-type catalysts. Another advantage of the neutral iridium catalysts is that they have high stability and have a long lifetime in air. The new iridium catalysts with chiral spiro phosphine-carboxy ligands exhibit unprecedented high enantioselectivity (up to 99.4% ee) in the asymmetric hydrogenations of various unsaturated carboxylic acids, particularly for 3-alkyl-3-methylenepropionic acids, which are challenging substrates for other chiral catalysts.

## Abstract 2

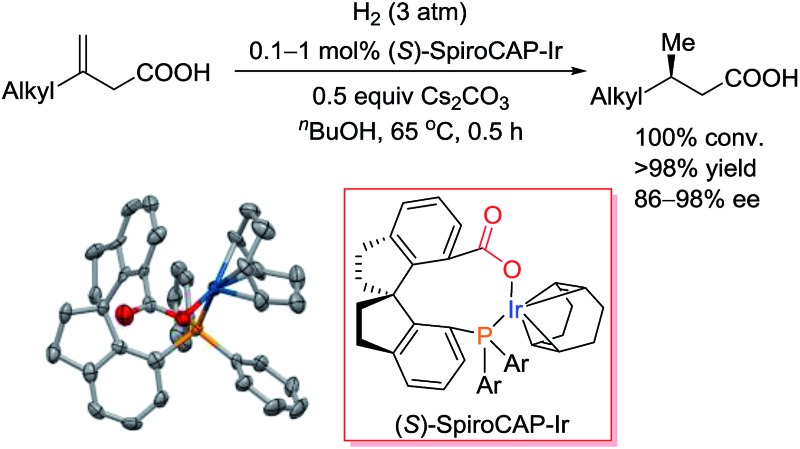
The new neutral iridium catalyst with chiral spiro phosphine-carboxy ligand exhibited exceptionally high enantioselectivity in the hydrogenation of 3-alkyl-3-methylenepropionic acids.

## Introduction

Transition-metal-catalyzed asymmetric hydrogenation is one of the most useful reactions for the synthesis of optical compounds because of its perfect atom economy and operational simplicity.^1^ Chiral ligands are essential for this reaction. In the past decades, a tremendous number of chiral ligands have been developed for the asymmetric hydrogenation of pro-chiral unsaturated compounds.^2^ However, there are few chiral ligands in which oxygen is the coordinating atom for asymmetric hydrogenation of olefins.^3^ A few chiral phosphine–amide ligands with oxygen as the coordinating atom have been reported for rhodium- and iridium-catalyzed asymmetric hydrogenation reactions of cyclic enamides and trisubstituted olefins, respectively, with moderate to high enantioselectivity.^3a,3b^

**Scheme 1:**
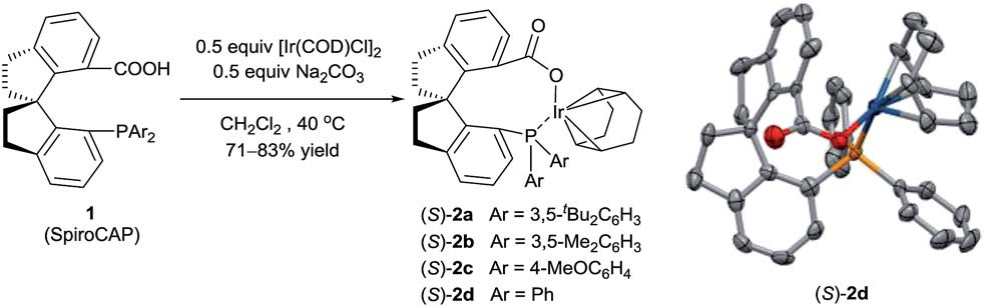
Synthesis of iridium complexes of chiral spiro phosphine-carboxy ligands. The crystal structure of (*S*)-**2d** (hydrogen atoms omitted for clarity). Selected bond lengths (Å) and angles (°) for (*S*)-**2d**: Ir–O 2.085(4), Ir–P 2.3516(17); O–Ir–P 90.30(15).

Recently, we developed iridium catalysts with chiral spiro phosphine–oxazoline ligands and chiral spiro aminophosphine ligands, which show high enantioselectivity for the asymmetric hydrogenation of unsaturated carboxylic acids.^4^ Mechanistic studies revealed that the carboxy group of the substrate anchors the molecule to the catalyst and triggers the hydrogenation reaction. On the basis of these results, we envisioned that iridium complexes with a chiral spiro phosphine-carboxy ligand might efficiently catalyze the asymmetric hydrogenation of unsaturated carboxylic acids. Carboxy groups can be introduced into chiral ligands to tune the electronic and steric properties of catalysts and thus offer new opportunities to achieve asymmetric hydrogenation of challenging substrates. Herein, we report the preparation of iridium complexes with chiral spiro phosphine-carboxy ligands (**2**, Scheme 1) and the use of the complexes for the hydrogenation of unsaturated carboxylic acids. These neutral iridium catalysts have several advantages: (1) they do not require the use of a tetrakis[3,5-bis(trifluoromethyl)phenyl]borate (BAr_F_^–^, has a molecular weight of 863) counterion, which is necessary for stabilizing chiral cationic Crabtree-type catalysts (also called Pfaltz catalysts);^5^ (2) they have high stability and have a long lifetime in air; and (3) they exhibit unprecedented high enantioselectivity (up to 99.4% ee) in the asymmetric hydrogenation of various unsaturated carboxylic acids, particularly for 3-alkyl-3-methylenepropionic acids, which are challenging substrates.

## Results and discussion

Iridium complexes **2** were readily prepared with high yields (71–83%) by refluxing a mixture of [Ir(COD)Cl]_2_, chiral spiro phosphine-carboxy ligands **1** (SpiroCAP),^6^ and Na_2_CO_3_ in CH_2_Cl_2_ for 1 h (Scheme 1). These neutral complexes were very stable; they can be purified by silica gel column chromatography and stored in air for a few months. The structure of (*S*)-**2d** was confirmed by X-ray diffraction analysis of a single crystal (Scheme 1), which revealed that (*S*)-**1d** coordinates to the iridium atom as an anionic ligand.^7^

Asymmetric hydrogenation of 3-alkyl-3-methylene-carboxylic acids **5** is a useful reaction because the products, chiral 3-alkyl-3-methylpropionic acids, are building blocks for biologically active compounds.^8^ However, there are no well-established methods for the asymmetric hydrogenation of 3-alkyl-3-methylene-carboxylic acids. Uchida *et al.* ^9^ and Zhang *et al.* ^10^ investigated this reaction by using Ru/BINAP and Rh/DuanPhos as catalysts, respectively, but achieved only moderate enantioselectivity (up to 74% ee). Alternatively, Pfaltz *et al.* ^11^ reported a hydrogenation of α,β-unsaturated esters giving 3-alkyl-3-methylpropionate esters in good enantioselectivities (up to 94% ee). We studied the asymmetric hydrogenation of 3-methyleneheptanoic acid (**5a**) with iridium complexes **2** as catalysts (Table 1). When the reaction was performed with (*S*)-**2a** in the presence of base Cs_2_CO_3_ under 3 atm of hydrogen in methanol at 45 °C, the desired product, 3-methylheptanoic acid (**6a**), was obtained in 82% ee (entry 1). Catalysts with less bulky *P*-aryl groups, such as 3,5-dimethylphenyl (**2b**), 4-methoxylphenyl (**2c**), and phenyl (**2d**), exhibited slightly higher enantioselectivities (entries 2–4). In contrast, catalysts (*S*)-**3** (ref. 4*c*) and (*S*)-**4** (ref. 4*f*) with chiral spiro P,N ligands gave much lower enantioselectivities (35% and 66% ee, respectively; entries 5 and 6). Other bases (K_2_CO_3_, Na_2_CO_3_, and NEt_3_) gave lower enantioselectivities (entries 7–9), and omission of the base resulted in a poor conversion of the substrate (entry 10). Performing the reaction at 65 °C slightly increased the enantioselectivity (entry 11). The use of higher-molecular-weight alcohols as solvents led to higher enantioselectivities (entries 12–15), and ^*n*^BuOH and ^*t*^BuOH proved to be the most suitable solvents, giving the desired product in 93% ee. When the carboxy group of the ligand **1a** was replaced by a methyl ester, only racemic product was obtained under identical reaction conditions, which clearly demonstrates that the coordination of carboxyl group of ligand to the metal of catalyst is necessary for enantiocontrol of reaction.

**Table 1:** Iridium-catalyzed asymmetric hydrogenation of 3-methyleneheptanoic acid^*a*^

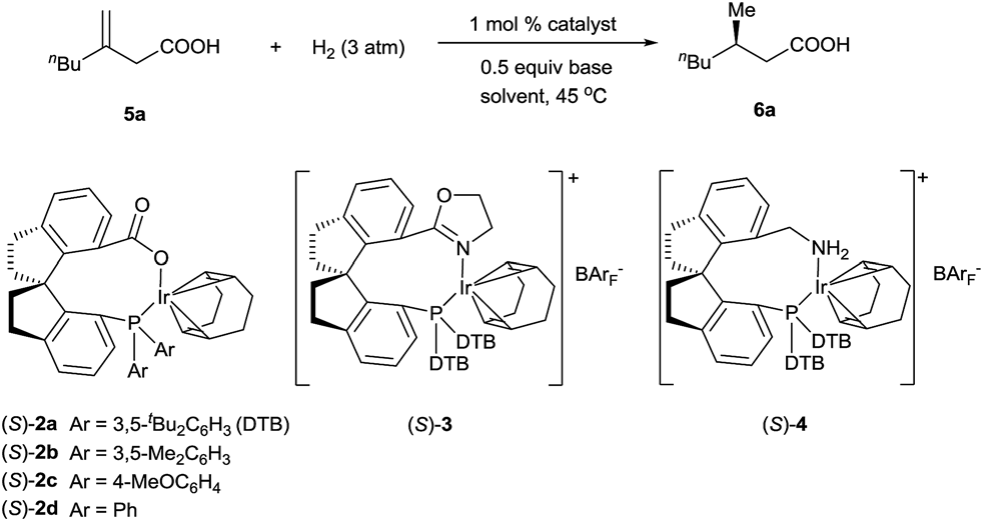						
Entry	Catalyst	Base	Solvent	Time (h)	Conv.^*b*^ (%)	ee^*c*^ (%)
1	(*S*)-**2a**	Cs_2_CO_3_	MeOH	0.5	100	82
2	(*S*)-**2b**	Cs_2_CO_3_	MeOH	0.5	100	83
3	(*S*)-**2c**	Cs_2_CO_3_	MeOH	0.5	100	83
4	(*S*)-**2d**	Cs_2_CO_3_	MeOH	0.5	100	84
5	(*S*)-**3**	Cs_2_CO_3_	MeOH	2	100	35
6	(*S*)-**4**	Cs_2_CO_3_	MeOH	0.5	100	66
7	(*S*)-**2d**	K_2_CO_3_	MeOH	0.5	100	78
8	(*S*)-**2d**	Na_2_CO_3_	MeOH	0.5	100	55
9^*d*^	(*S*)-**2d**	NEt_3_	MeOH	0.5	100	76
10	(*S*)-**2d**	None	MeOH	24	27	70
11^*e*^	(*S*)-**2d**	Cs_2_CO_3_	MeOH	0.5	100	88
12^*e*^	(*S*)-**2d**	Cs_2_CO_3_	^*n*^PrOH	0.5	100	90
13^*e*^	(*S*)-**2d**	Cs_2_CO_3_	^i^PrOH	0.5	100	91
14^*e*^	(*S*)-**2d**	Cs_2_CO_3_	^*n*^BuOH	0.5	100	93
15^*e*^	(*S*)-**2d**	Cs_2_CO_3_	^*t*^BuOH	0.5	100	93

^*a*^Reaction conditions: **5a**/catalyst/base = 0.5 : 0.005 : 0.25 (mmol), 2 mL solvent, 45 °C.

^*b*^Determined by ^1^H NMR.

^*c*^Determined by HPLC using a Chiralpak AD-H column.

^*d*^1.0 equiv of base was used.

^*e*^Performed at 65 °C.

Under the optimal reaction conditions (Table 1, entry 14), various 3-alkyl-3-methylenepropionic acids (**5a–5j**) were hydrogenated in the presence of 1 mol% catalyst (*S*)-**2d** (Table 2). All the tested substrates underwent hydrogenation within 0.5 h to afford the desired chiral 3-methyl-carboxylic acids with good to excellent enantioselectivity (86–98% ee). The steric bulk of the R group of the substrate had a remarkable influence on the enantioselectivity; bulky alkyl groups favored high enantioselectivity (**6d–6g**). The hydrogenation of **5j**, which has two C=C bonds, occurred only at the β-olefin; the remote olefin moiety was untouched.

**Table 2:** Asymmetric hydrogenation of 3-alkyl-3-methylenepropionic acids catalyzed by (*S*)-**2d** ^*a*^

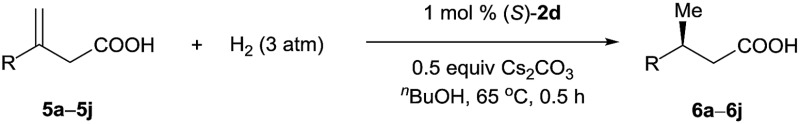
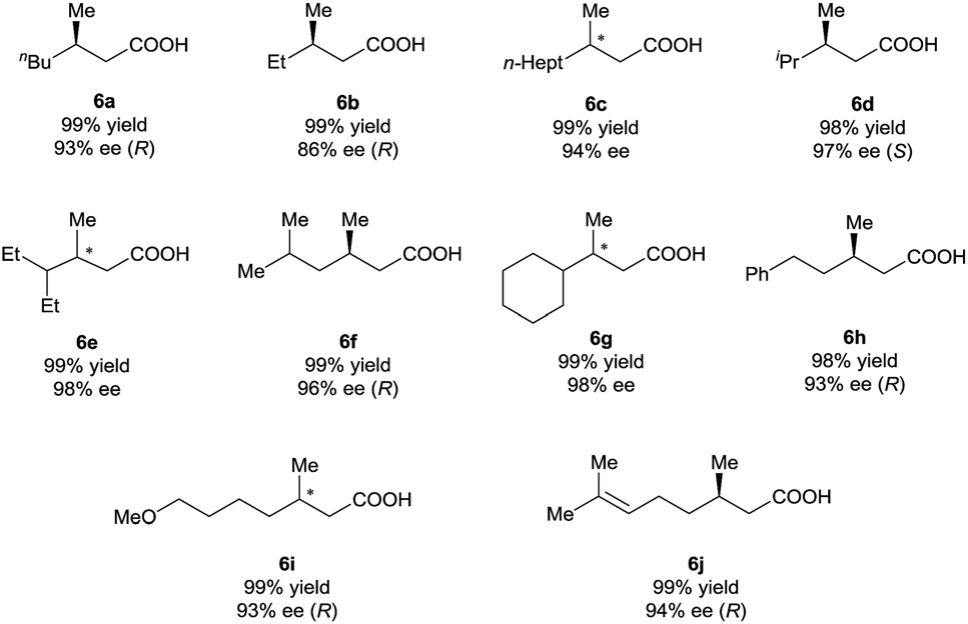

^*a*^Reaction conditions and analytical methods were the same as those described in entry 14 of Table 1. Full conversion was obtained in all cases.

**Scheme 2:**
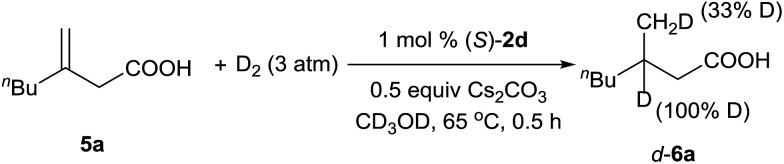
Deuterium-labeling experiment.

A deuterium-labeling experiment was performed to determine whether the olefin migrated during the hydrogenation reaction (Scheme 2). The absence of deuterium at the α-position of hydrogenation product *d*-**6a** clearly showed that the double bond did not migrate during the reaction.

In addition to 3-alkyl-3-methylenepropionic acids, other representative unsaturated carboxylic acids, such as α-methyl cinnamic acid (**7a**), tiglic acid (**7b**), α-phenyl butenoic acid (**7c**), α-benzyloxy cinnamic acid (**7d**), 2-(4-isobutylphenyl)acrylic acid (**7e**), and 2-methyleneheptanoic acid (**7f**), also underwent hydrogenation catalyzed by (*S*)-**2d** (Table 3), producing the corresponding chiral carboxylic acids in high yields (97–99%) with excellent enantioselectivities (94–99.4% ee). These results show that neutral iridium catalysts **2** are among the most efficient chiral catalysts reported to date for the asymmetric hydrogenation of unsaturated carboxylic acids.^4a,9–12^

**Table 3:** Hydrogenation of other types of unsaturated carboxylic acids^*a*^

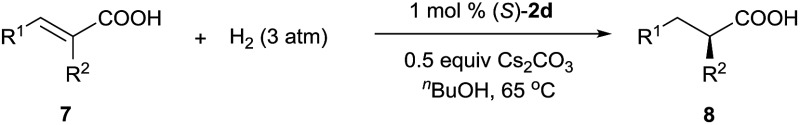
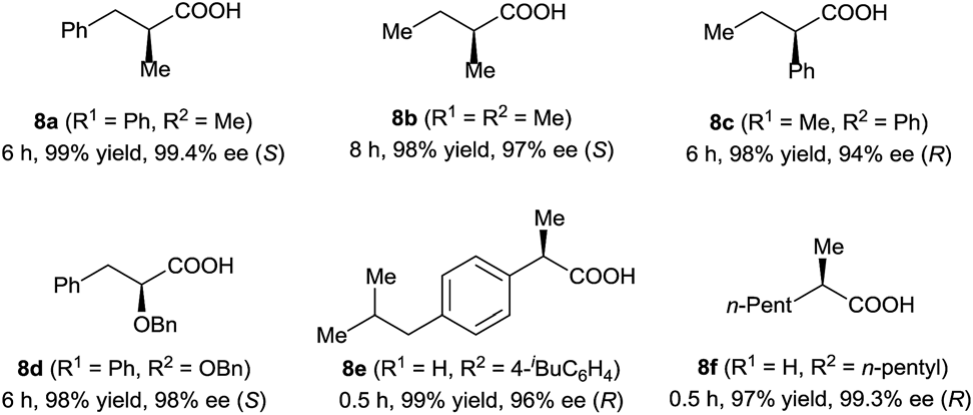

^*a*^Reaction conditions and analytical methods were the same as those described in entry 14 of Table 1. Full conversion was obtained in all cases.

**Scheme 3:**
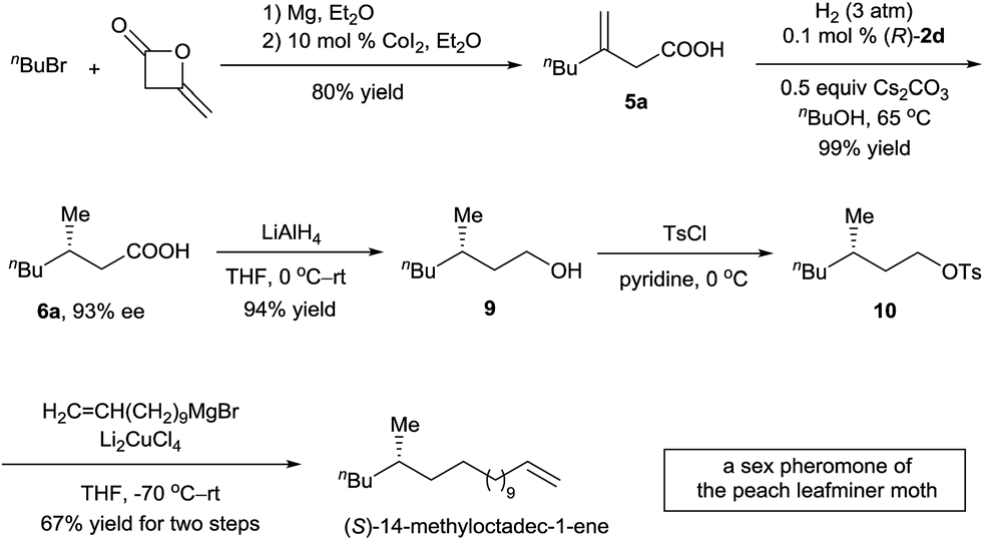
Total synthesis of (*S*)-14-methyloctadec-1-ene.

The (*S*)-14-methyloctadec-1-ene is a female sex pheromone of the peach leafminer moth (*Lyonetia clerkella*). Several syntheses of (*S*)-14-methyloctadec-1-ene have been reported using enzymatic catalysis,^13^ chiral starting materials,^14^ or chiral auxiliaries.^15^ To demonstrate the potential application of the asymmetric hydrogenation of 3-alkyl-3-methylenepropionic acids in organic synthesis, we carried out a synthesis of (*S*)-14-methyloctadec-1-ene (Scheme 3). 3-Methyleneheptanoic acid **5a**, which was easily prepared in 80% yield by a ring-opening reaction of commercially available 4-methyleneoxetan-2-one with a Grignard reagent, was hydrogenated in the presence of 0.1 mol% catalyst (*R*)-**2d** to produce acid (*S*)-**6a** in 99% yield with 93% ee. Acid (*S*)-**6a** was reduced with LiAlH_4_ to alcohol **9** in 94% yield. Finally, **9** was converted to (*S*)-14-methyloctadec-1-ene by tosylation and subsequent coupling with a Grignard reagent in 67% yield for the two steps. Thus, the total synthesis of (*S*)-14-methyloctadec-1-ene was accomplished in 50% overall yield starting from commercially available materials.

## Conclusions

In summary, we developed a new type of chiral spiro phosphine-carboxy ligand, which can form iridium complexes without the need for a stabilizing counterion. Neutral iridium complexes of these ligands catalyzed the asymmetric hydrogenation of various unsaturated carboxylic acids with excellent enantioselectivity. In particular, these catalysts allowed us to achieve highly enantioselective hydrogenation of 3-alkyl-3-methylenepropionic acids, which are challenging substrates for previously reported chiral catalysts. Additional studies on the applications of these neutral iridium catalysts with chiral phosphine-carboxy ligands to other asymmetric reactions are underway in our laboratory.
