# Supplementary material for: Neutral iridium catalysts with chiral phosphine-carboxy ligands for asymmetric hydrogenation of unsaturated carboxylic acids
Source: Chem Sci. 2016 Nov 15;8(3):1977–80. doi: 10.1039/c6sc03764j (PMC5384563; doi:10.1039/c6sc03764j)
Supplement: Supplementary file 1 [file SC-008-C6SC03764J-s001.pdf]

Electronic Supplementary Material (ESI) for Chemical Science.
This journal is © The Royal Society of Chemistry 2016

Supplementary Information for:

Neutral Iridium Catalysts with Chiral Phosphine-Carboxy Ligands for Asymmetric Hydrogenation of Unsaturated Carboxylic Acids

Shuang Yang,[a] Wen Che,[a] Hui-Ling Wu,[a] Shou-Fei Zhu*[a] and Qi-Lin Zhou*[ab]

[a] *State Key Laboratory and Institute of Elemento-Organic Chemistry, Nankai University, Tianjin 300071, China*

[b] *Collaborative Innovation Center of Chemical Science and Engineering (Tianjin), Tianjin 300071, China*

CONTENTS:

1. Preparation and Analytical Data of Iridium Complexes with Chiral Spiro Phosphine-Carboxy Ligands..........S3

2. Preparation and Analytical Data of Unsaturated Carboxylic Acids..........S4

3. Asymmetric Hydrogenation and Analytical Data of Products..........S6

4. Total Synthesis of (*S*)-14-Methyloctadec-1-ene..........S10

5. X-ray Diffraction Analysis of (*S*)-2d..........S11

6. NMR Spectra of New Compounds..........S13

7. HPLC or GC Charts of Hydrogenation Product Derivatives..........S29

8. Reference..........S45

General Information. Unless otherwise noted, all reactions and manipulations were performed in an argon-filled glovebox (VAC DRI-LAB HE 493) or using standard Schlenk techniques. Melting points were measured on a RY-I apparatus and uncorrected. ^{1}H, ^{13}C and ^{31}P NMR spectra were recorded on a Bruker AV 400 spectrometer or a Varian Mercury Plus 400 spectrometer at 400 MHz (^{1}H NMR), 100 MHz (^{13}C NMR) and 162 MHz (^{31}P NMR) in $CDCl_3$. Chemical shifts were reported in ppm down field from internal Me_4Si and external 85% H_3PO_4, respectively. Optical rotations were determined using a Perkin Elmer 341 MC polarimeter. IR spectra were obtained with a Perkin-Elmer spectrometer in KBr disks. HRMS were recorded on IonSpec FT-ICR mass spectrometer with ESI or MALDI resource. Enantiomeric excesses of the asymmetric hydrogenation products were determined by chiral HPLC or GC. HPLC analyses were performed using a Waters 2996 instruments or a Hewlett Packard Model HP 1100 instruments. GC analyses were performed using a Hewlett Packard Model HP 6890 Series instruments. Anhydrous Et_2O, THF and toluene were distilled from sodium benzophenone ketyl, anhydrous CH_2Cl_2, NEt_3, DMF, and pyridine were freshly distilled from calcium hydride under nitrogen atmosphere. Absolute MeOH, EtOH, ^{n}PrOH, ^{n}BuOH, ^{i}PrOH, and ^{t}BuOH were distilled from magnesium under nitrogen atmosphere. Hydrogen gas (99.999%) was purchased from Boc Gas Inc., Tianjin. $[Ir(COD)Cl]_2$ was prepared from $IrCl_3 \cdot 3H_2O$ according to the literature procedure.[1]

1. Preparation and Analytical Data of Iridium Complexes with Chiral Spiro Phosphine-Carboxy Ligands

(*S*)-2d

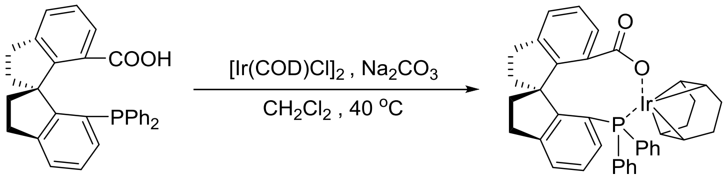

(*S*)-**1d** (*S*)-**2d**

Typical procedure: (*S*)-**1d** (112 mg, 0.250 mmol), $[Ir(COD)Cl]_2$ (84 mg, 0.125 mmol) and Na_2CO_3 (13 mg, 0.125 mmol) were mixed in CH_2Cl_2 (2 mL) in a Schlenk tube under argon atmosphere. The resulting suspension was heated to 40 °C till that the TLC analysis showed no free ligand existed. After cooling to room temperature, the mixture was concentrated under reduced pressure and the residue was purified by a flash column chromatography on silica gel with petroleum ether/ethyl acedate (PE/EA = 1:1, v/v) to offer (*S*)-**2d** (133 mg, yield: 71%) as an orange-yellow solid, mp: 190–191 °C. $[\alpha]_D^{25}$ +277 (*c* 0.5, CH_2Cl_2). ^1H NMR (400 MHz, $CDCl_3$) δ 8.20 (d, *J* = 5.2 Hz, 1H), 7.64 (s, 2H), 7.48–7.24 (m, 10H), 7.12 (t, *J* = 7.6 Hz, 1H), 6.69 (s, 2H), 4.41 (s, 1H), 3.55 (d, *J* = 6.0 Hz, 1H), 3.26 (s, 1H), 2.92–2.75 (m, 5H), 2.43–2.31 (m, 2H), 2.19 (dd, *J* = 15.7 and 9.3 Hz, 1H), 1.91 (d, *J* = 2.4 Hz, 1H), 1.55–1.46 (m, 3H), 1.15–1.14 (m, 1H), 0.97–0.96 (m, 1H), 0.78–0.76 (m, 1H), 0.62–0.60 (m, 1H), 0.12–0.09 (m, 1H); ^{31}P NMR (161 MHz, $CDCl_3$) δ 8.9 (s); ^{13}C NMR (101 MHz, $CDCl_3$) δ 174.5, 150.0, 149.9, 146.4, 146.3, 142.9, 140.5, 136.2, 135.7, 132.33, 132.30, 131.7, 131.1, 130.6, 130.3, 129.0, 128.9, 127.7, 127.6, 127.4, 127.3, 127.2, 127.1, 126.0, 125.6, 119.1, 74.4, 64.5, 64.2, 64.1, 63.0, 61.0, 39.7, 35.0 34.9, 34.2, 31.1, 31.0, 30.6, 30.4, 27.1, 27.0. HRMS (ESI) Calcd for $[C_{38}H_{36}IrNaO_2P, M + Na]^+$: 771.1974, Found: 771.1977.

(*S*)-2b

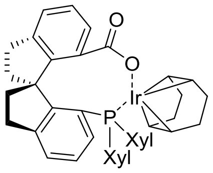

Yield: 75%, orange-yellow solid, mp: 205–206 °C. $[\alpha]_D^{25}$ +318 (*c* 0.5, CH_2Cl_2). ^1H NMR (400 MHz, $CDCl_3$) δ 8.24 (d, *J* = 6.0 Hz, 1H), 7.48 (t, *J* = 8.0 Hz, 1H), 7.37–7.32 (m, 3H), 7.27–7.23 (m, 2H), 7.11 (t, *J* = 8.0 Hz, 2H), 7.04 (s, 2H), 5.73 (s, 1H), 4.40 (td, *J* = 7.6 and 2.2 Hz, 1H), 3.57–3.52 (m, 1H), 3.20 (t, *J* = 7.2 Hz, 1H), 2.92–2.67 (m, 5H), 2.46–2.38 (m, 2H), 2.31 (s, 6H), 2.22 (s, 6H), 2.09 (dd, *J* = 16.0 and 9.2 Hz, 1H), 1.95–1.88 (m, 1H), 1.58–1.42 (m, 3H), 1.23–1.15 (m, 1H), 1.05–0.96 (m, 1H), 0.79–0.73 (m, 1H), 0.60–0.50 (m, 1H), 0.12 (dd, *J* = 22.1 and 10.1 Hz, 1H); ^{31}P NMR (161 MHz, $CDCl_3$) δ 9.9 (s); ^{13}C NMR (101 MHz, $CDCl_3$) δ 174.5, 149.9, 149.8, 146.2, 146.1, 143.2, 140.5, 138.3, 137.0, 136.9, 135.6, 135.2, 133.4, 132.7, 132.42, 132.38, 132.04, 132.02, 130.4, 129.7, 129.3, 127.9, 127.6, 127.1, 127.0, 125.8, 125.2, 118.6, 73.7, 64.1, 63.9, 63.8, 63.0, 61.1, 39.6, 34.83, 34.77, 34.1, 31.1, 30.8, 30.6, 30.3, 27.4, 27.3, 21.5, 21.4. HRMS (ESI) Calcd for $[C_{42}H_{44}IrNaO_2P, M + Na]^+$: 827.2600, Found: 827.2603.

(*S*)-2c

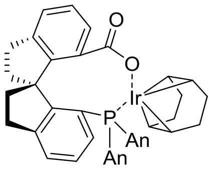

Yield: 78%, orange-yellow solid, mp: 195–196 °C. $[\alpha]_D^{25}$ +273 (*c* 0.5, CH_2Cl_2). ^1H NMR (400 MHz, $CDCl_3$) δ 8.19 (dd, *J* = 5.5 and 2.3 Hz, 1H), 7.57 (t, *J* = 10.4 Hz, 2H), 7.39–7.33 (m, 3H), 7.25–7.23 (m, 1H), 7.13–7.06

(m, 2H), 6.93 (d, *J* = 10.0 Hz, 2H), 6.81 (d, *J* = 10.4 Hz, 2H), 6.59 (s, 1H), 4.45 (dd, *J* = 7.3 and 5.5 Hz, 1H), 3.86 (s, 3H), 3.81 (s, 3H), 3.57–3.52 (m, 1H), 3.23–3.20 (m, 1H), 2.92–2.73 (m, 5H), 2.40–2.18 (m, 3H), 1.95–1.87 (m, 1H), 1.60–1.21 (m, 4H), 1.07–1.01 (m, 1H), 0.87–0.66 (m, 2H), 0.25 (dd, *J* = 22.2 and 10.3 Hz, 1H); ^{31}P NMR (161 MHz, $CDCl_3$) δ 6.4 (s); ^{13}C NMR (101 MHz, $CDCl_3$) δ 174.5, 161.7, 160.2, 149.8, 149.7, 146.2, 146.1, 143.0, 140.6, 137.2, 134.3, 132.1, 132.0, 131.9, 129.6, 128.7, 128.3, 127.4, 127.1, 127.0, 126.9, 125.9, 125.6, 121.3, 120.9, 119.2, 114.5, 113.2, 113.1, 74.4, 64.3, 64.0, 63.6, 63.0, 60.8, 55.5, 55.4, 39.8, 34.8, 34,7, 34.2, 31.3, 31.1, 30.6, 30.5, 27.1, 27.0. HRMS (ESI) Calcd for [$C_{40}H_{40}IrNaO_4P$, M + Na]$^+$: 831.2186, Found: 831.2182.

(*S*)-2a

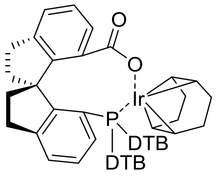

Yield: 83%, orange-yellow solid, mp: 212–213 °C. $[\alpha]_D^{25}$ +200 (*c* 0.5, CH_2Cl_2). ^{1}H NMR (400 MHz, $CDCl_3$) δ 8.17 (d, *J* = 6.8 Hz, 1H), 7.98 (s, 1H), 7.62 (t, *J* = 8.4 Hz, 1H), 7.43 (s, 1H), 7.38 (s, 1H), 7.34–7.26 (m, 3H), 7.15 (t, *J* = 7.2 Hz, 1H), 6.92 (s, 1H), 6.38 (s, 1H), 6.05 (s, 1H), 4.37 (t, *J* = 6.4 Hz, 1H), 3.53 (d, *J* = 7.2 Hz, 1H), 3.34 (s, 1H), 2.94–2.74 (m, 5H), 2.43–2.32 (m, 2H), 2.09 (dd, *J* = 15.8 and 9.3 Hz, 1H), 1.90 (dd, *J* = 9.9 and 4.9 Hz, 1H), 1.54–0.81 (m, 41H), 0.73 (d, *J* = 10.8 Hz, 1H), 0.53–0.37 (m, 2H); ^{31}P NMR (161 MHz, $CDCl_3$) δ 11.1 (s); ^{13}C NMR (101 MHz, $CDCl_3$) δ 174.7, 149.7, 149.6, 146.1, 146.0, 142.5, 140.6, 135.9, 135.4, 132.5, 131.6, 130.6, 130.2, 127.7, 127.4, 127.1, 126.7, 126.6, 125.6, 125.4, 124.7, 121.9, 120.1, 73.0, 64.1, 63.8, 63.3, 63.0, 60.3, 39.7, 34.9, 34.8, 33.8, 31.3, 31.2, 30.8, 30.5, 30.1, 27.4, 27.3. HRMS (ESI) Calcd for [$C_{54}H_{68}IrNaO_2P$, M + Na]$^+$: 995.4478, Found: 995.4480.

2. Preparation and Analytical Data of Unsaturated Carboxylic Acids

The 3-alkyl-3-methylene-carboxylic acids were prepared according to literature procedure.[2] The acids **5a**[2], **5b**[3] and **5k**[2] are known compounds. The other α,β-unsaturated carboxylic acids **7a** and **7b** are commercially available; **7c**,[4] **7d**,[5] **7e**[6] and **7f**[7] were prepared according to the reported procedures.

3-Methylenedecanoic acid (5c)

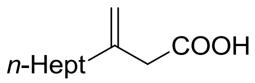

Yield: 80%, colorless oil. ^{1}H NMR (400 MHz, $CDCl_3$) δ 4.96 (s, 1H), 4.93 (s, 1H), 3.08 (s, 2H), 2.12 (t, *J* = 7.6 Hz, 2H), 1.48–1.41 (m, 2H), 1.32–1.28 (m, 8H), 0.88 (t, *J* = 6.4 Hz, 3H); ^{13}C NMR (101 MHz, $CDCl_3$) δ 177.7, 142.1, 114.0, 41.6, 35.9, 31.8, 29.2, 29.1, 27.4, 22.7, 14.1. HRMS (ESI) Calcd for [$C_{11}H_{19}O_2$, M − H]$^-$: 183.1391, Found: 183.1390.

4-Methyl-3-methylenepentanoic acid (5d)

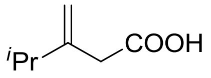

Yield: 76%, colorless oil. ^{1}H NMR (400 MHz, $CDCl_3$) δ 4.99 (s, 1H), 4.92 (s, 1H), 3.10 (s, 2H), 2.39–2.32 (m, 1H), 1.06 (d, *J* = 6.8 Hz, 6H); ^{13}C NMR (101 MHz, $CDCl_3$) δ 178.5, 147.8, 112.0, 40.2, 33.8, 21.4. IR (KBr): ν_{max} 3449, 2957, 2924, 2873, 1709, 1644, 1459, 1293, 1164, 901, 742, 699. HRMS (ESI) Calcd for [$C_7H_{11}O_2$, M − H]$^-$: 127.0765, Found: 127.0766.

4-Ethyl-3-methylenehexanoic acid (5e)

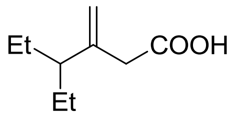

Yield: 70%, colorless oil. ^{1}H NMR (400 MHz, $CDCl_3$) δ 5.05 (s, 1H), 4.96 (s, 1H), 2.97 (s, 2H), 1.96–1.89 (m, 1H), 1.46–1.30 (m, 4H), 0.83 (t, *J* = 7.6 Hz, 6H); ^{13}C NMR (101 MHz, $CDCl_3$) δ 178.4, 143.7, 114.5, 49.7, 38.8,

25.7, 11.7. IR (KBr): ν_{max} 3498, 2963, 2930, 2875, 1711, 1643, 1457, 1293, 900. HRMS (ESI) Calcd for [$C_9H_{15}O_2$, M − H]$^-$: 155.1078, Found: 155.1076.

5-Methyl-3-methylenehexanoic acid (5f)

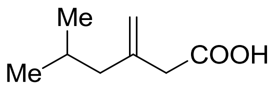

Yield: 78%, colorless oil. ^{1}H NMR (400 MHz, $CDCl_3$) δ 4.97 (s, 1H), 4.94 (s, 1H), 3.06 (s, 2H), 2.01 (d, *J* = 7.2 Hz, 2H), 1.82–1.72 (m, 1H), 0.88 (d, *J* = 6.8 Hz, 6H); ^{13}C NMR (101 MHz, $CDCl_3$) δ 178.3, 140.8, 115.6, 45.5, 41.4, 25.9, 22.4. HRMS (ESI) Calcd for [$C_8H_{13}O_2$, M − H]$^-$: 141.0921, Found: 141.0923.

3-Cyclohexylbut-3-enoic acid (5g)

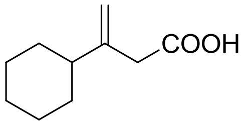

Yield: 70%, colorless oil. ^{1}H NMR (400 MHz, $CDCl_3$) δ 4.97 (s, 1H), 4.92 (s, 1H), 3.10 (s, 2H), 1.98–1.92 (m, 1H), 1.83–1.75 (m, 4H), 1.70–1.66 (m, 1H), 1.33–1.07 (m, 5H); ^{13}C NMR (101 MHz, $CDCl_3$) δ 178.6, 147.1, 112.4, 43.9, 40.6, 32.0, 26.6, 26.2. IR (KBr): ν_{max} 3088, 2927, 2853, 1710, 1643, 1448, 1409, 1294, 1216, 891, 735, 620. HRMS (ESI) Calcd for [$C_{10}H_{15}O_2$, M − H]$^-$: 167.1078, Found: 167.1077.

3-Methylene-5-phenylpentanoic acid (5h)

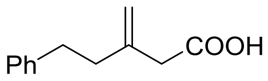

Yield: 72%, colorless oil. ^{1}H NMR (400 MHz, $CDCl_3$) δ 7.30–7.26 (m, 2H), 7.20–7.17 (m, 3H), 5.01 (s, 1H), 4.99 (s, 1H), 3.12 (s, 2H), 2.79 (t, *J* = 7.6 Hz, 2H), 2.45 (t, *J* = 8.4 Hz, 2H); ^{13}C NMR (101 MHz, $CDCl_3$) δ 178.0, 141.5, 141.2, 128.4, 128.3, 126.0, 114.8, 41.9, 37.5, 33.9. IR (KBr): ν_{max} 3643, 3084, 3027, 2929, 2857, 1709, 1648, 1409, 1294, 1217, 902, 744, 698. HRMS (ESI) Calcd for [$C_{12}H_{13}O_2$, M − H]$^-$: 189.0921, Found: 189.0923.

7-Methoxy-3-methyleneheptanoic acid (5i)

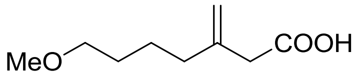

Yield: 75%, colorless oil. ^{1}H NMR (400 MHz, $CDCl_3$) δ 4.95 (s, 1H), 4.94 (s, 1H), 3.39 (t, *J* = 6.4 Hz, 2H), 3.33 (s, 3H), 3.07 (s, 2H), 2.14 (t, *J* = 7.6 Hz, 2H), 1.62–1.47 (m, 4H); ^{13}C NMR (101 MHz, $CDCl_3$) δ 177.5, 141.7, 114.3, 72.6, 58.5, 41.6, 35.6, 29.1, 23.9. IR (KBr): ν_{max} 3456, 2938, 2869, 1712, 1647, 1446, 1391, 1114, 899, 739. HRMS (ESI) Calcd for [$C_9H_{15}O_3$, M − H]$^-$: 171.1027, Found: 171.1029.

7-Methyl-3-methyleneoct-6-enoic acid (5j)

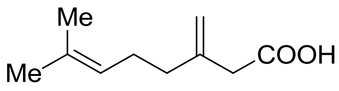

Yield: 62%, colorless oil. ^{1}H NMR (400 MHz, $CDCl_3$) δ 5.12–5.07 (m, 1H), 4.98 (s, 1H), 4.96 (s, 1H), 3.09 (s, 2H), 2.15–2.14 (m, 4H), 1.68 (s, 3H), 1.61 (s, 3H); ^{13}C NMR (101 MHz, $CDCl_3$) δ 178.1, 141.7, 132.1, 123.5, 114.3, 41.8, 35.8, 26.1, 25.7, 17.7. HRMS (ESI) Calcd for [$C_{10}H_{15}O_2$, M − H]$^-$: 167.1078, Found: 167.1080.

3. Asymmetric Hydrogenation and Analytical Data of Products

General procedure of hydrogention

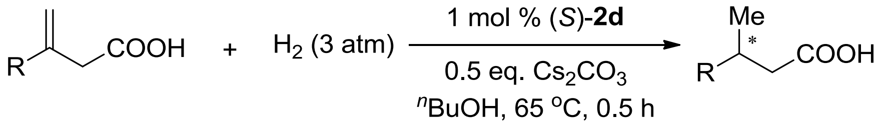

A hydrogenation tube was charged with a stir bar, β-alkyl-β,γ-unsaturated acids **5** (0.5 mmol), catalyst (*S*)-**2d** (3.7 mg, 0.005 mmol), Cs_2CO_3 (82 mg, 0.25 mmol) in an argon-filled glovebox. Then 2 mL ^{n}BuOH was injected into the hydrogenation tube by a syringe with stirring. The hydrogenation tube was put into an autoclave. The argon in the autoclave was replaced with hydrogen for 3 times, and was finally charged with hydrogen to 3 atm. The reaction mixture was stirred at 65 °C for specified time before releasing the hydrogen.

After releasing hydrogen, the reaction mixture was added with 20 mg NaOH and concentrated under reduced pressure. The mixture was added 25 mL water and washed with Et_2O. The aqueous layer was acidified with conc. HCl, and extracted with Et_2O. The organic layer was dried with anhydrous Na_2SO_4. The conversion of substrate was determined by ^{1}H NMR analysis. The crude product was purified by a flash chromatography on silica gel column to give pure product **6**. The acid **6** (0.5 mmol) was reacted with aniline (50 μL, 0.55 mmol) in the presence of *N*,*N*-4-dimethylaminopyridine (DMAP, 4 mg, 0.033 mmol) and dicyclohexylcarbodiimide (DCC, 110 mg, 0.53 mmol) in 2.0 mL THF for 30 min. The filtrate was concentrated under reduced pressure and the residue passed through a flash chromatography on Al_2O_3 column with PE/EA (4:1, v/v) as eluent to afford the corresponding amide. The ee value of amide was determined by HPLC or GC.

(*R*)-3-Methylheptanoic acid (6a)[2]

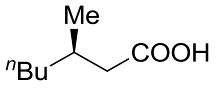

Yield: 99%, colorless oil. 93% ee (*R*), $[\alpha]_D^{25}$ +4.70 (*c* 0.4, CH_2Cl_2), HPLC condition for corresponding amide: Chiralpak AD-H column (25 cm × 0.46 cm ID), *n*-hexane/2-propanol = 85:15, 1.0 mL/min, 254 nm UV detector, t_R = 5.43 min for (*S*)-enantiomer and t_R = 5.94 min for (*R*)-enantiomer. ^{1}H NMR (400 MHz, $CDCl_3$) δ 2.35 (dd, *J* = 15.2 and 6.0 Hz, 1H), 2.14 (dd, *J* = 14.8 and 8.0 Hz, 1H), 1.99–1.89 (m, 1H), 1.37–1.17 (m, 6H), 0.96 (d, *J* = 6.8 Hz, 3H), 0.89 (t, *J* = 6.8 Hz, 3H). IR (KBr): ν_{max} 3450, 2960, 2925, 2861, 1708, 1413, 1292, 936.

(*R*)-3-Methylpentanoic acid (6b)[8]

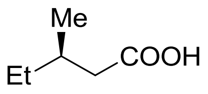

Yield: 99%, colorless oil. 86% ee (*R*), $[\alpha]_D^{25}$ –5.52 (*c* 1.0, CH_3OH), HPLC condition for corresponding amide: Chiralpak AD-H column (25 cm × 0.46 cm ID), *n*-hexane/2-propanol = 85:15, 1.0 mL/min, 254 nm UV detector, t_R = 6.10 min for (*S*)-enantiomer and t_R = 6.46 min for (*R*)-enantiomer. ^{1}H NMR (400 MHz, $CDCl_3$) δ 2.39 (dd, *J* = 15.2 and 6.0 Hz, 1H), 2.17 (dd, *J* = 15.2 and 8.4 Hz, 1H), 1.96–1.88 (m, 1H), 1.48–1.36 (m, 1H), 1.34–1.25 (m, 1H), 1.00 (d, *J* = 6.4 Hz, 3H), 0.93 (t, *J* = 7.6 Hz, 3H).

3-Methyldecanoic acid (6c)

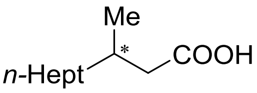

Yield: 99%, colorless oil. 94% ee, $[\alpha]_D^{25}$ +7.62 (*c* 1.0, $CHCl_3$), HPLC condition for corresponding amide: Chiralpak AD-H column (25 cm × 0.46 cm ID), *n*-hexane/2-propanol = 90:10, 1.0 mL/min, 254 nm UV detector, t_R = 6.78 min for the minor isomer and t_R = 7.70 min for the major isomer. ^1H NMR (400 MHz, $CDCl_3$) δ 2.35 (dd, *J* = 14.8 and 5.6 Hz, 1H), 2.14 (dd, *J* = 14.8 and 8.0 Hz, 1H), 1.97–1.94 (m, 1H), 1.31–1.21 (m, 12H), 0.96 (d, *J* = 6.8 Hz, 3H), 0.88 (t, *J* = 6.4 Hz, 3H); ^{13}C NMR (101 MHz, $CDCl_3$) δ 180.1, 41.6, 36.7, 31.9, 30.2, 29.7, 29.3, 26.9, 22.7, 19.7, 14.1. HRMS (ESI) Calcd for $[C_{11}H_{21}O_2, M-H]^-$: 185.1547, Found: 185.1545.

(*S*)-3,4-Dimethylpentanoic acid (6d)[9]

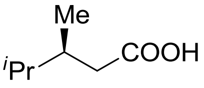

Yield: 98%, colorless oil. 97% ee (*S*), $[\alpha]_D^{25}$ −7.92 (*c* 1.4, C_6H_6), HPLC condition for corresponding amide: Chiralpak AD-H column (25 cm × 0.46 cm ID), *n*-hexane/2-propanol = 90:10, 1.0 mL/min, 254 nm UV detector, t_R = 8.76 min for (*R*)-enantiomer and t_R = 10.04 min for (*S*)-enantiomer. ^1H NMR (400 MHz, $CDCl_3$) δ 2.38 (dd, *J* = 14.8 and 5.2 Hz, 1H), 2.09 (dd, *J* = 14.8 and 9.2 Hz, 1H), 1.90–1.84 (m, 1H), 1.64–1.56 (m, 1H), 0.91–0.84 (m, 9H). IR (KBr): ν_{max} 3670, 2959, 2875, 2659, 1709, 1462, 1302, 1119, 929.

4-Ethyl-3-methylhexanoic acid (6e)

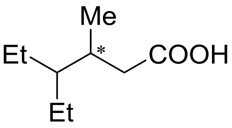

Yield: 99%, colorless oil. 98% ee, $[\alpha]_D^{25}$ +9.76 (*c* 1.0, $CHCl_3$), HPLC condition for corresponding amide: Chiralpak AD-H column (25 cm × 0.46 cm ID), *n*-hexane/2-propanol = 85:15, 1.0 mL/min, 254 nm UV detector, t_R = 5.43 min for the minor isomer and t_R = 5.94 min for the major isomer. ^1H NMR (400 MHz, $CDCl_3$) δ 2.36–2.31 (m, 1H), 2.16–2.08 (m, 2H), 1.33–1.22 (m, 5H), 0.90–0.86 (m, 9H); ^{13}C NMR (101 MHz, $CDCl_3$) δ 180.4, 45.9, 39.1, 31.2, 23.0, 22.3, 15.9, 12.2, 12.1. IR (KBr): ν_{max} 3788, 2964, 2930, 2875, 1708, 1412, 1296, 1203, 930, 745. HRMS (ESI) Calcd for $[C_9H_{17}O_2, M-H]^-$: 157.1234, Found: 157.1223.

(*R*)-3,5-Dimethylhexanoic acid (6f)

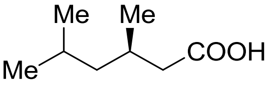

Yield: 99%, colorless oil. 96% ee (*R*), $[\alpha]_D^{25}$ +12.8 (*c* 5.0, $CHCl_3$),[10] HPLC condition for corresponding amide: Chiralpak AD-H column (25 cm × 0.46 cm ID), *n*-hexane/2-propanol = 95:5, 1.0 mL/min, 254 nm UV detector, t_R = 15.93 min for (*S*)-enantiomer and t_R = 16.61 min for (*R*)-enantiomer. ^1H NMR (400 MHz, $CDCl_3$) δ 2.32 (dd, *J* = 14.4 and 5.2 Hz, 1H), 2.13–1.98 (m, 2H), 1.68–1.58 (m, 1H), 1.18–1.05 (m, 2H), 0.94 (d, *J* = 6.4 Hz, 3H), 0.87 (t, *J* = 6.8 Hz, 6H); ^{13}C NMR (101 MHz, $CDCl_3$) δ 180.0, 46.2, 42.2, 27.9, 25.2, 23.2, 22.1, 19.7. HRMS (ESI) Calcd for $[C_8H_{15}O_2, M-H]^-$: 143.1078, Found: 143.1076.

3-Cyclohexylbutanoic acid (6g)[11]

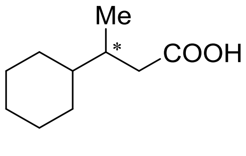

Yield: 99%, colorless oil. 98% ee, $[\alpha]_D^{25}$ +9.97 (*c* 1.0, $CHCl_3$), HPLC condition for corresponding amide: Chiralpak AD-H column (25 cm × 0.46 cm ID), *n*-hexane/2-propanol = 85:15, 1.0 mL/min, 254 nm UV detector, t_R = 6.32 min for the minor isomer and t_R = 7.06 min for the

major isomer. ^{1}H NMR (400 MHz, $CDCl_3$) δ 2.41 (dd, J = 14.8 and 4.8 Hz, 1H), 2.08 (dd, J = 14.8 and 9.6 Hz, 1H), 1.87–1.85 (m, 1H), 1.75–1.73 (m, 2H), 1.65–1.62 (m, 3H), 1.25–0.94 (m, 6H), 0.91 (d, J = 6.4 Hz, 3H). IR (KBr): ν_{max} 3480, 2925, 2853, 1708, 1449, 1288, 938.

(*R*)-3-Methyl-5-phenylpentanoic acid (6h)[12]

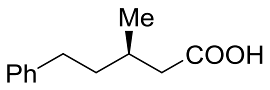

Yield: 99%, colorless oil. 93% ee (*R*), $[\alpha]_D^{25}$ +67.6 (*c* 1.0, C_6H_6), HPLC condition for corresponding amide: Chiralpak AD-H column (25 cm × 0.46 cm ID), *n*-hexane/2-propanol = 85:15, 1.0 mL/min, 254 nm UV detector, t_R = 7.52 min for (*S*)-enantiomer and t_R = 8.35 min for (*R*)-enantiomer. ^{1}H NMR (400 MHz, $CDCl_3$) δ 7.31–7.26 (m, 2H), 7.21–7.19 (m, 3H), 2.73–2.57 (m, 2H), 2.43 (dd, J = 15.2 and 6.0 Hz, 1H), 2.23 (dd, J = 14.8 and 8.0 Hz, 1H), 2.09–2.00 (m, 1H), 1.76–1.67 (m, 1H), 1.60–1.51 (m, 1H), 1.06 (d, J = 6.8 Hz, 3H). IR (KBr): ν_{max} 3479, 3028, 2961, 2926, 1707, 1454, 1299, 746, 698.

7-Methoxy-3-methylheptanoic acid (6i)

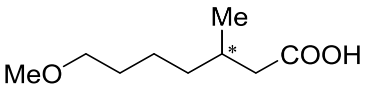

Yield: 99%, colorless oil. 93% ee, $[\alpha]_D^{25}$ +5.31 (*c* 1.0, $CHCl_3$), HPLC condition for corresponding amide: Chiralpak AD-H column (25 cm × 0.46 cm ID), *n*-hexane/2-propanol = 85:15, 1.0 mL/min, 254 nm UV detector, t_R = 23.02 min for the minor isomer and t_R = 24.18 min for the major isomer. ^{1}H NMR (400 MHz, $CDCl_3$) δ 3.37 (t, J = 6.8 Hz, 2H), 3.32 (s, 3H), 2.32 (dd, J = 14.8 and 5.6 Hz, 1H), 2.11 (dd, J = 14.8 and 8.0 Hz, 1H), 1.94–1.93 (m, 1H), 1.56–1.52 (m, 2H), 1.40–1.21 (m, 4H), 0.97 (d, J = 7.2 Hz, 3H); ^{13}C NMR (101 MHz, $CDCl_3$) δ 179.4, 72.8, 58.5, 41.8, 36.5, 30.2, 29.7, 23.5, 19.6. IR (KBr): ν_{max} 3468, 2932, 2868, 1709, 1460, 1291, 1192, 1117, 936, 739. HRMS (ESI) Calcd for $[C_9H_{17}O_3, M - H]^-$: 173.1183, Found: 173.1182.

(*R*)-3,7-Dimethyloct-6-enoic acid (6j)[13]

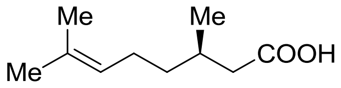

Yield: 99%, colorless oil. 94% ee (*R*), $[\alpha]_D^{25}$ +9.31 (*c* 1.0, $CHCl_3$), HPLC condition for corresponding amide: Chiralpak AD-H column (25 cm × 0.46 cm ID), *n*-hexane/2-propanol = 90:10, 1.0 mL/min, 254 nm UV detector, t_R = 15.53 min for (*S*)-enantiomer and t_R = 16.73 min for (*R*)-enantiomer. ^{1}H NMR (400 MHz, $CDCl_3$) δ 5.09 (t, J = 7.2 Hz, 1H), 2.36 (dd, J = 15.2 and 6.0 Hz, 1H), 2.15 (dd, J = 14.8 and 8.4 Hz, 1H), 2.07–1.95 (m, 3H), 1.68 (s, 3H), 1.60 (s, 3H), 1.41–1.34 (m, 1H), 1.29–1.20 (m, 1H), 0.98 (d, J = 6.4 Hz, 3H).

(*S*)-2-Methyl-3-phenylpropionic acid (8a)[14]

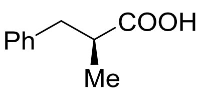

Yield: 99%, colorless oil. 99.4% ee (*S*), $[\alpha]_D^{25}$ +31.2 (*c* 0.8, $CHCl_3$), HPLC condition for corresponding amide: Chiralpak AS column (25 cm × 0.46 cm ID), *n*-hexane/2-propanol = 95:5, 1.0 mL/min, 254 nm UV detector, t_R = 14.53 min for (*S*)-enantiomer and t_R = 17.72 min for (*R*)-enantiomer. ^{1}H NMR (400 MHz, $CDCl_3$) δ 7.30–7.17 (m, 5H), 3.08 (dd, J = 17.2 and 8.0 Hz, 1H), 2.80−2.63 (m, 2H), 1.17 (d, J = 9.2 Hz, 3H). IR (KBr): ν_{max} 3664, 3062, 2972, 2927, 2855, 1707, 1496, 1236, 910, 742, 699.

(*S*)-2-Methylbutanoic acid (8b)[15]

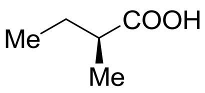

Yield: 98%, colorless oil. 97% ee (*S*), $[\alpha]_D^{25}$ +20.7 (*c* 0.8, C_2H_5OH), HPLC condition for corresponding amide: Chiralpak AD-H column (25 cm × 0.46 cm ID), *n*-hexane/2-propanol = 95:5, 1.0 mL/min, 254 nm UV detector, t_R = 17.23 min for (*S*)-enantiomer and t_R = 18.94 min for (*R*)-enantiomer. ^{1}H NMR (400 MHz, $CDCl_3$)

δ 2.45–2.34 (m, 1H), 1.76–1.64 (m, 1H), 1.54–1.45 (m, 1H), 1.17 (d, *J* = 9.2 Hz, 3H), 0.94 (t, *J* = 10.0 Hz, 3H). IR (KBr): ν_{max} 3670, 2962, 2928, 2872, 1708, 1550, 1459, 1024.

(*R*)-2-Phenylbutanoic acid (8c)[16]

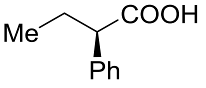

Yield: 98%, white solid, mp: 40−42 °C. 94% ee (*R*), $[\alpha]_D^{25}$ –70.4 (*c* 1.0, $CHCl_3$), HPLC condition for corresponding amide: Chiralpak AD-H column (25 cm × 0.46 cm ID), *n*-hexane/2-propanol = 80:20, flow rate = 1.0 mL/min, 254 nm UV detector, t_R = 6.41 min for (*S*)-enantiomer and t_R = 7.67 min for (*R*)-enantiomer. ^{1}H NMR (400 MHz, $CDCl_3$) δ 7.33–7.22 (m, 5H), 3.45 (t, *J* = 8.0 Hz, 1H), 2.15–2.04 (m, 1H), 1.86–1.75 (m, 1H), 0.90 (t, *J* = 7.6 Hz, 3H).

(*S*)-2-(Benzyloxy)-3-phenylpropionic acid (8d)[5]

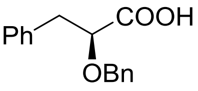

Yield: 98%, white solid, mp: 59−60 °C. 98% ee (*S*), $[\alpha]_D^{20}$ –83.2 (*c* 2.2, C_2H_5OH),[17] HPLC condition for corresponding amide: Chiralpak AD-H column (25 cm × 0.46 cm ID), *n*-hexane/2-propanol = 80:20, flow rate = 1.0 mL/min, 254 nm UV detector, t_R = 10.19 min for (*S*)-enantiomer and t_R = 10.91 min for (*R*)-enantiomer. ^{1}H NMR (400 MHz, $CDCl_3$) δ 7.33−7.26 (m, 8H), 7.16−7.14 (m, 2H), 4.67 (d, *J* = 15.6 Hz, 1H), 4.40 (d, *J* = 15.6 Hz, 1H), 4.18 (dd, *J* = 11.2 and 5.6 Hz, 1H), 3.20–3.01 (m, 2H).

(*R*)-2-(4-Isobutylphenyl)propionic acid [(*R*)-ibuprofen] (8e)[18]

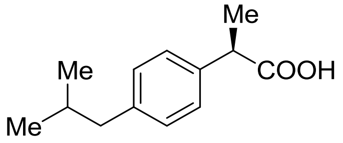

Yield: 99%, white solid, mp: 53–54 °C. 96% ee (*R*), $[\alpha]_D^{25}$ −51.4 (*c* 2.0, C_2H_5OH), GC condition for corresponding methyl ester: Coating CP Chirasil-DEX CB, CP7502, df = 0.25 μm, 0.25 mm i.d. x 25 m, carrier gas: N_2 (1.3 mL/min), inject temperature: 230 °C, initial temperature: 120 °C, hold 60 min then temperature programmed, programming rate: 1.0 °C /min, final temperature: 180 °C. t_R = 38.26 min for (*S*)-enantiomer and t_R = 38.88 min for (*R*)-enantiomer. ^{1}H NMR (400 MHz, $CDCl_3$) δ 7.22 (d, *J* = 8.0 Hz, 2H), 7.11 (d, *J* = 8.0 Hz, 2H), 3.71 (q, *J* = 7.2 Hz, 1H), 2.45 (d, *J* = 6.8 Hz, 2H), 1.90−1.80 (m, 1H), 1.50 (d, *J* = 7.2 Hz, 3H), 0.90 (d, *J* = 6.8 Hz, 6H).

(*R*)-2-Methylheptanoic acid (8f)[19]

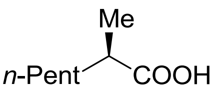

Yield: 97%, colorless oil. 99.3% ee (*R*), $[\alpha]_D^{25}$ –16.8 (*c* 0.6, C_2H_5OH), HPLC condition for corresponding amide: Chiralpak AD-H column (25 cm × 0.46 cm ID), *n*-hexane/2-propanol = 95:5, flow rate = 1.0 mL/min, 254 nm UV detector, t_R = 11.46 min for (*S*)-enantiomer and t_R = 12.43 min for (*R*)-enantiomer. ^{1}H NMR (400 MHz, $CDCl_3$) δ 2.49−2.41 (m, 1H), 1.72−1.63 (m, 1H), 1.47−1.38 (m, 1H), 1.36−1.24 (m, 6H), 1.17 (d, *J* = 7.2 Hz, 3H), 0.88 (t, *J* = 6.4 Hz, 3H).

4. Total Synthesis of (*S*)-14-Methyloctadec-1-ene

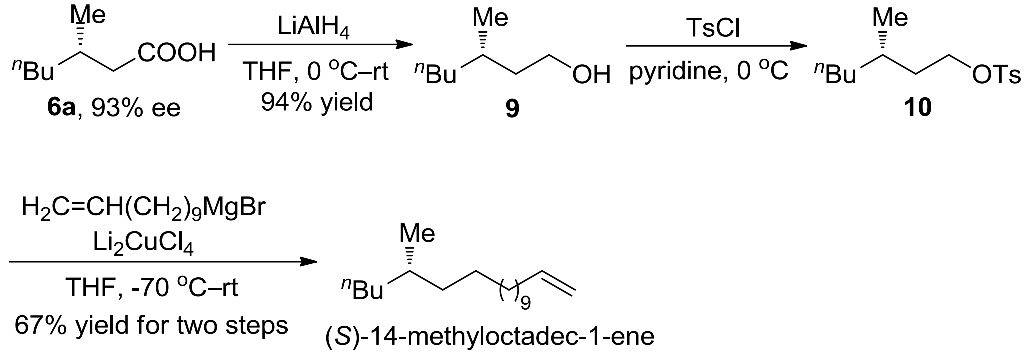

(*S*)-3-Methylheptan-1-ol (9)[20]

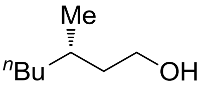

A solution of acid **6a** (72 mg, 0.50 mmol) in 1 mL of anhydrous THF was added to a stirred suspension of $LiAlH_4$ (38 mg, 1.0 mmol) in 1 mL of anhydrous THF at 0 ℃. The reaction mixture was warmed to room temperature, and stirred for 2 h. After cooling to 0 ℃, 1 mL of water and 1 mL of aq. NaOH (10% solution) were added with stirring. The organic layer was separated, and the aqueous layer was extracted with Et_2O. The combined organic layer was dried with Na_2SO_4 and concentrated under reduced pressure. The residue was purified by a flash column chromatography on silica gel with PE/EA (4:1) to offer **9** (61 mg, 94%) as a colorless oil. $[\alpha]_D^{25}$ −2.73 (*c* 3.0, $CHCl_3$). ^1H NMR (400 MHz, $CDCl_3$) δ 3.69–3.63 (m, 2H), 1.66–1.54 (m, 2H), 1.37−1.24 (m, 7H), 0.89 (t, *J* = 6.8 Hz, 3H), 0.86 (t, *J* = 6.6 Hz, 3H).

(*S*)-3-Methylheptyl 4-methylbenzenesulfonate (10)[20]

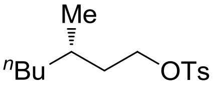

Tosyl chloride (70 mg, 0.38 mmol) was added to a solution of alcohol **9** (50 mg, 0.35 mmol) in 1 mL of anhydrous pyridine at 0 ℃ and the mixture was stirred for 16 h at the same temperature. The mixture was added 20 mL of Et_2O and washed successively with brine, saturated solution of $CuSO_4$ and $NaHCO_3$, and brine. The organic phase was dried with Na_2SO_4, and concentrated to give tosylate **10**. The product was used in the next step without further purification.

(*S*)-14-Methyloctadec-1-ene[20]

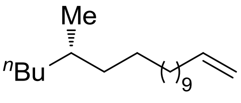

A solution of tosylate **10** in 3 mL of anhydrous THF was added dropwise to a stirred solution of the Grignard reagent (0.38 mmol) at 0 °C. A solution of Li_2CuCl_4 (0.2 N, 0.10 mL) in anhydrous THF was added. The reaction mixture was stirred at –70 ℃ for 1 h, at –10 ℃ for 2 h, and at 25 ℃ for 2 h, poured into a cooled saturated solution of NH_4Cl, and extracted with Et_2O. The combined extract was washed successively with saturated solution of $NaHCO_3$ and brine, dried with Na_2SO_4, and concentrated under reduced pressure. The residue was purified by a flash column chromatography on silica gel with PE to offer (*S*)-14-methyloctadec-1-ene (62 mg, 67% for two steps) as a colorless oil. $[\alpha]_D^{25}$ +1.18 (*c* 5.0, $CHCl_3$). ^1H NMR (400 MHz, $CDCl_3$) δ 5.77–5.71 (m, 1H), 4.94–4.84 (m, 2H), 1.98–1.96 (m, 2H), 1.73–1.71 (m, 1H), 1.53–1.51 (m, 2H), 1.30–1.18 (m, 24H), 0.81–0.79 (m, 6H).

5. X-ray Diffraction Analysis of (*S*)-2d

The fine yellow crystals of (*S*)-**2d** suitable for the X-ray diffraction analyses grow slowly on the interface of a solution of (*S*)-**2d** (30 mg) in dichloromethane (0.5 mL) and *n*-hexane (1.5 mL).

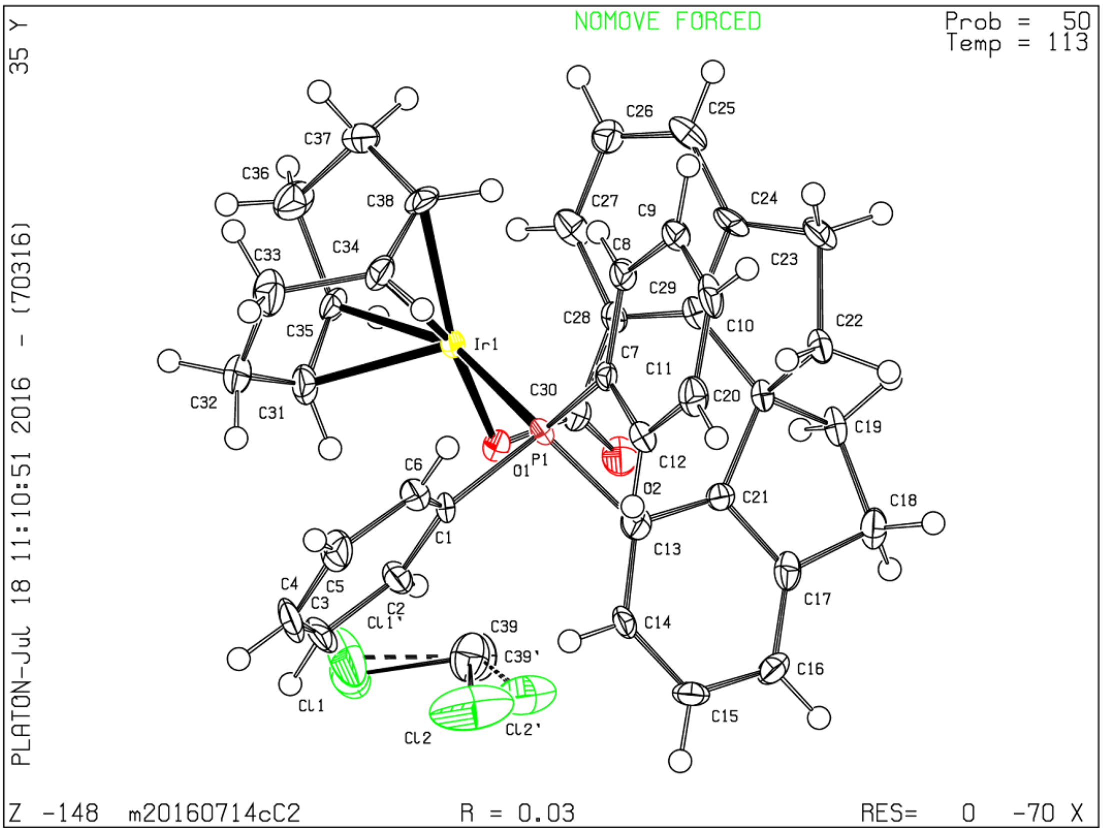

Table S1. Crystal data and structure refinement for (*S*)-2d

Empirical formula	C39 H36 Cl2 Ir O2 P
Moiety formula	$C_{39}H_{36}Cl_2IrO_2P$
Formula weight	830.75
Temperature	113(2) K
Wavelength	0.71073 Å
Crystal system	Monoclinic
Space group	C2
Unit cell dimensions	a = 19.385(3) Å alpha = 90°<br>b = 16.220(3) Å beta = 107.035(18)°<br>c = 10.9215(15) Å gamma = 90°
Volume	3283.2(10) Å^3
Z	4
Calculated density	1.681 Mg/m^3
Absorption coefficient	4.313 mm^{-1}

F(000)	1648
Crystal size	0.20 x 0.18 x 0.12 mm
Theta range for data collection	3.18 to 25.01^{o}
Limiting indices	-23<=h<=23, -19<=k<=18, -12<=l<=12
Reflections collected / unique	17815 / 5559 [R(int) = 0.0734]
Completeness to theta = 25.01	99.7 %
Absorption correction	Semi-empirical from equivalents
Max. and min. transmission	0.6256 and 0.4792
Refinement method	Full-matrix least-squares on F^2
Data / restraints / parameters	5559 / 71 / 434
Goodness-of-fit on F^2	1.041
Final R indices [I>2sigma(I)]	R_1 = 0.0317, wR_2 = 0.0748
R indices (all data)	R_1 = 0.0321, wR_2 = 0.0750
Absolute structure parameter	0.010(7)
Largest diff. peak and hole	2.781 and -1.354 e.Å^{-3}

6. NMR Spectra of New Compounds

(*S*)-2a

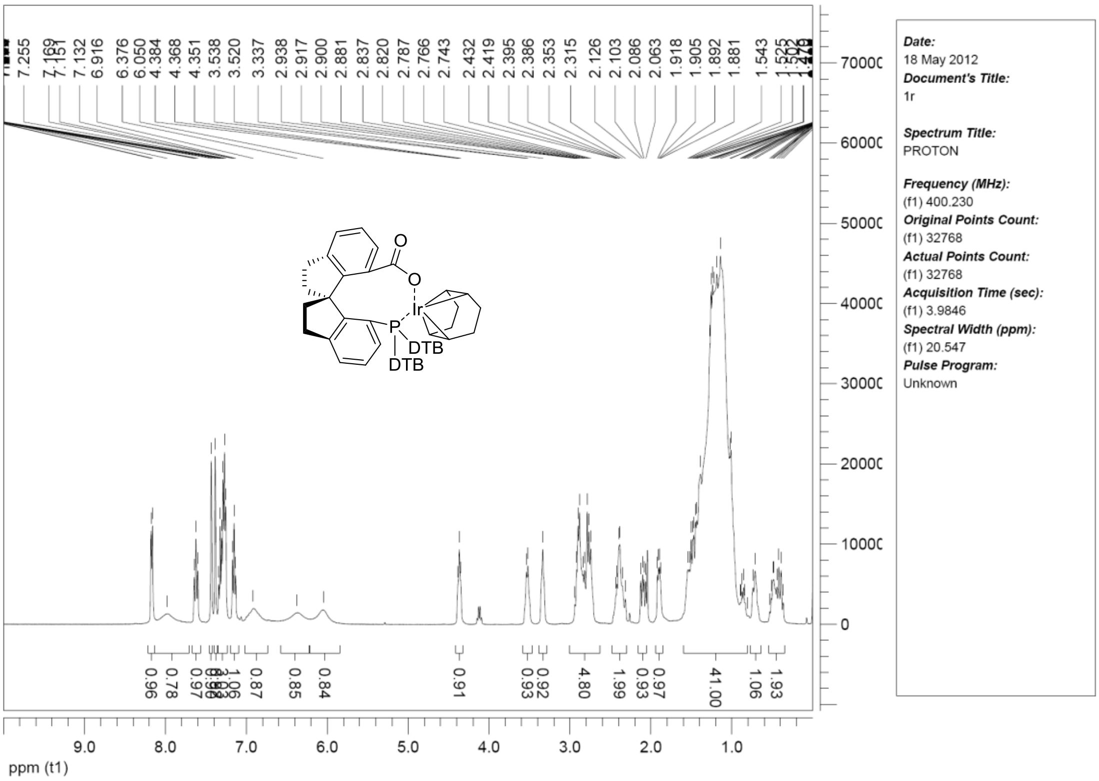

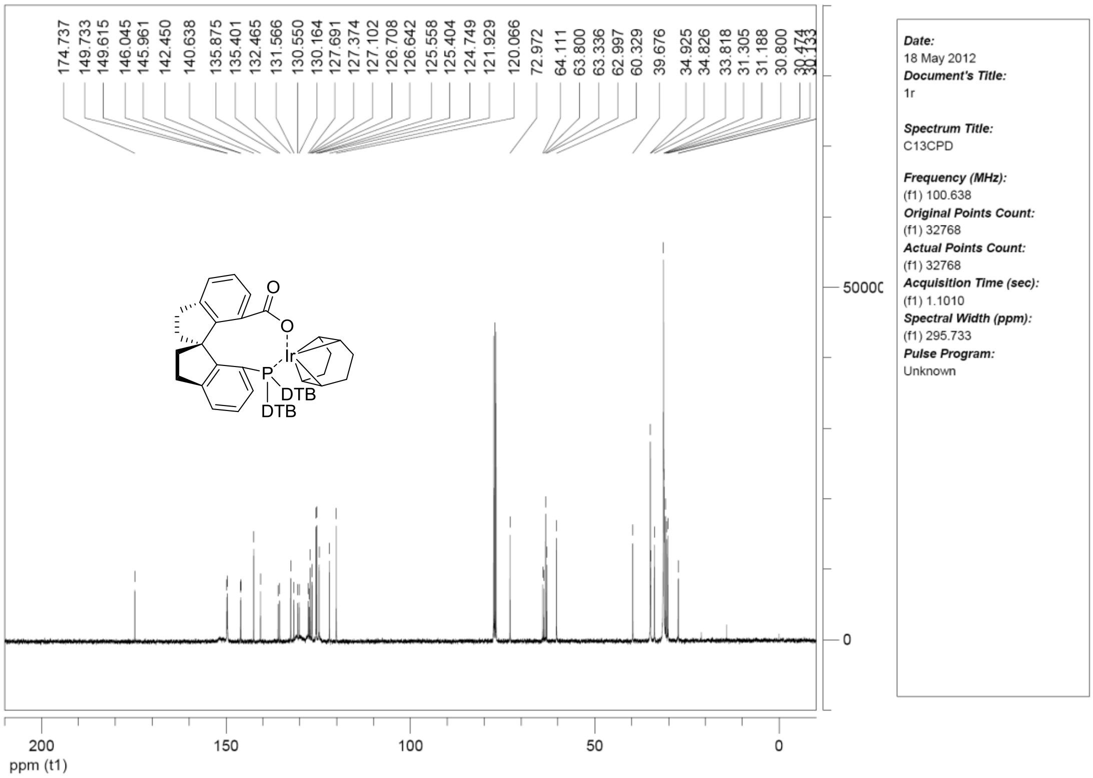

(*S*)-2b

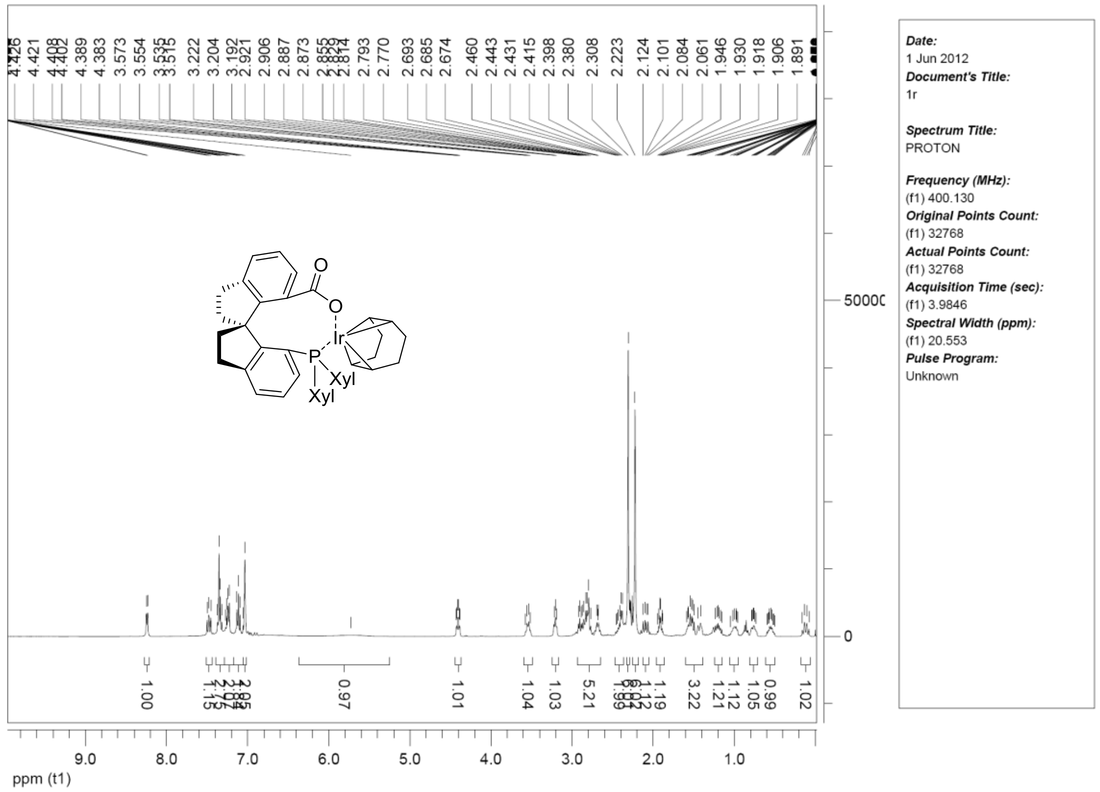
Date:
1 Jun 2012
Document's Title:
1r
Spectrum Title:
PROTON
Frequency (MHz):
(f1) 400.130
Original Points Count:
(f1) 32768
Actual Points Count:
(f1) 32768
Acquisition Time (sec):
(f1) 3.9846
Spectral Width (ppm):
(f1) 20.553
Pulse Program:
Unknown
Ir
P
Xyl
Xyl
O
O
ppm (t1)

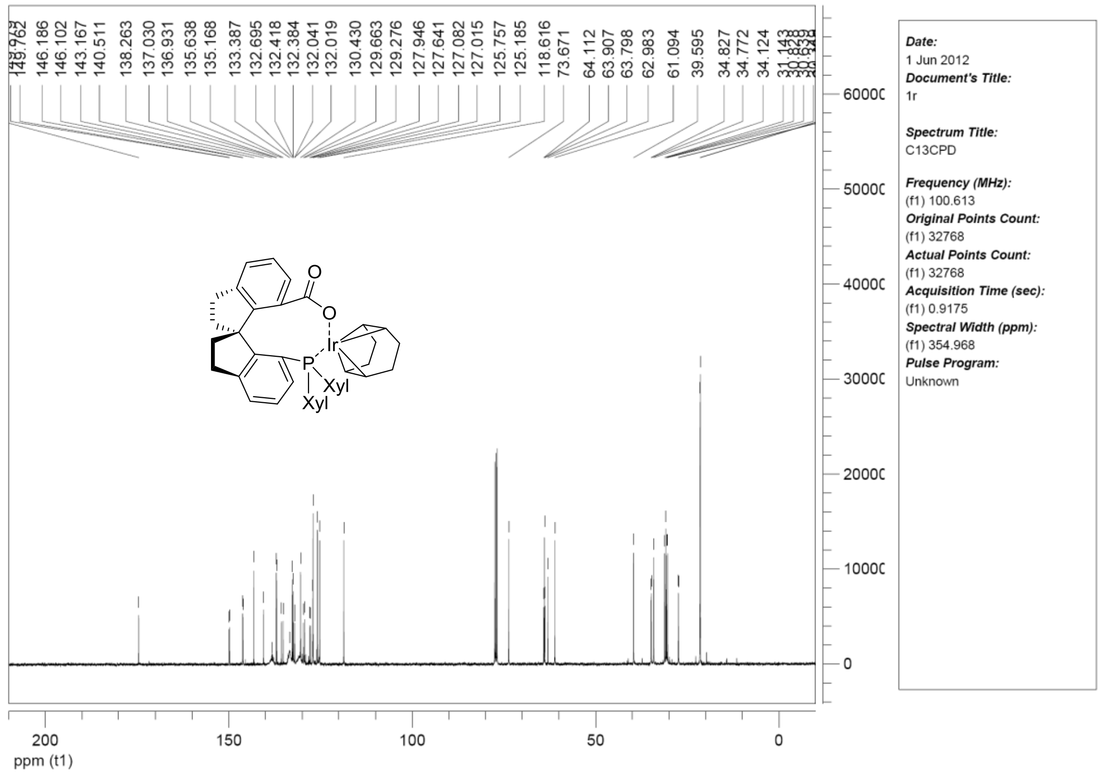
Date:
1 Jun 2012
Document's Title:
1r
Spectrum Title:
C13CPD
Frequency (MHz):
(f1) 100.613
Original Points Count:
(f1) 32768
Actual Points Count:
(f1) 32768
Acquisition Time (sec):
(f1) 0.9175
Spectral Width (ppm):
(f1) 354.968
Pulse Program:
Unknown
Ir
P
Xyl
Xyl
O
O
ppm (t1)

(*S*)-2c

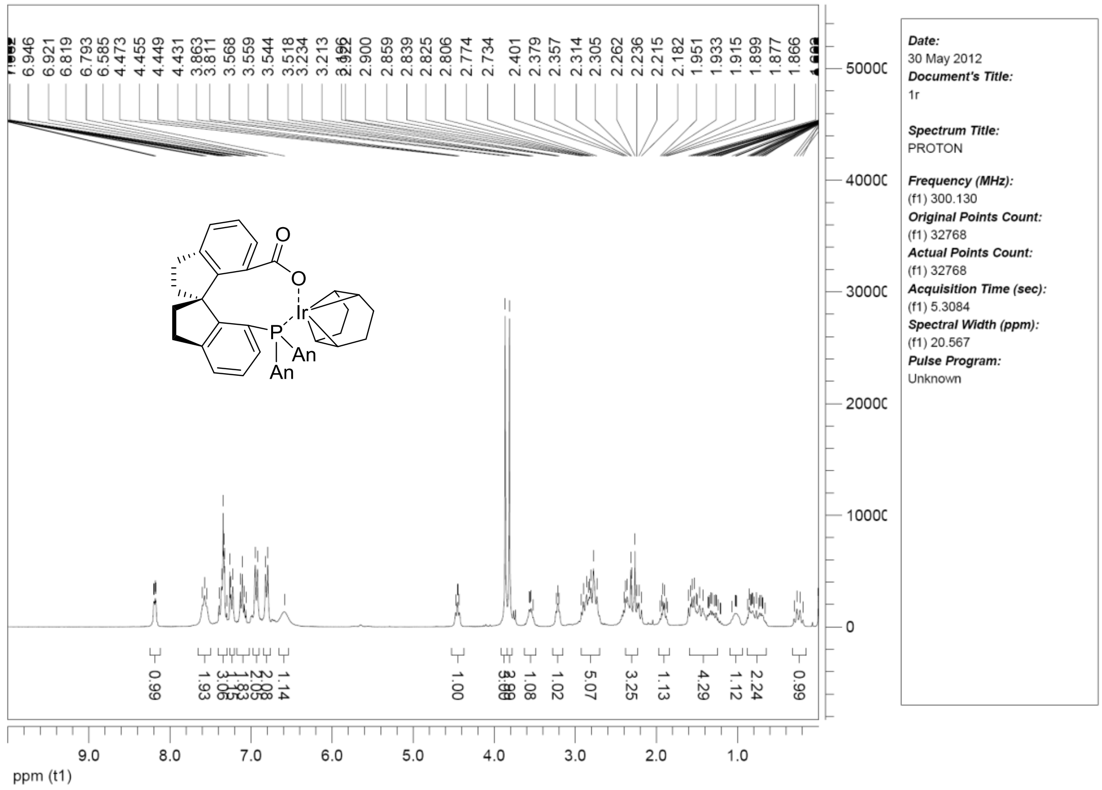
Date:
30 May 2012
Document's Title:
1r
Spectrum Title:
PROTON
Frequency (MHz):
(f1) 300.130
Original Points Count:
(f1) 32768
Actual Points Count:
(f1) 32768
Acquisition Time (sec):
(f1) 5.3084
Spectral Width (ppm):
(f1) 20.567
Pulse Program:
Unknown
ppm (t1)
O
O
Ir
P
An
An

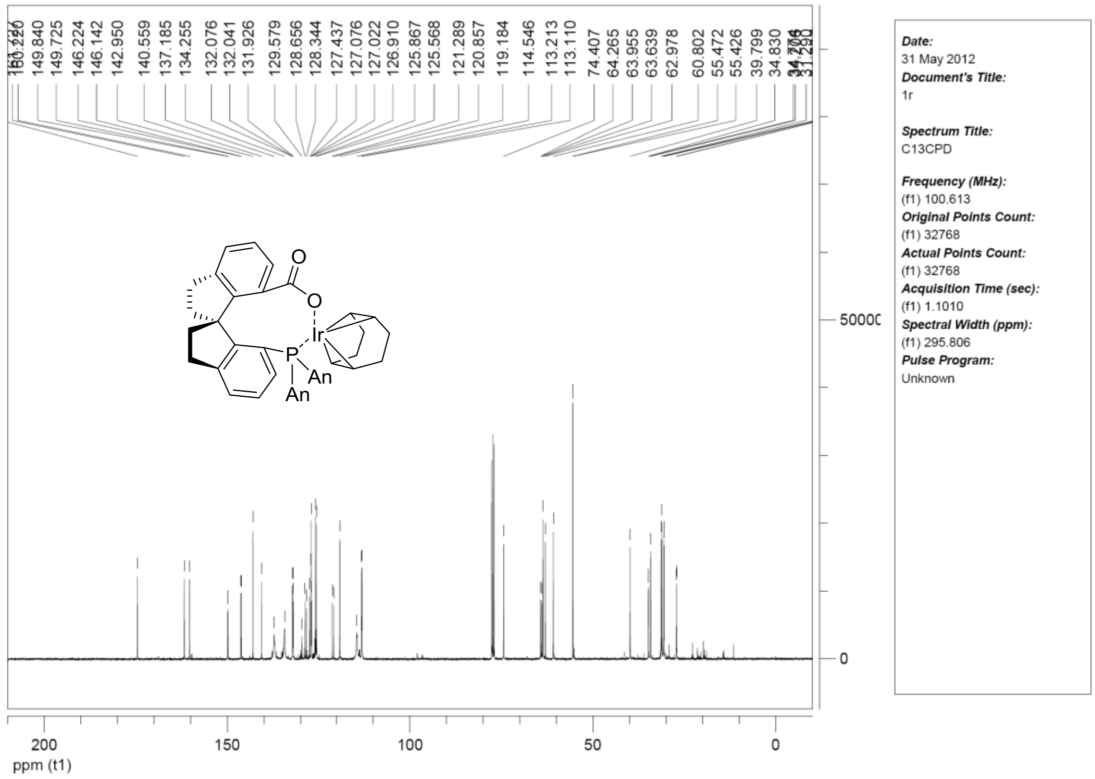
Date:
31 May 2012
Document's Title:
1r
Spectrum Title:
C13CPD
Frequency (MHz):
(f1) 100.613
Original Points Count:
(f1) 32768
Actual Points Count:
(f1) 32768
Acquisition Time (sec):
(f1) 1.1010
Spectral Width (ppm):
(f1) 295.806
Pulse Program:
Unknown
ppm (t1)
O
O
Ir
P
An
An

(*S*)-2d

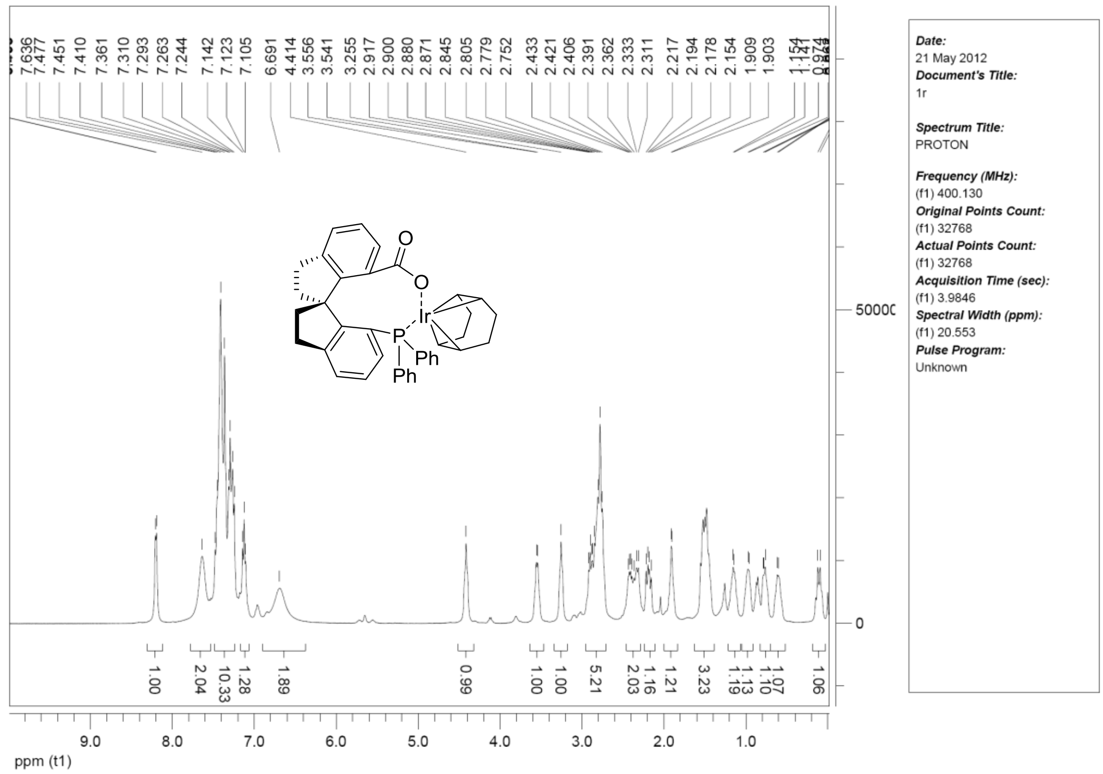
7.636
7.477
7.451
7.410
7.361
7.310
7.293
7.263
7.244
7.142
7.123
7.105
6.691
4.414
3.556
3.541
3.255
2.917
2.900
2.880
2.871
2.845
2.805
2.779
2.752
2.433
2.421
2.406
2.391
2.362
2.333
2.311
2.217
2.194
2.178
2.154
1.909
1.903
O
Ir
P
Ph
Ph
50000
0
1.00
2.04
10.33
1.28
1.89
0.99
1.00
1.00
5.21
2.03
1.16
1.21
3.23
1.19
1.13
1.10
1.07
1.06
9.0
8.0
7.0
6.0
5.0
4.0
3.0
2.0
1.0
ppm (t1)
Date:
21 May 2012
Document's Title:
1r
Spectrum Title:
PROTON
Frequency (MHz):
(f1) 400.130
Original Points Count:
(f1) 32768
Actual Points Count:
(f1) 32768
Acquisition Time (sec):
(f1) 3.9846
Spectral Width (ppm):
(f1) 20.553
Pulse Program:
Unknown

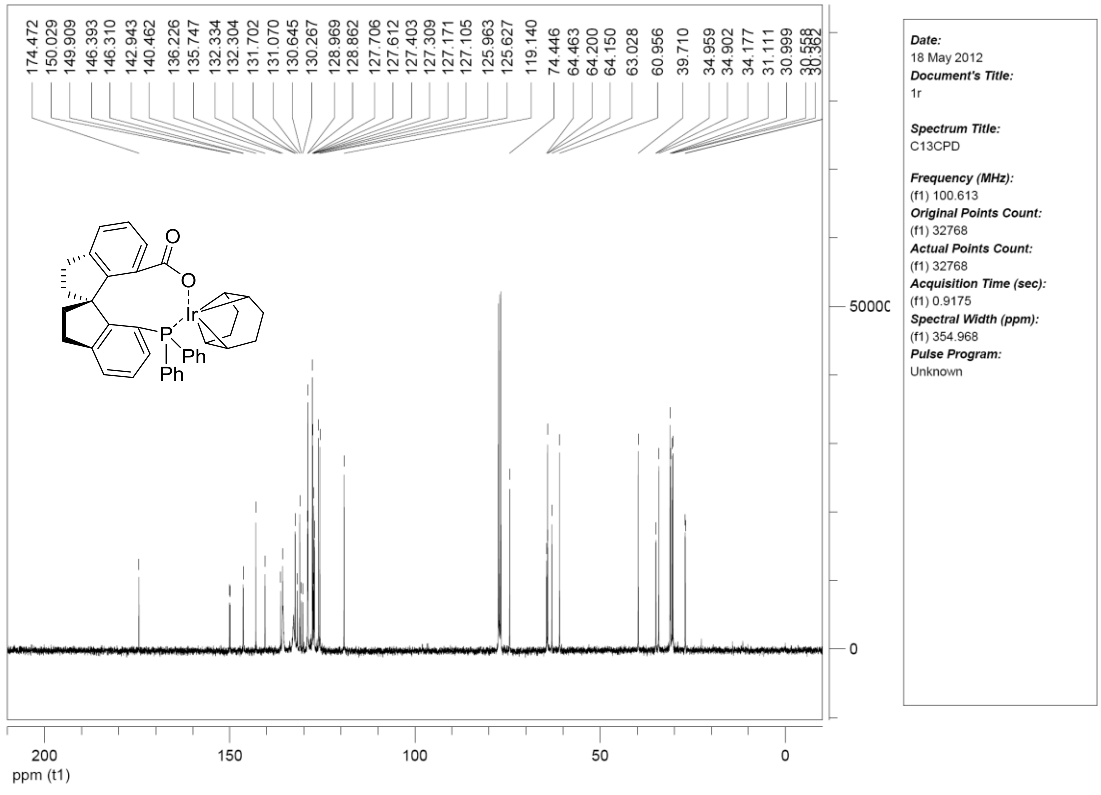
174.472
150.029
149.909
146.393
146.310
142.943
140.462
136.226
135.747
132.334
132.304
131.702
131.070
130.645
130.267
128.969
128.862
127.706
127.612
127.403
127.309
127.171
127.105
125.963
125.627
119.140
74.446
64.463
64.200
64.150
63.028
60.956
39.710
34.959
34.902
34.177
31.111
30.999
O
Ir
P
Ph
Ph
50000
0
200
150
100
50
0
ppm (t1)
Date:
18 May 2012
Document's Title:
1r
Spectrum Title:
C13CPD
Frequency (MHz):
(f1) 100.613
Original Points Count:
(f1) 32768
Actual Points Count:
(f1) 32768
Acquisition Time (sec):
(f1) 0.9175
Spectral Width (ppm):
(f1) 354.968
Pulse Program:
Unknown

3-Methylenedecanoic acid (5c)

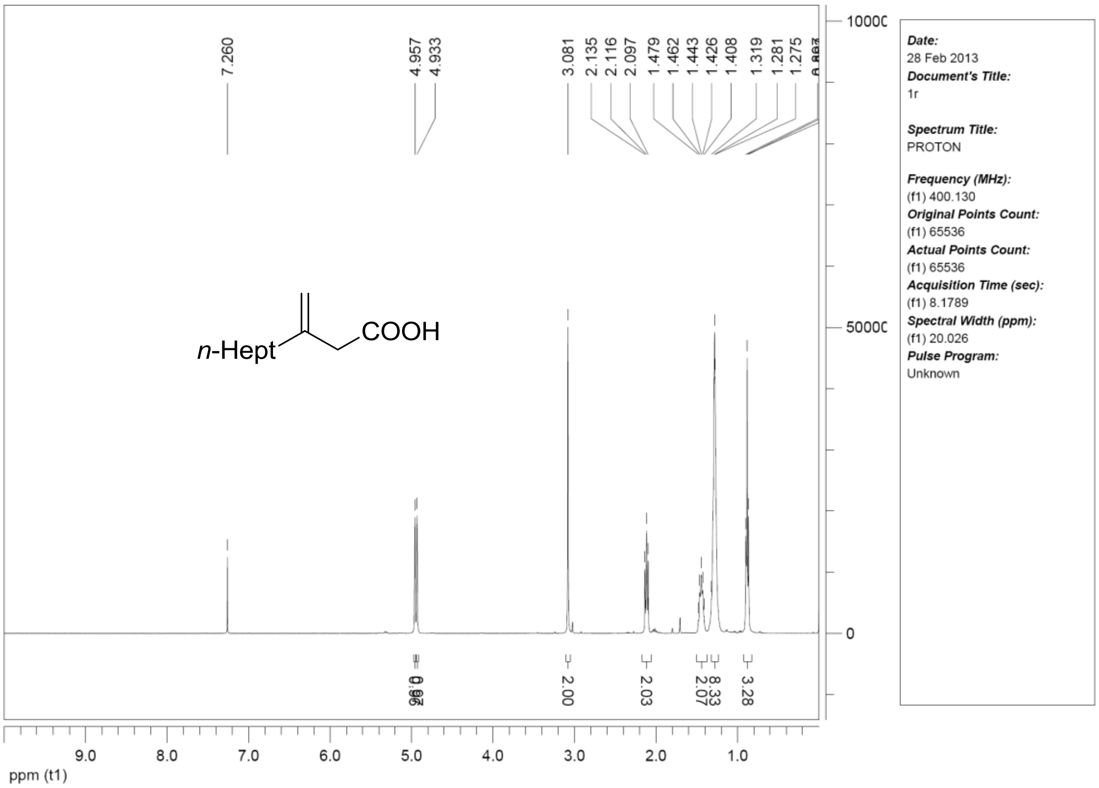

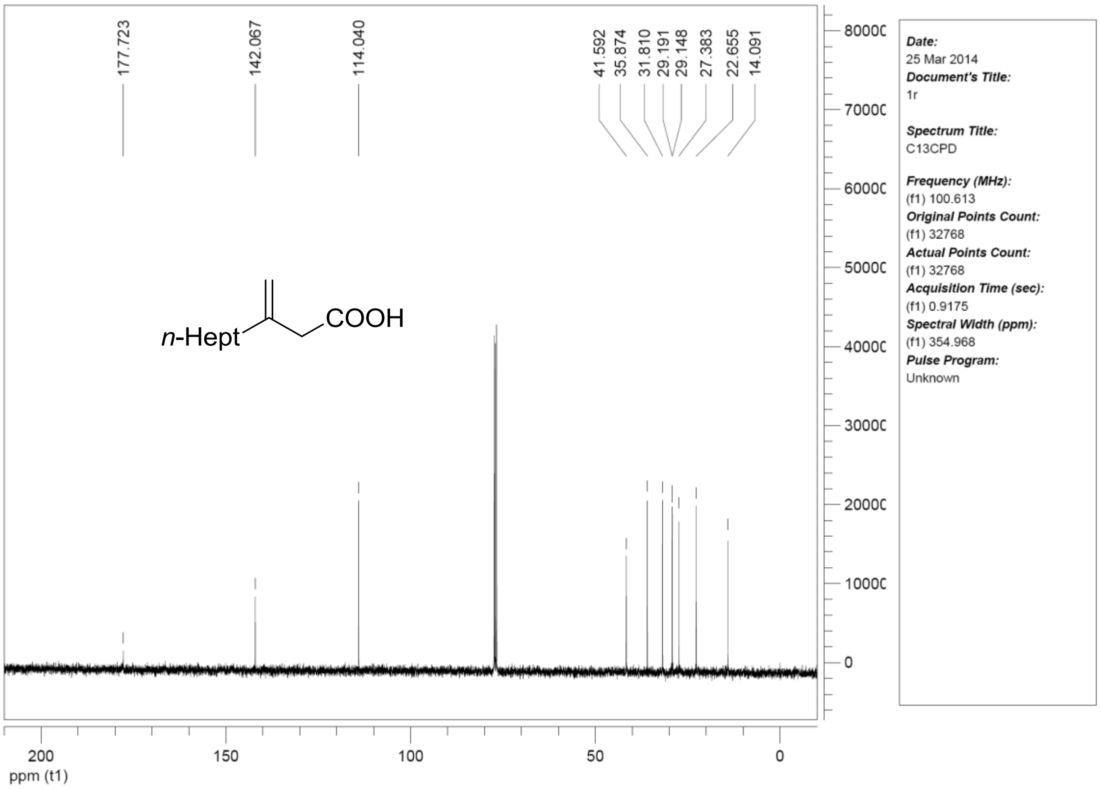

4-Methyl-3-methylenepentanoic acid (5d)

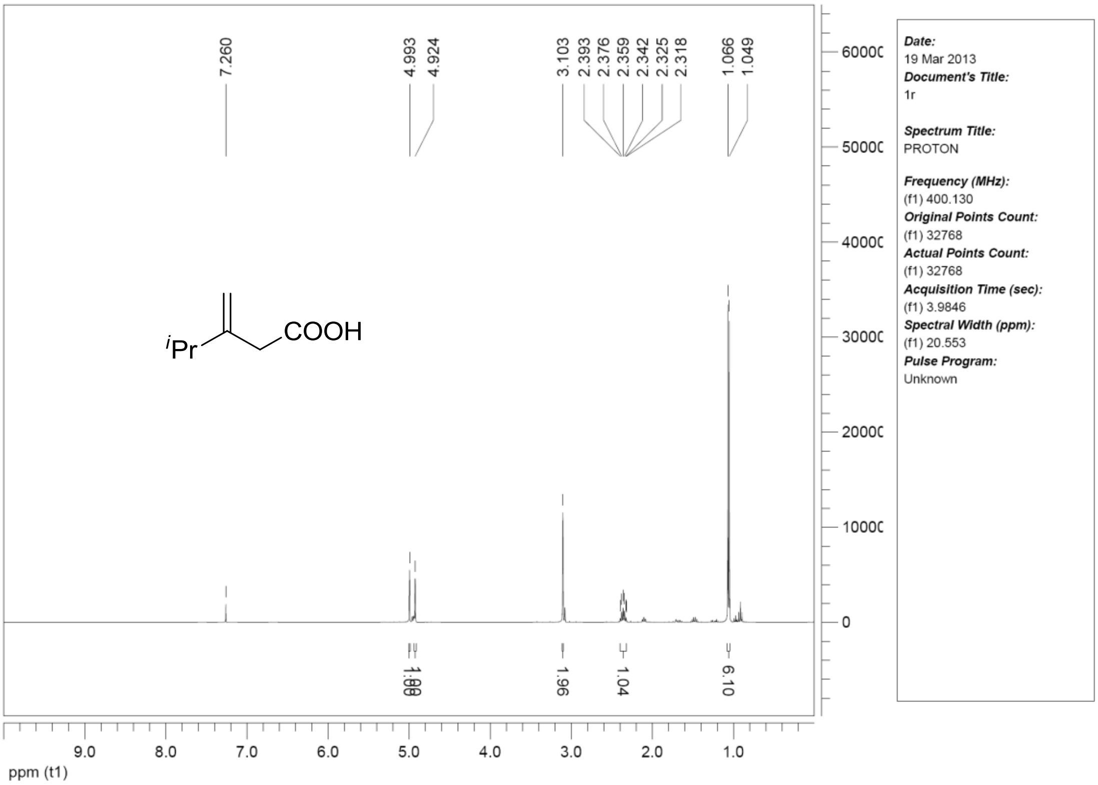

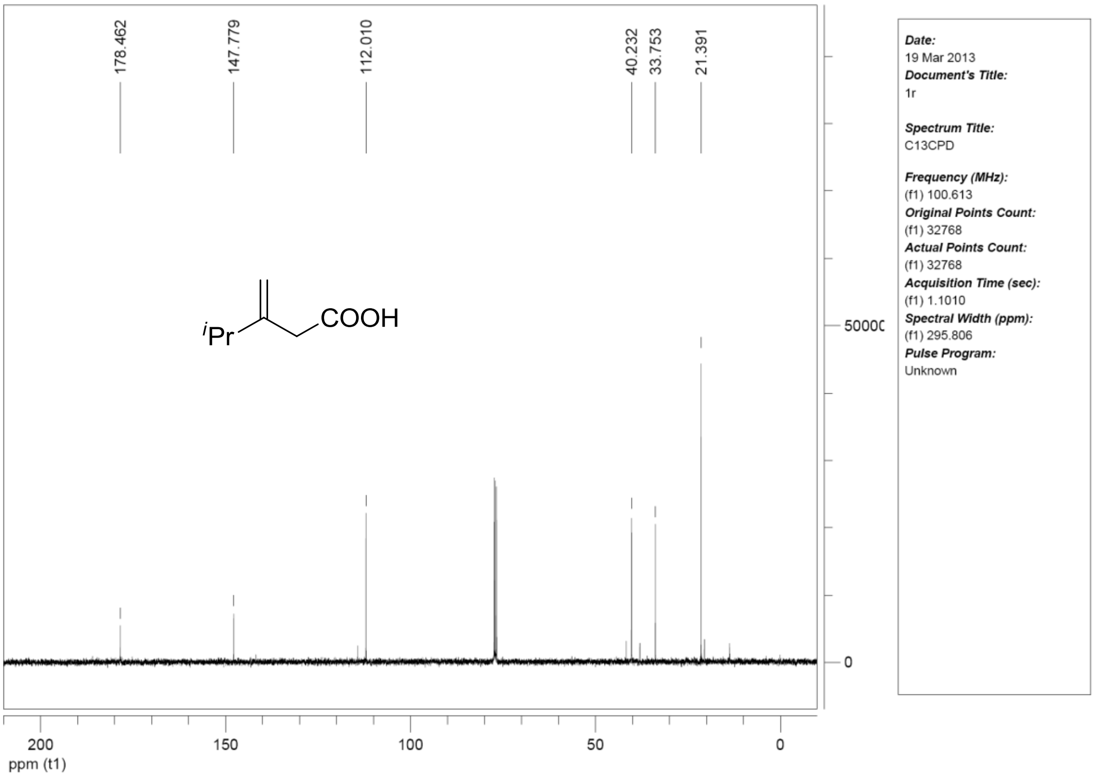

4-Ethyl-3-methylenehexanoic acid (5e)

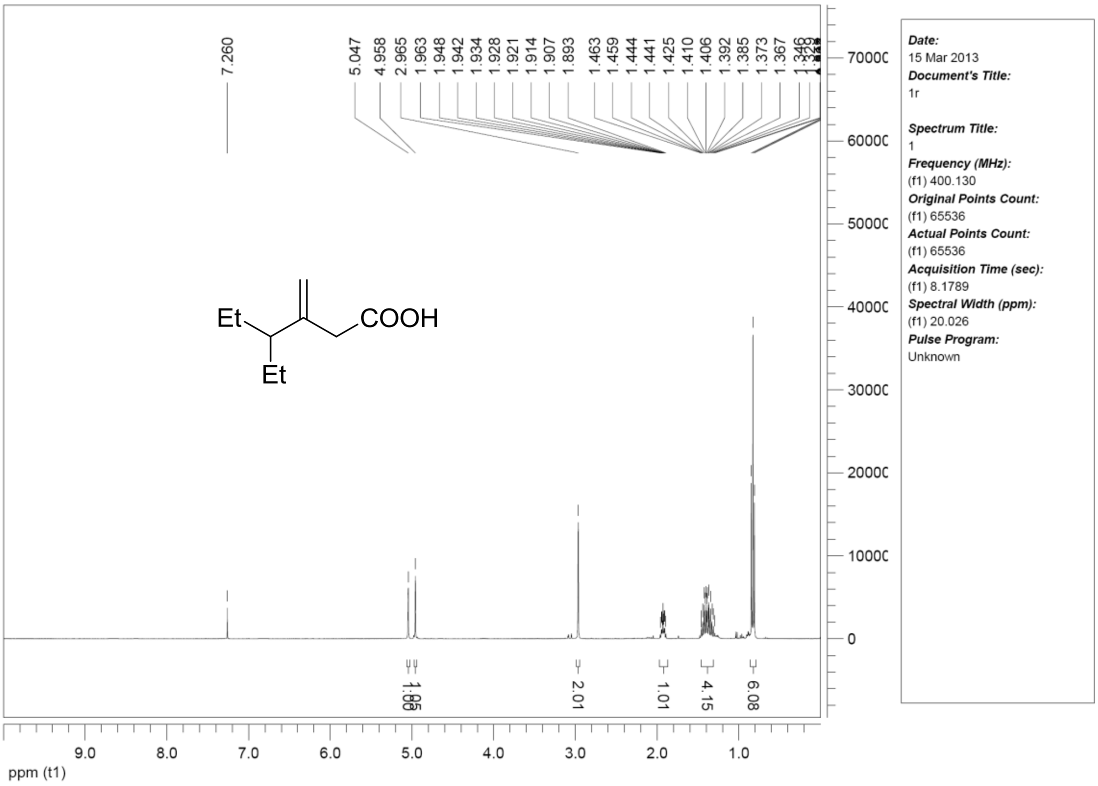

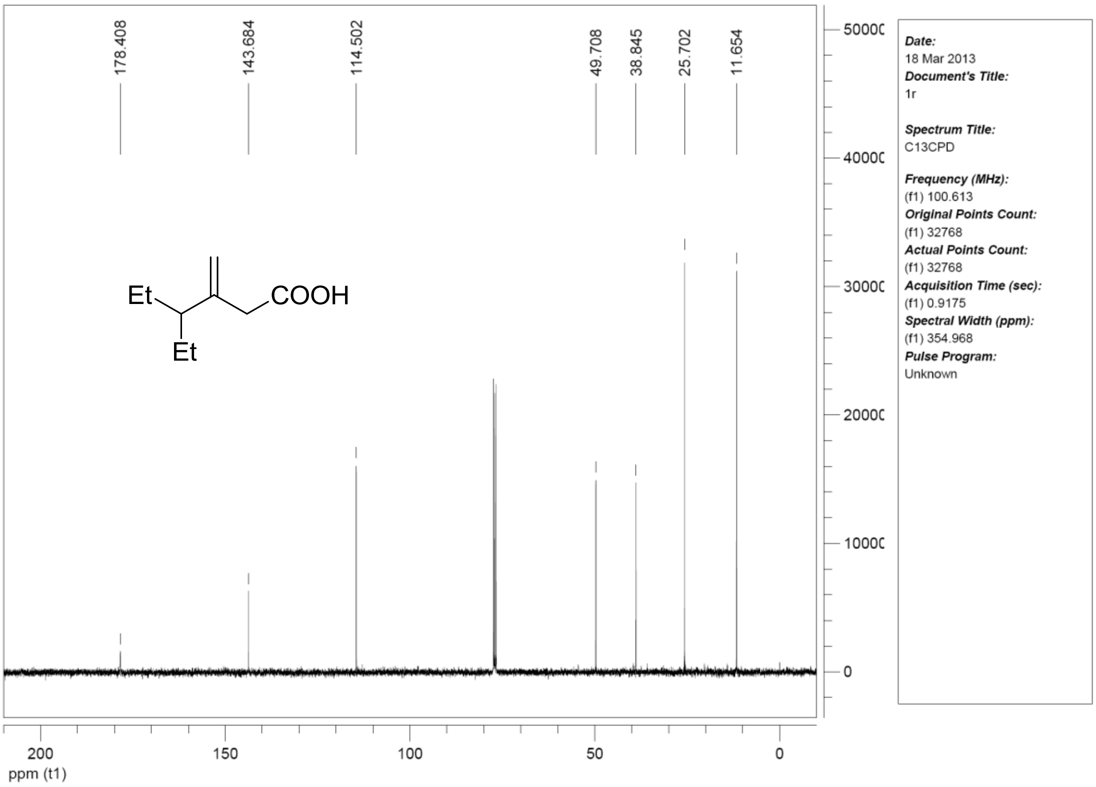

5-Methyl-3-methylenehexanoic acid (5f)

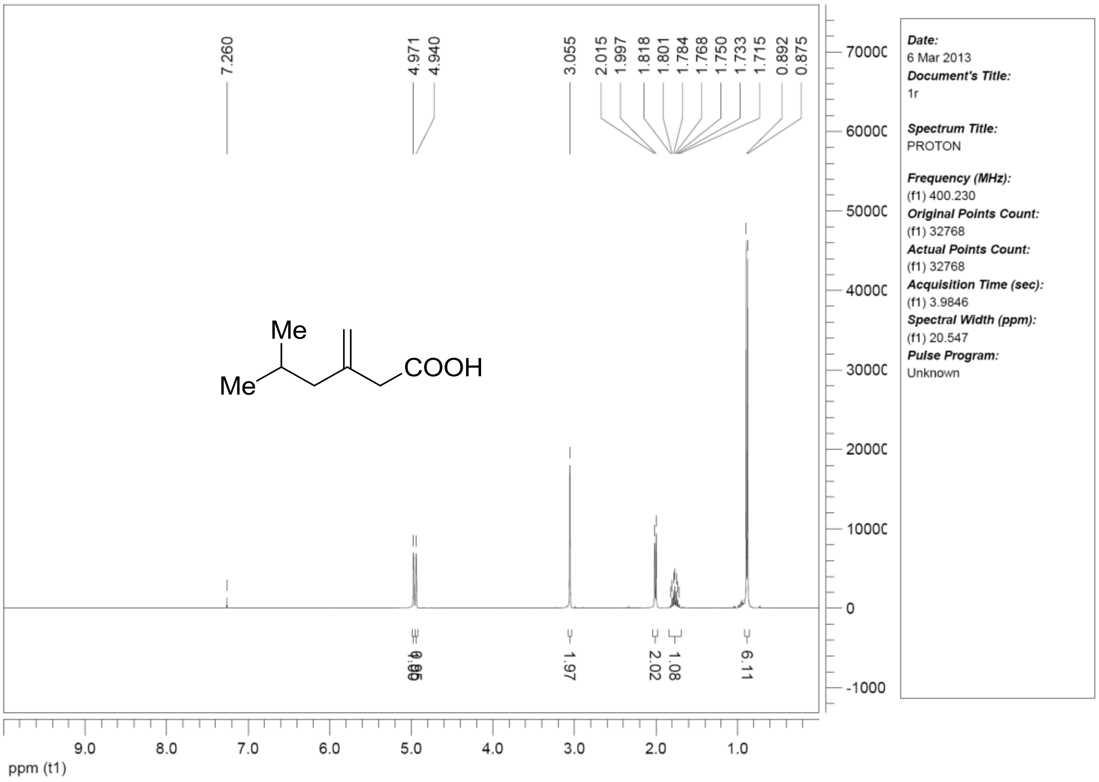

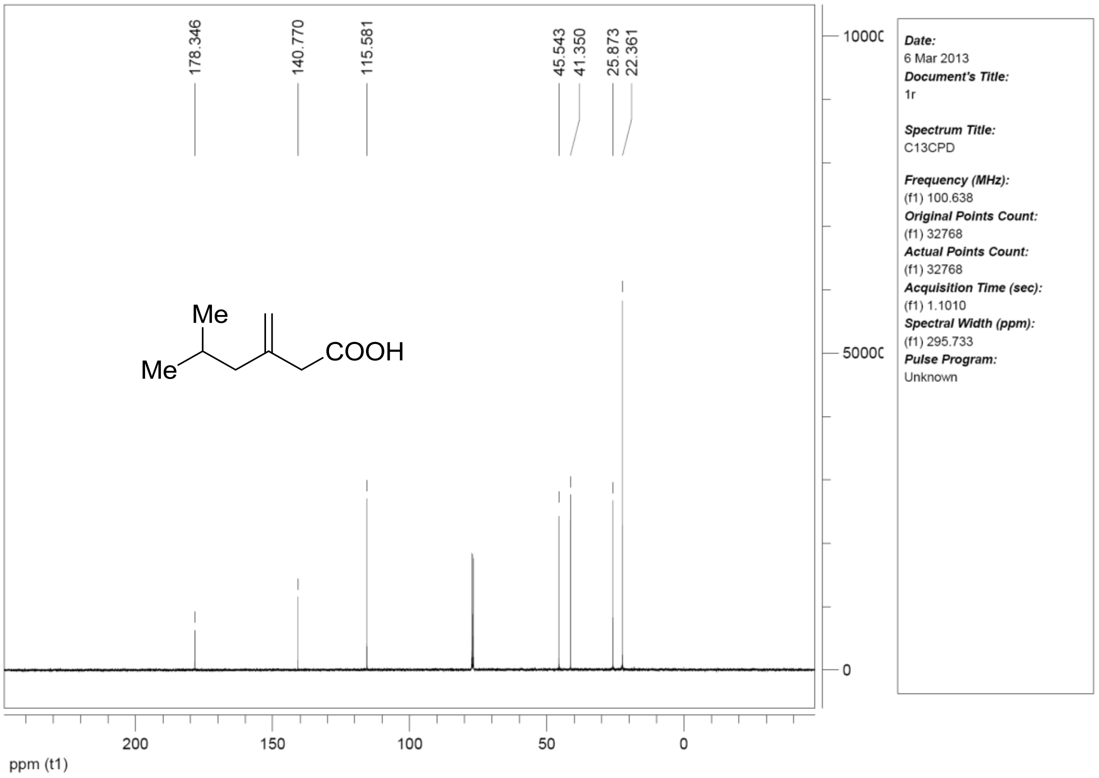

3-Cyclohexylbut-3-enoic acid (5g)

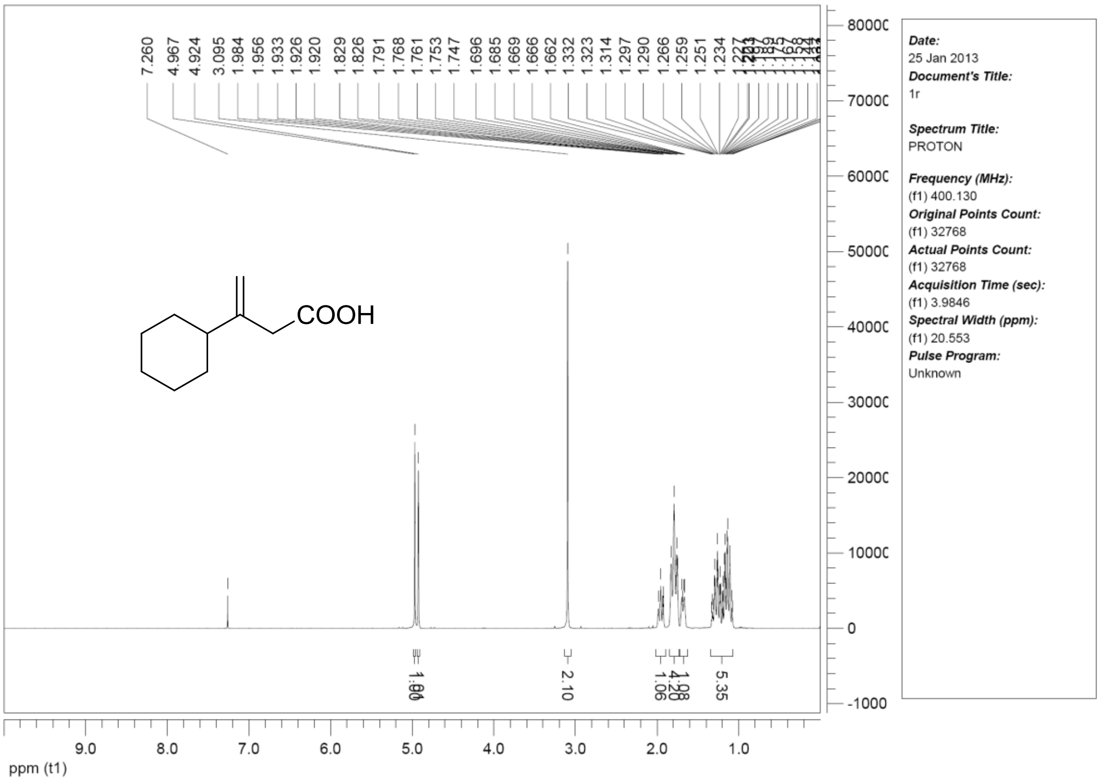

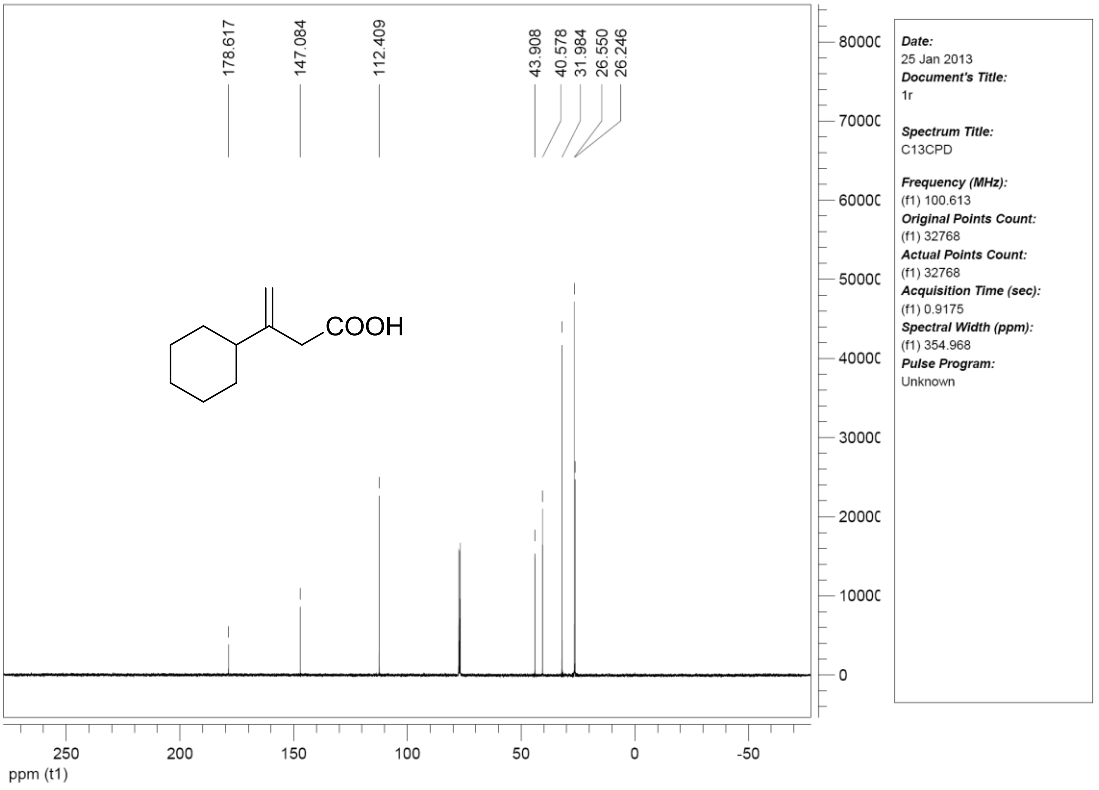

3-Methylene-5-phenylpentanoic acid (5h)

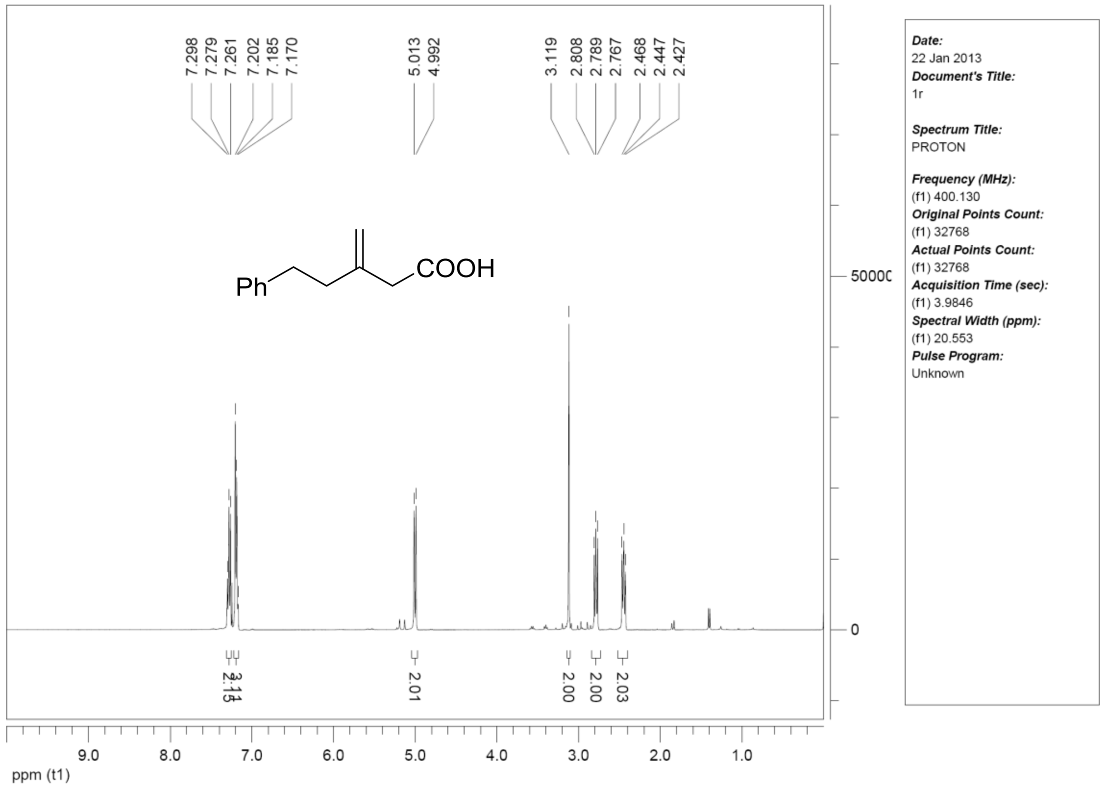

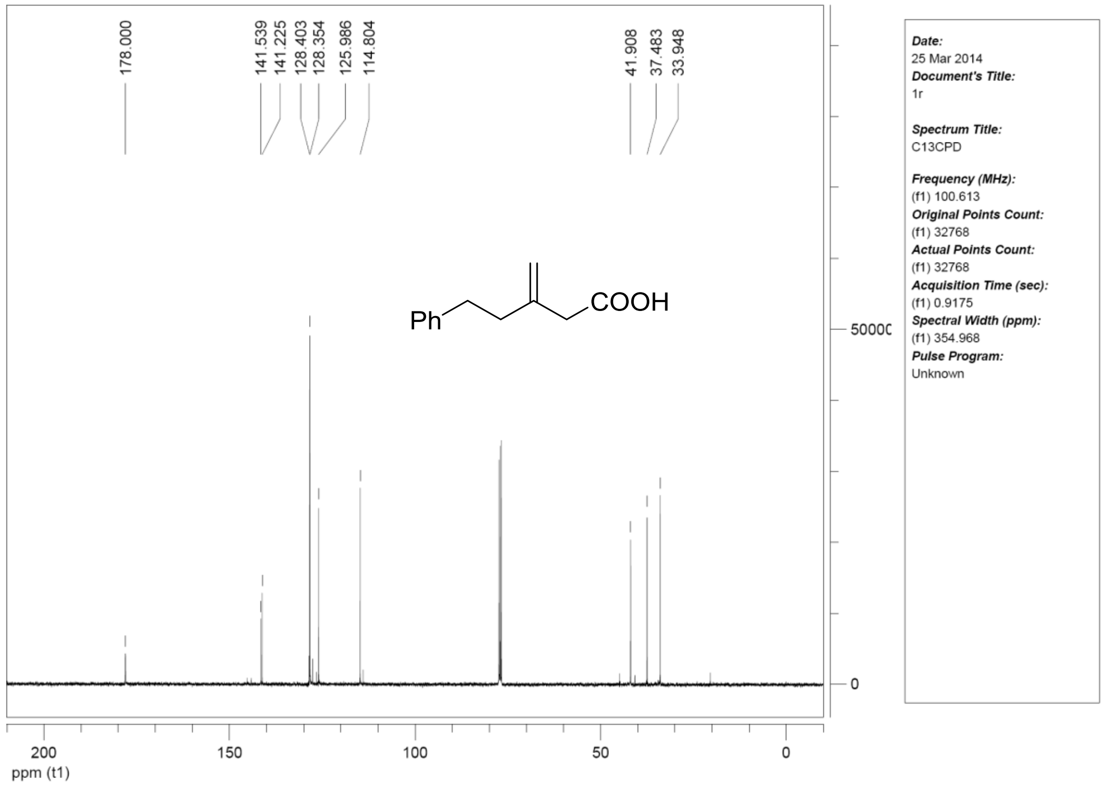

7-Methoxy-3-methyleneheptanoic acid (5i)

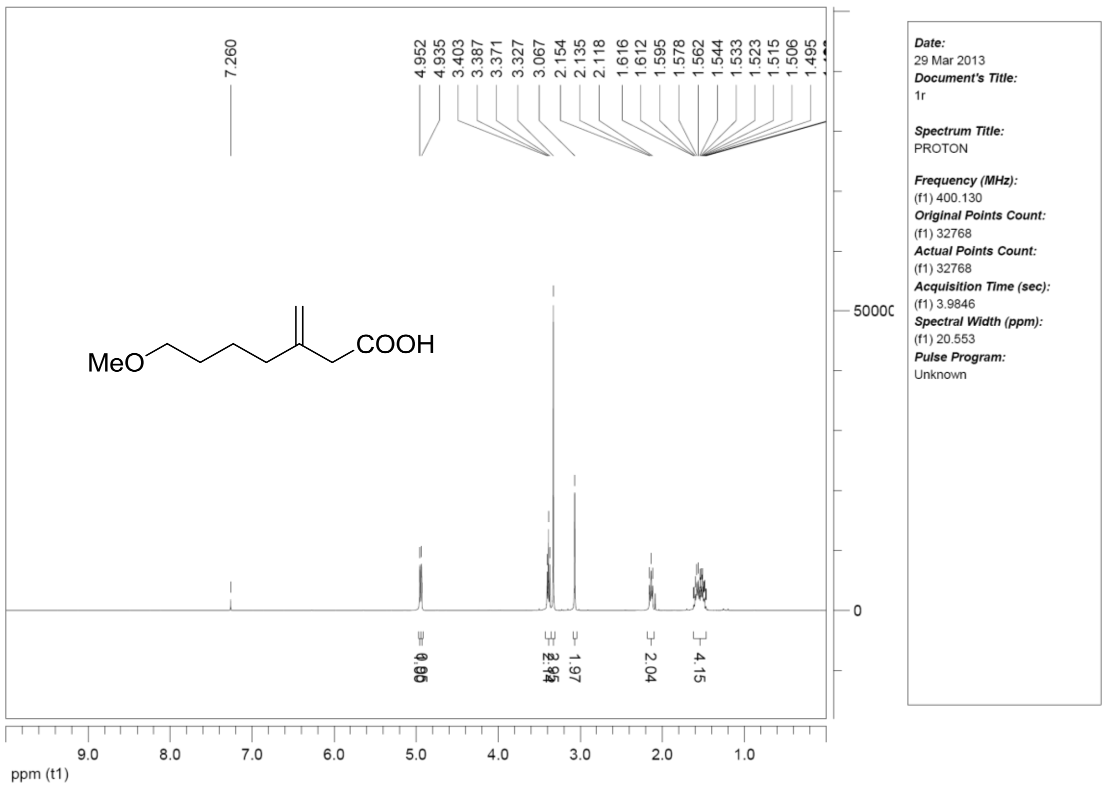

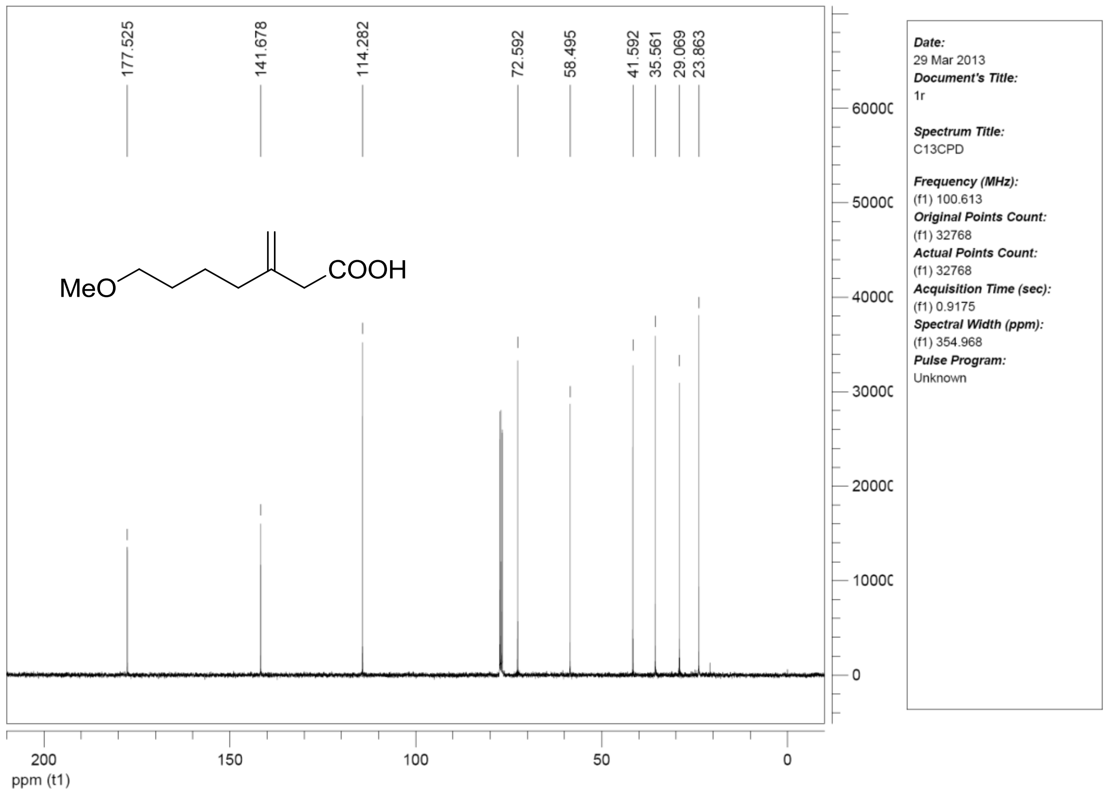

7-Methyl-3-methyleneoct-6-enoic acid (5j)

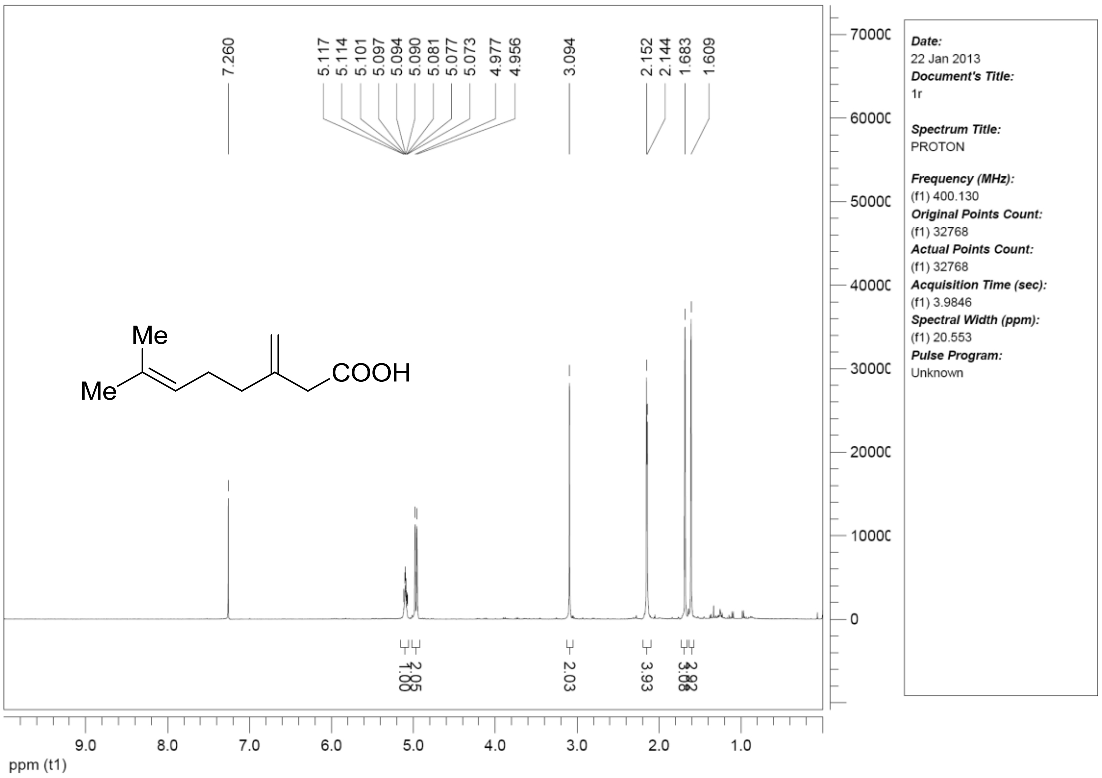

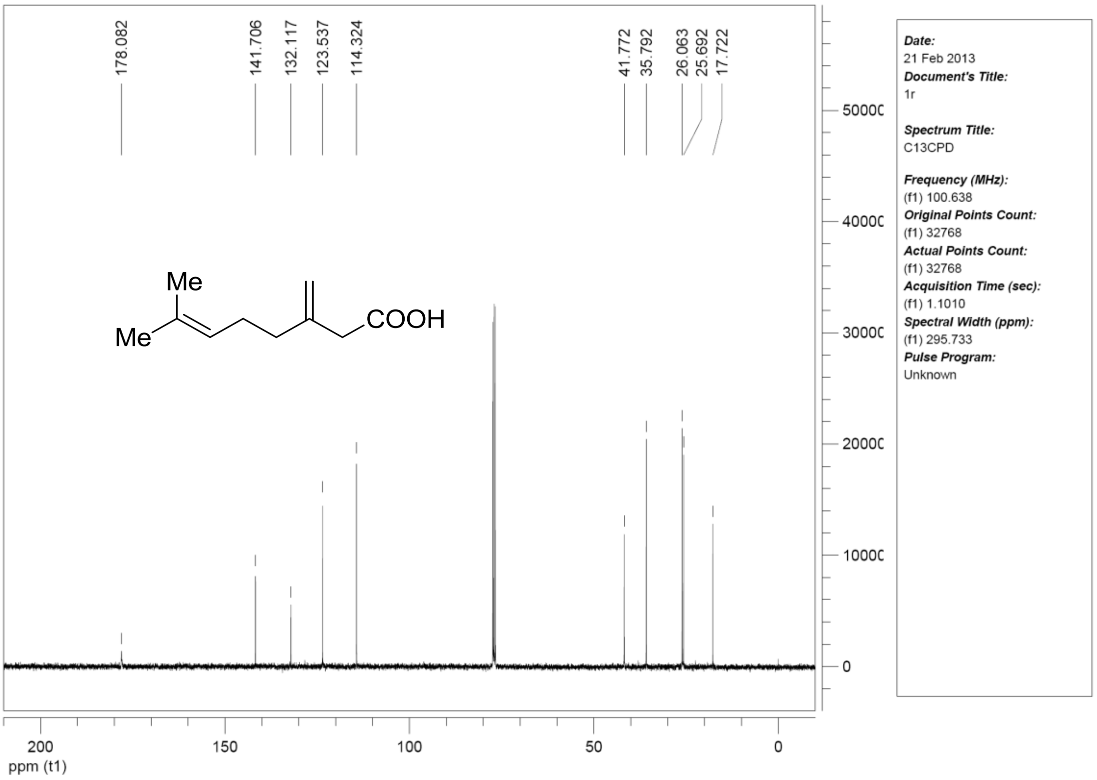

3-Methyldecanoic acid (6c)

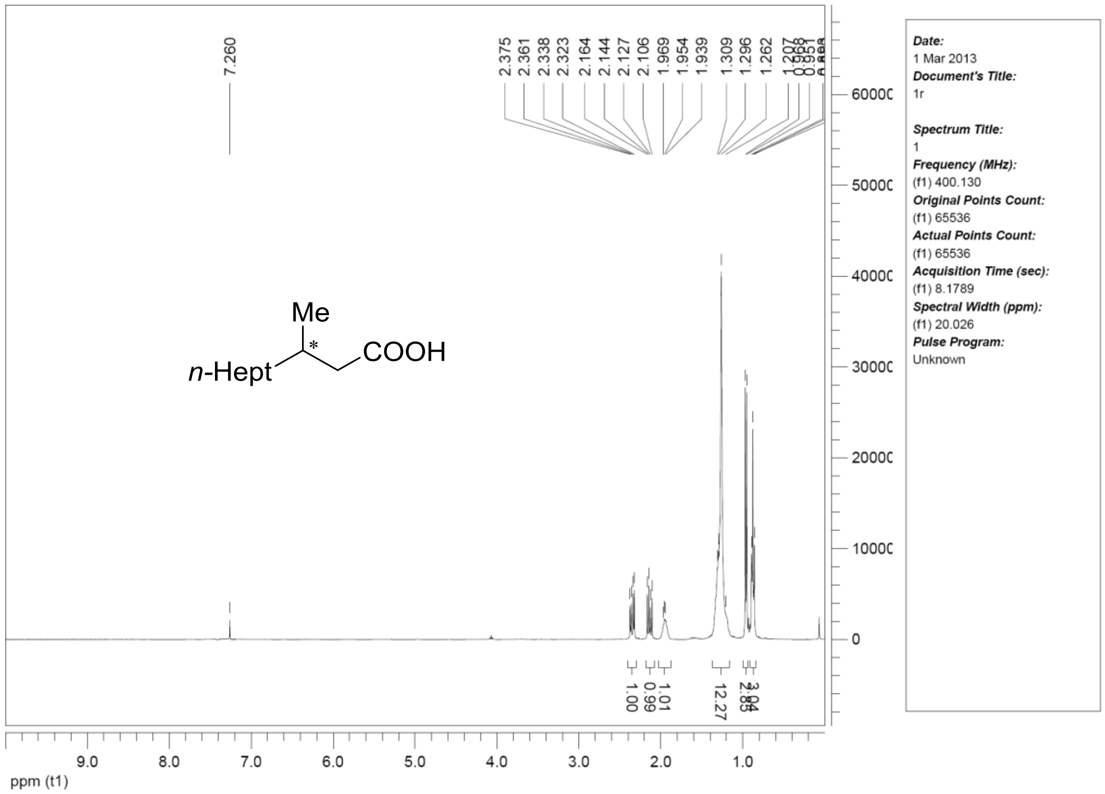

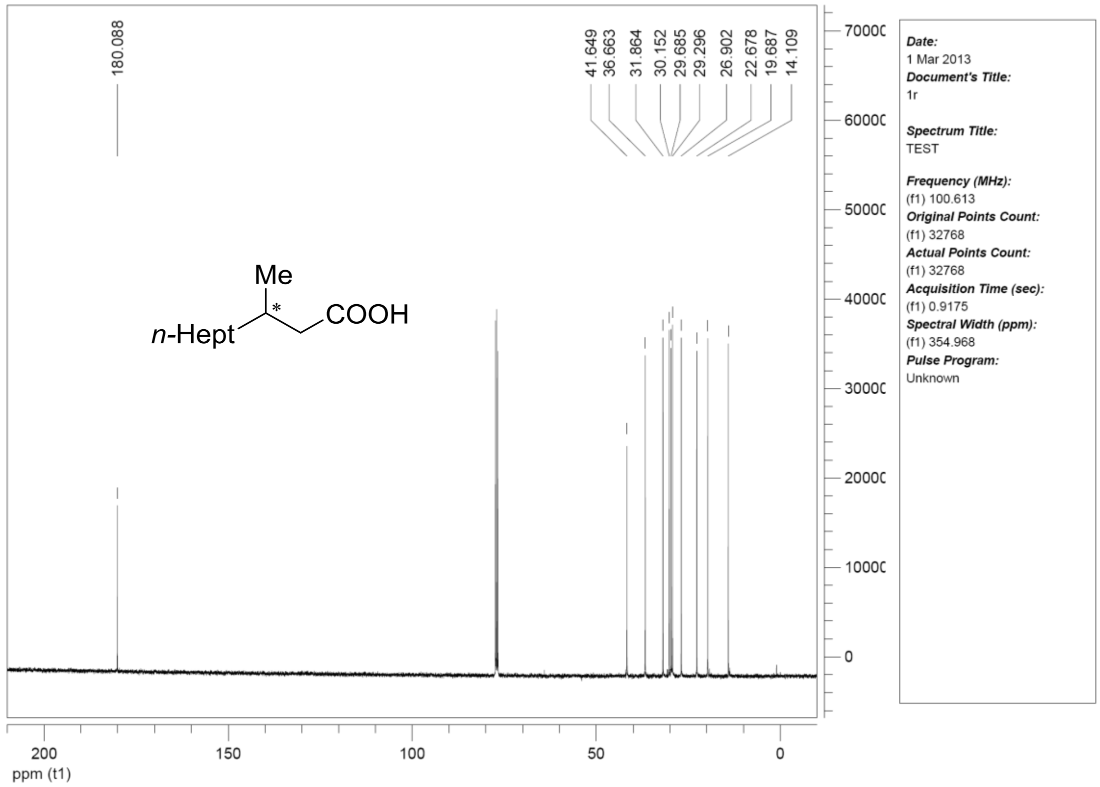

4-Ethyl-3-methylhexanoic acid (6e)

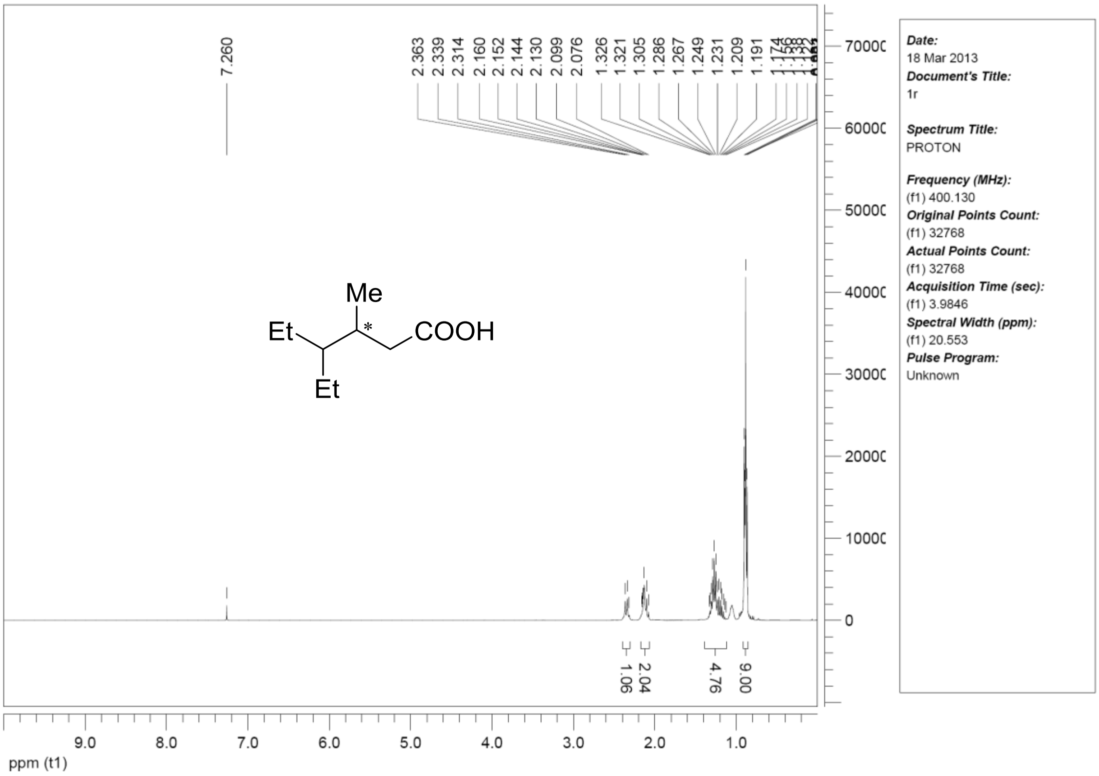

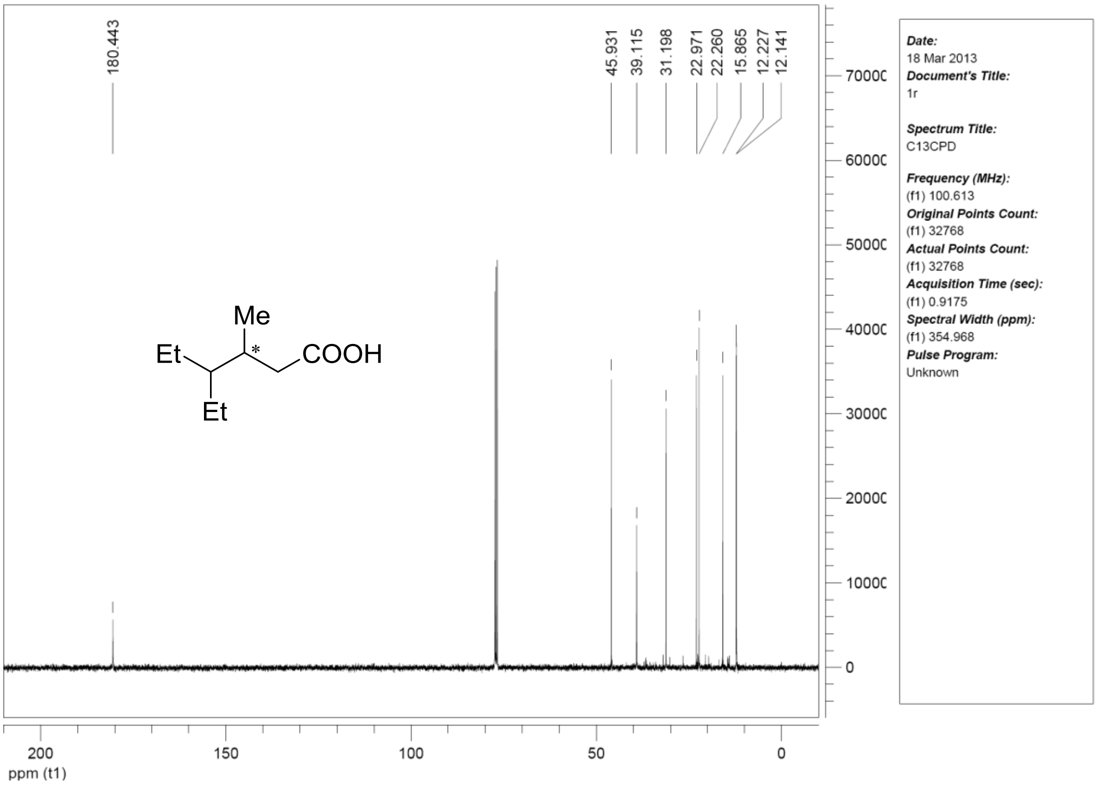

(*R*)-3,5-Dimethylhexanoic acid (6f)

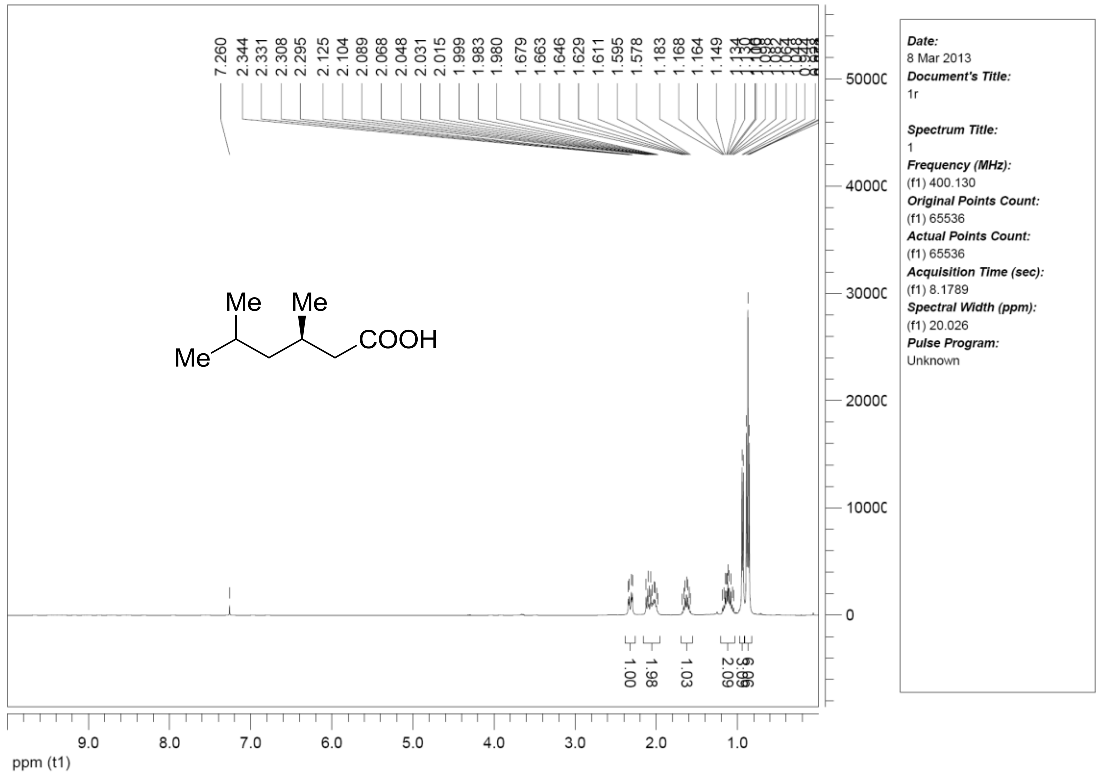

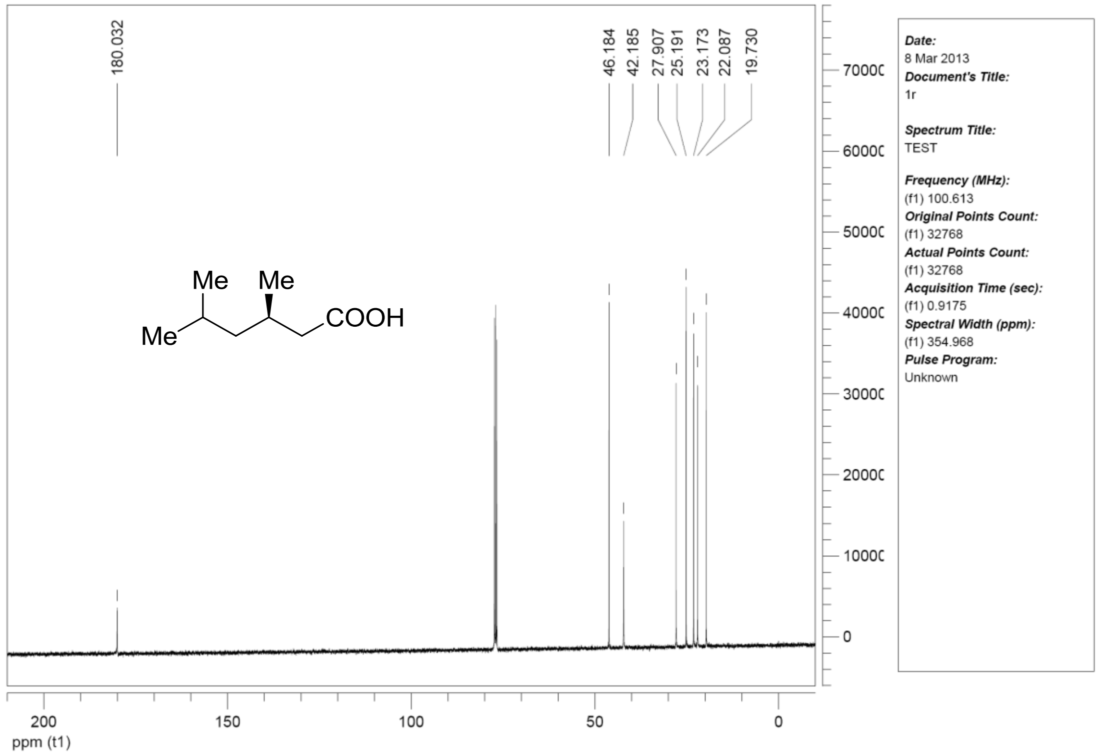

7-Methoxy-3-methylheptanoic acid (6i)

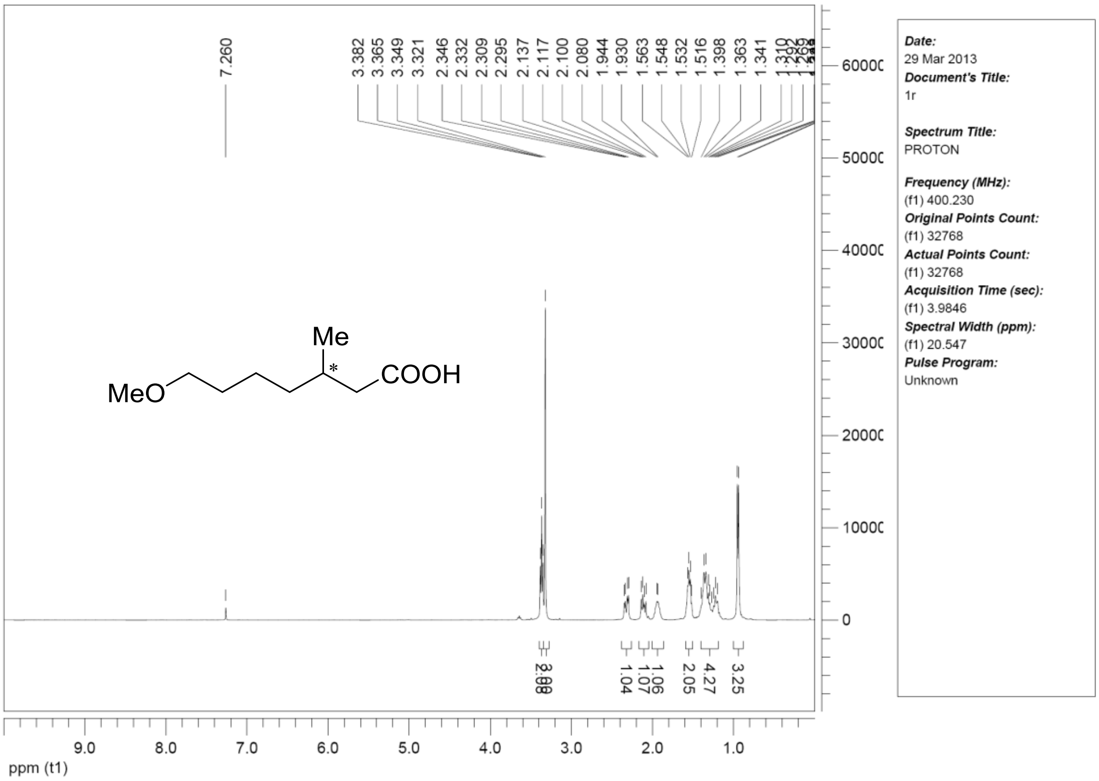

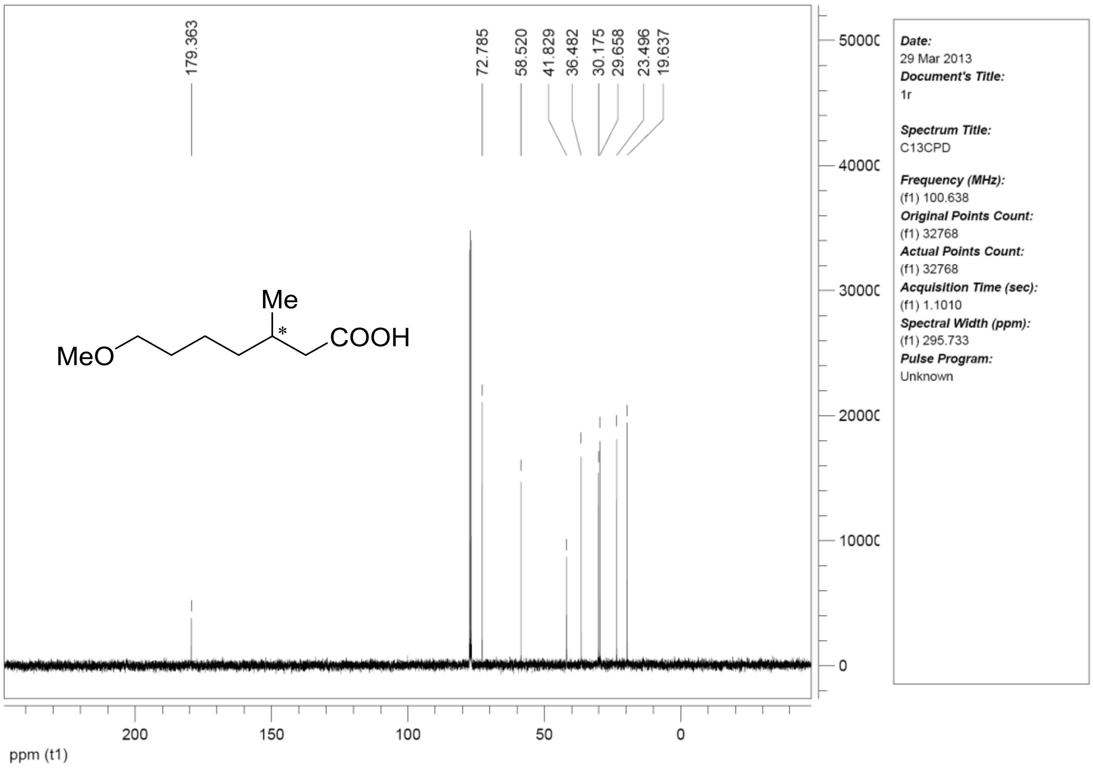

7. HPLC or GC Charts of Hydrogenation Product Derivatives

(*R*)-3-Methyl-N-phenylheptanamide (6a)

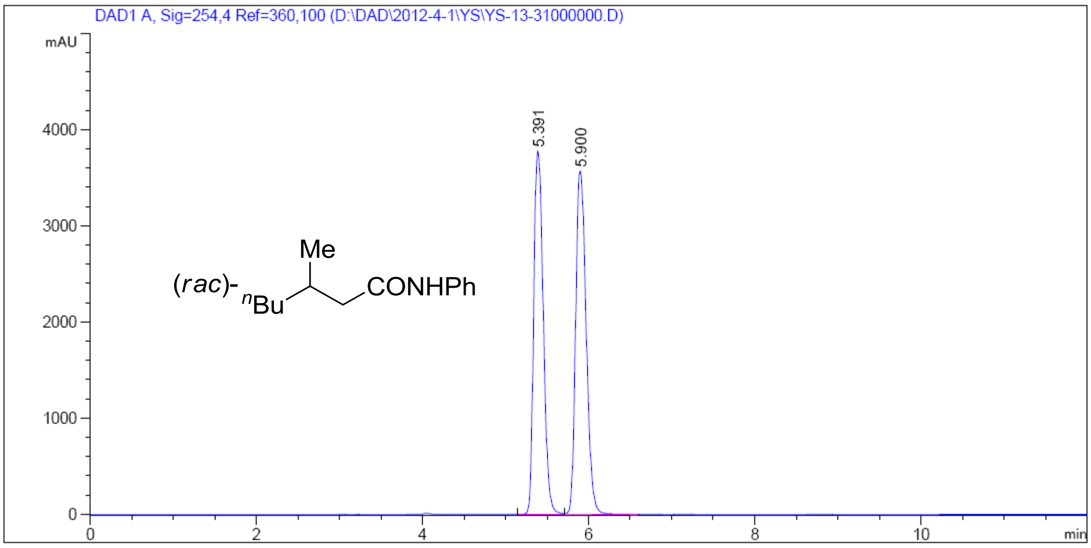

Peak #	RetTime [min]	Type	Width [min]	Area [mAU*s]	Height [mAU]	Area %
1	5.391	BV	0.1292	3.00976e4	3766.09644	48.9770
2	5.900	VV	0.1386	3.13549e4	3565.60352	51.0230

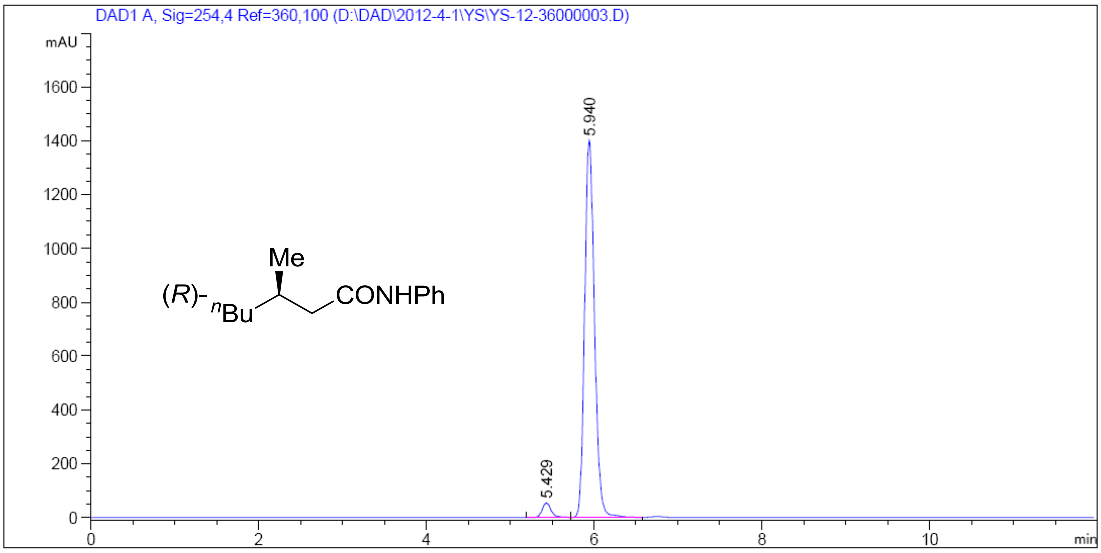

Peak #	RetTime [min]	Type	Width [min]	Area [mAU*s]	Height [mAU]	Area %
1	5.429	BV	0.1149	400.56201	53.77439	3.3813
2	5.940	VV	0.1274	1.14458e4	1399.86182	96.6187

(*R*)-3-Methyl-N-phenylpentanamide (6b)

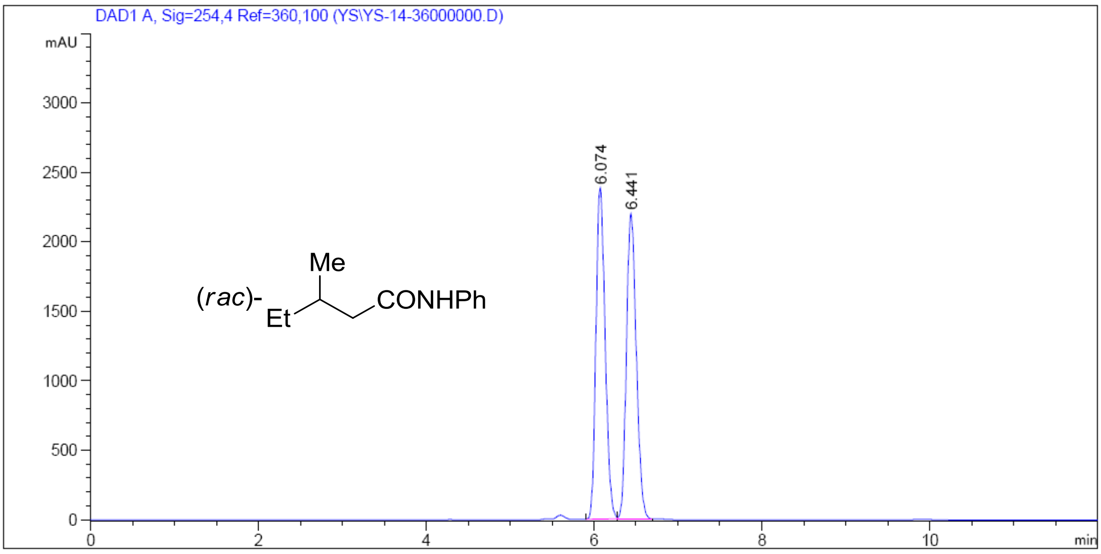

Peak #	RetTime [min]	Type	Width [min]	Area [mAU*s]	Height [mAU]	Area %
1	6.074	BV	0.1211	1.81818e4	2381.79077	50.2973
2	6.441	VB	0.1276	1.79668e4	2192.70605	49.7027

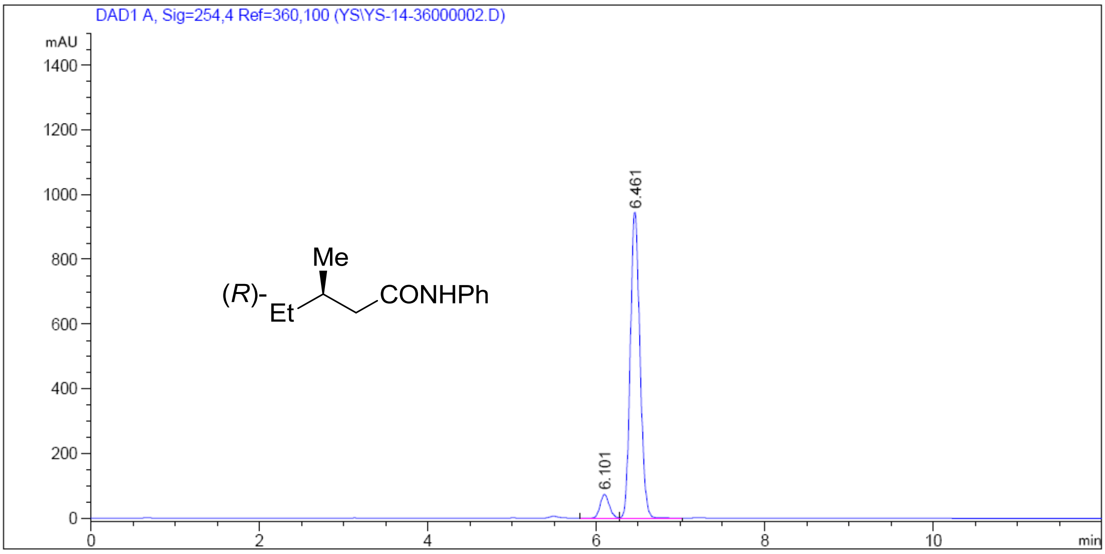

Peak #	RetTime [min]	Type	Width [min]	Area [mAU*s]	Height [mAU]	Area %
1	6.101	BV	0.1160	551.33026	73.09827	6.8041
2	6.461	VB	0.1252	7551.61914	944.85028	93.1959

3-Methyl-N-phenyldecanamide (6c)

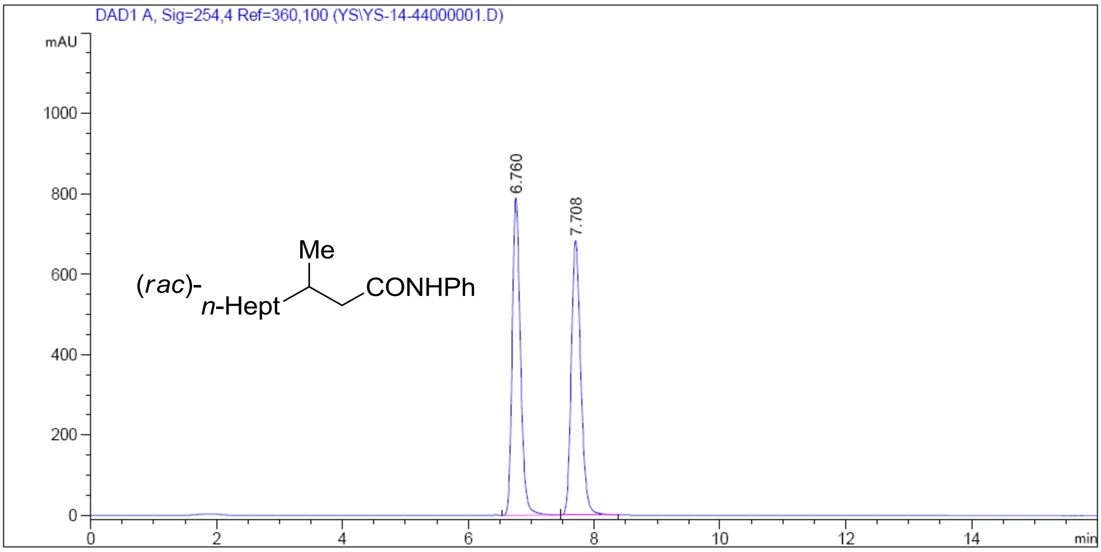

Peak #	RetTime [min]	Type	Width [min]	Area [mAU*s]	Height [mAU]	Area %
1	6.760	VB	0.1399	7169.24658	789.90051	50.0641
2	7.708	BB	0.1624	7150.89502	681.82556	49.9359

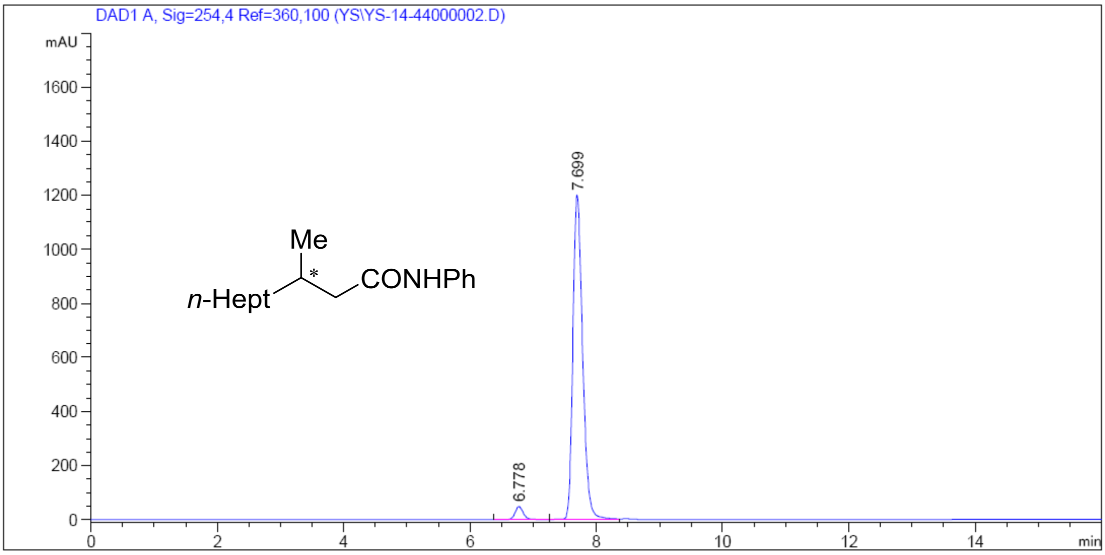

Peak #	RetTime [min]	Type	Width [min]	Area [mAU*s]	Height [mAU]	Area %
1	6.778	BB	0.1379	427.37921	47.98709	3.2225
2	7.699	BV	0.1648	1.28350e4	1200.26758	96.7775

(*S*)-3,4-Dimethyl-N-phenylpentanamide (6d)

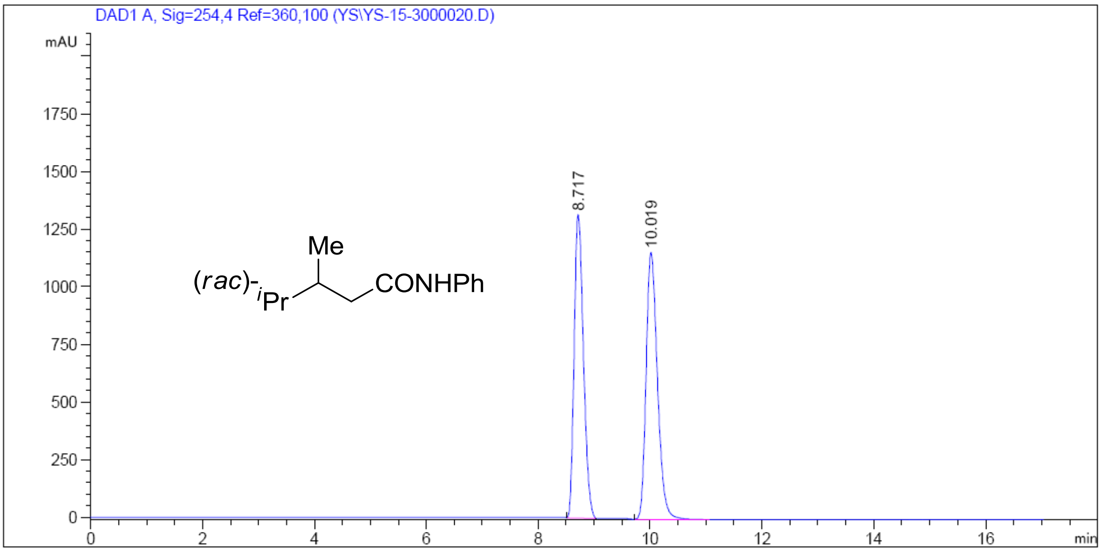

Peak #	RetTime [min]	Type	Width [min]	Area [mAU*s]	Height [mAU]	Area %
1	8.717	BB	0.1805	1.51780e4	1316.33972	48.2807
2	10.019	BB	0.2179	1.62590e4	1157.25415	51.7193

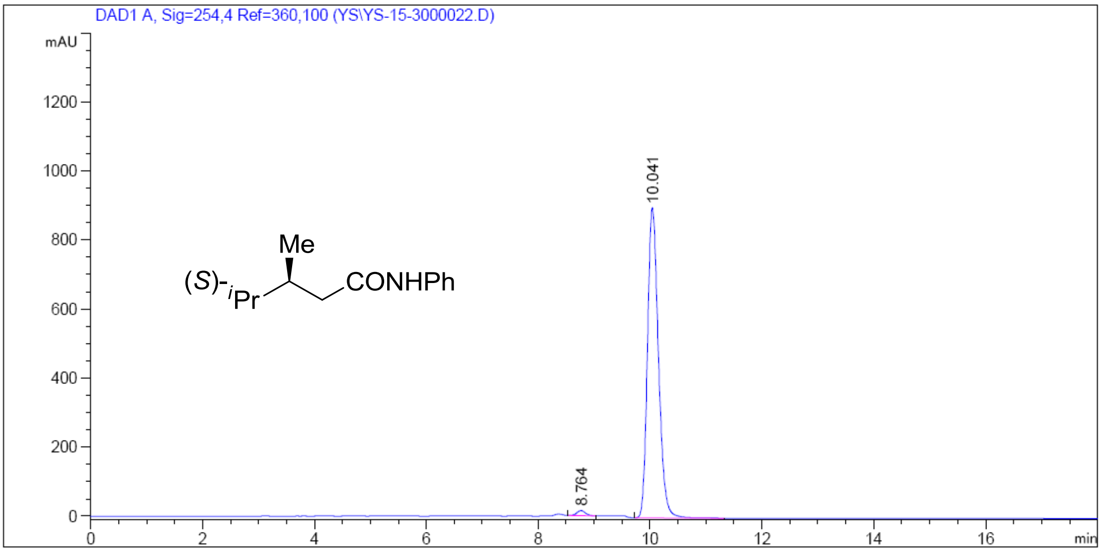

Peak #	RetTime [min]	Type	Width [min]	Area [mAU*s]	Height [mAU]	Area %
1	8.764	BB	0.1676	157.00070	14.58230	1.2735
2	10.041	BB	0.2100	1.21710e4	898.67847	98.7265

4-Ethyl-3-methyl-N-phenylhexanamide (6e)

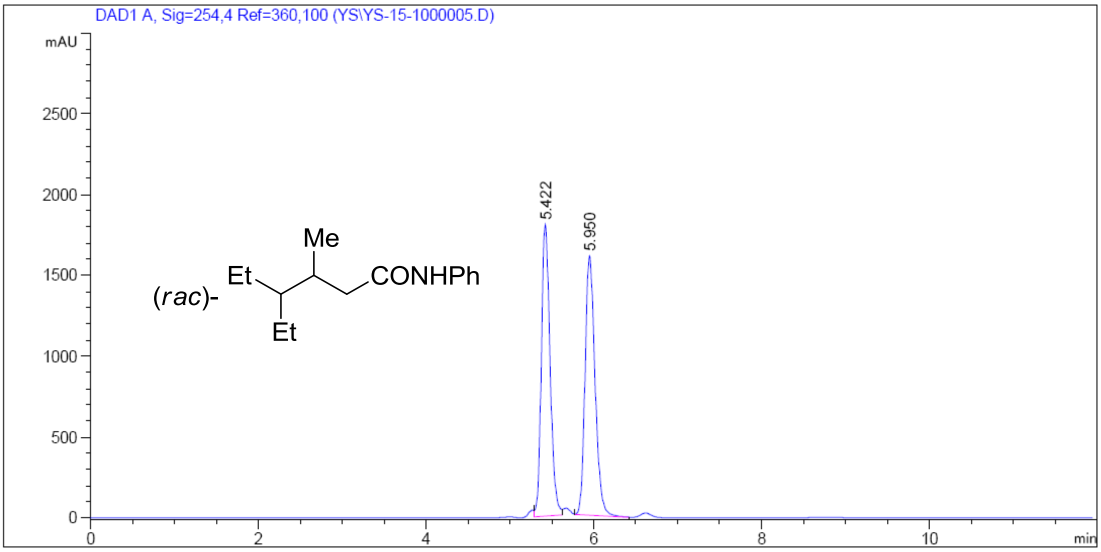

Peak #	RetTime [min]	Type	Width [min]	Area [mAU*s]	Height [mAU]	Area %
1	5.422	VV	0.1103	1.30356e4	1803.39868	49.5895
2	5.950	BB	0.1284	1.32514e4	1604.11804	50.4105

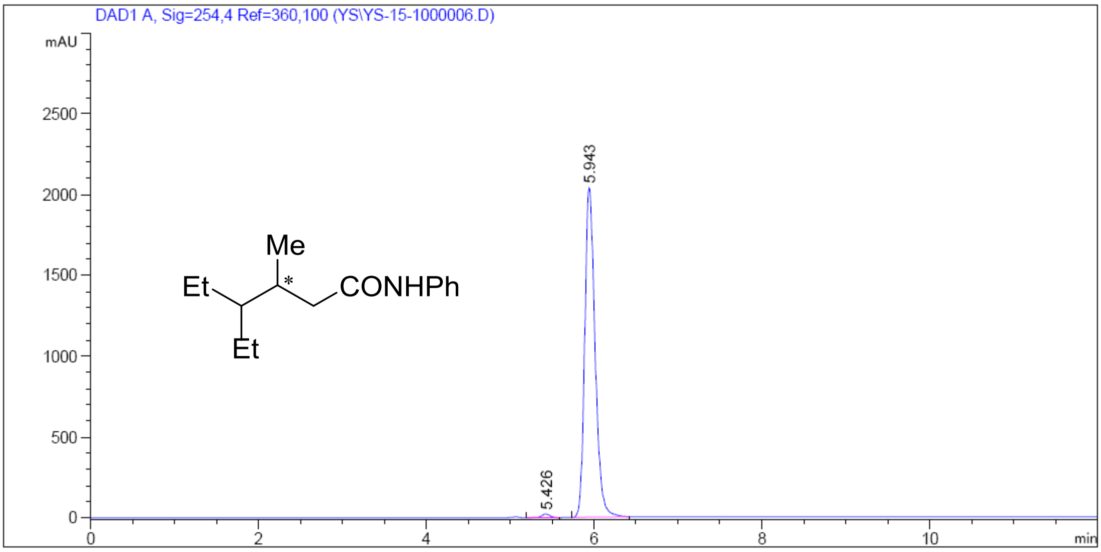

Peak #	RetTime [min]	Type	Width [min]	Area [mAU*s]	Height [mAU]	Area %
1	5.426	BB	0.1094	162.20784	22.68942	0.9221
2	5.943	BB	0.1317	1.74287e4	2039.17822	99.0779

(*R*)-3,5-Dimethyl-N-phenylhexanamide (6f)

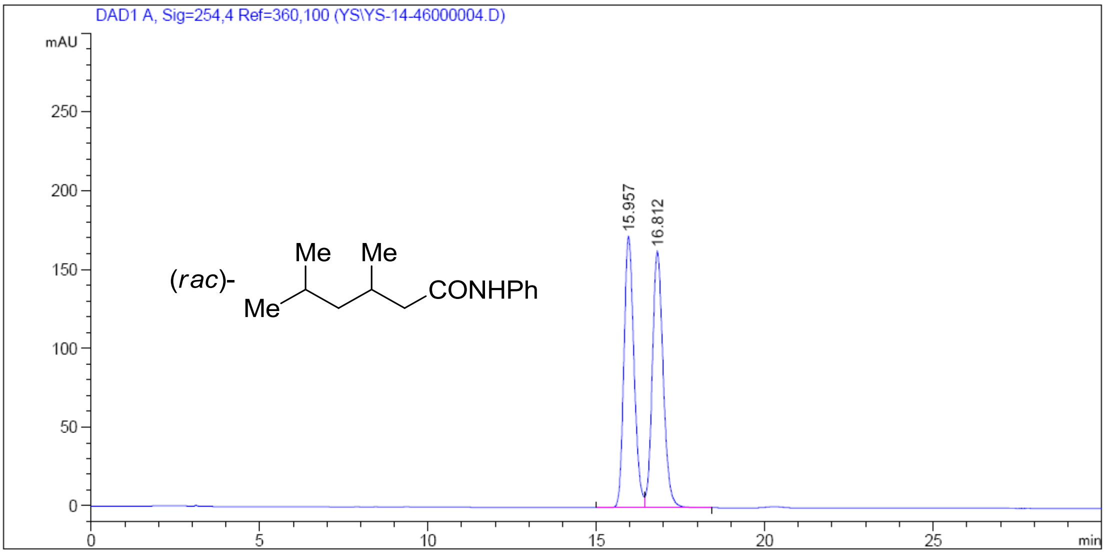

Peak #	RetTime [min]	Type	Width [min]	Area [mAU*s]	Height [mAU]	Area %
1	15.957	BV	0.3202	3574.43140	172.19176	49.4526
2	16.812	VB	0.3438	3653.56348	162.83850	50.5474

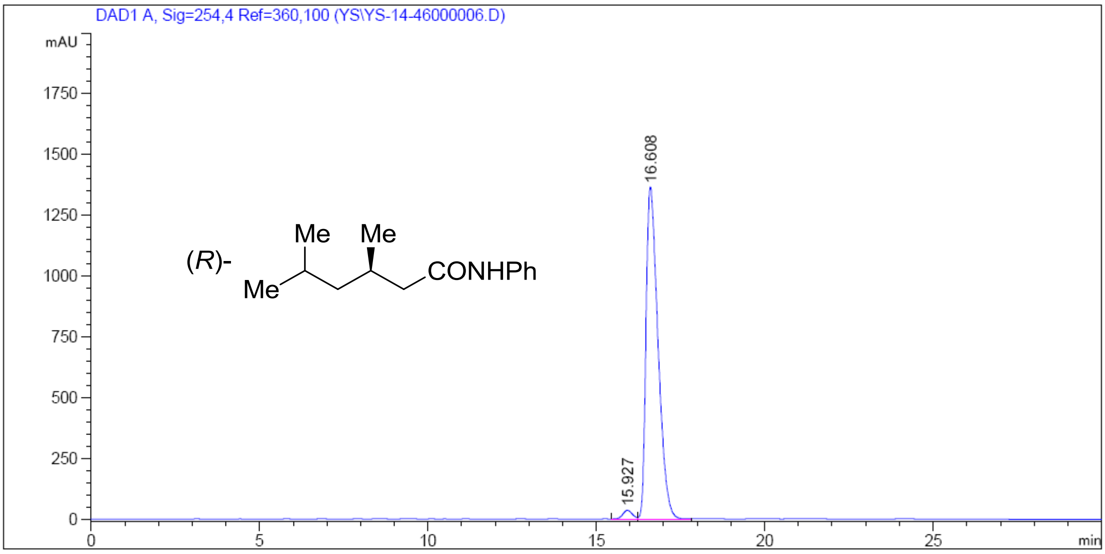

Peak #	RetTime [min]	Type	Width [min]	Area [mAU*s]	Height [mAU]	Area %
1	15.927	VV	0.3037	696.61780	35.69996	2.0161
2	16.608	VV	0.3814	3.38559e4	1364.76086	97.9839

3-Cyclohexyl-N-phenylbutanamide (6g)

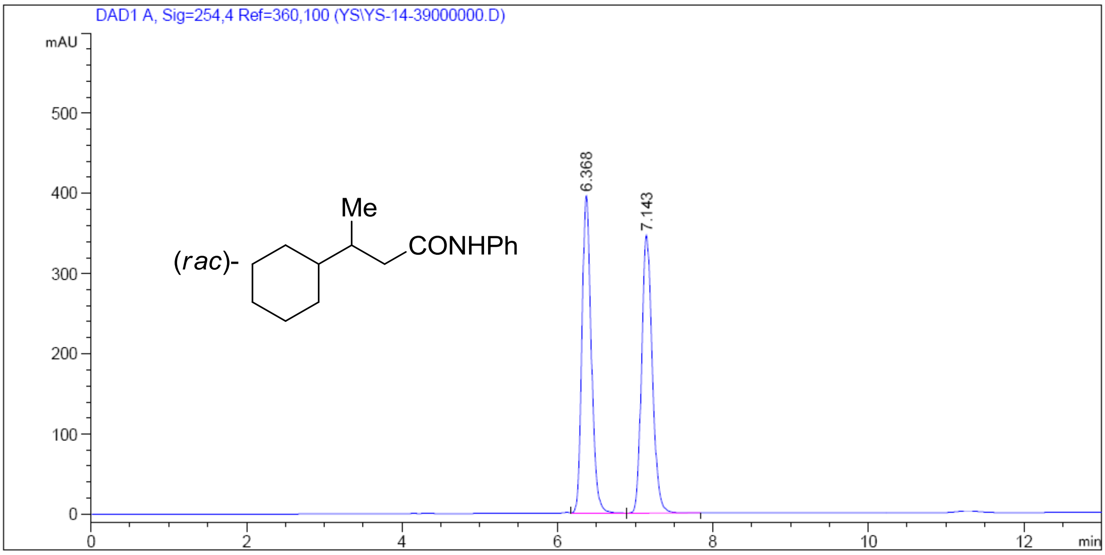

Peak #	RetTime [min]	Type	Width [min]	Area [mAU*s]	Height [mAU]	Area %
1	6.368	VB	0.1298	3315.75781	395.58499	50.0301
2	7.143	BB	0.1477	3311.77026	345.76700	49.9699

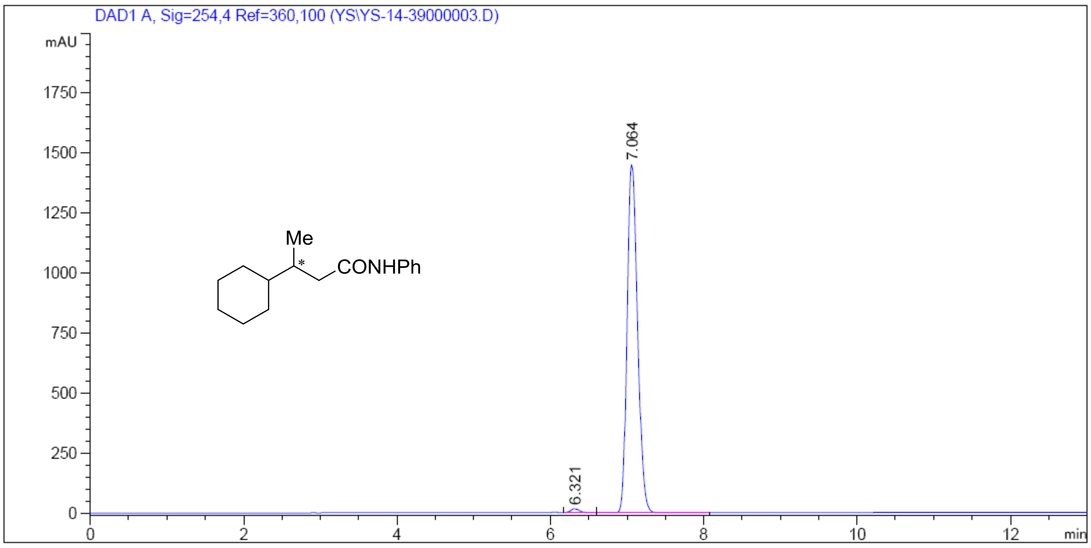

Peak #	RetTime [min]	Type	Width [min]	Area [mAU*s]	Height [mAU]	Area %
1	6.321	VB	0.1302	141.32686	16.79159	1.0200
2	7.064	BV	0.1504	1.37144e4	1449.69421	98.9800

(*R*)-3-Methyl-N,5-diphenylpentanamide (6h)

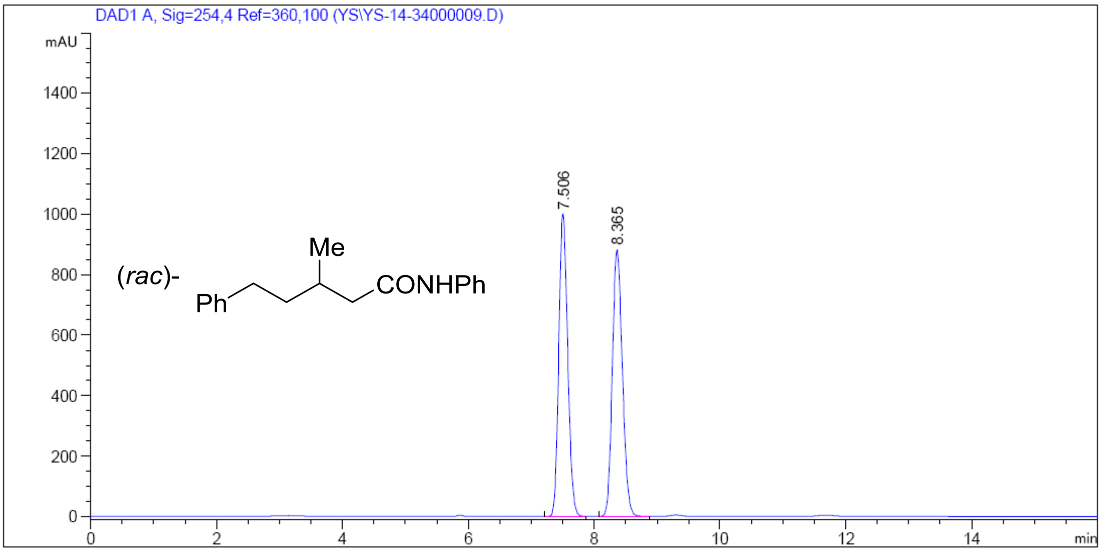

Peak #	RetTime [min]	Type	Width [min]	Area [mAU*s]	Height [mAU]	Area %
1	7.506	BV	0.1529	9857.53516	1000.85193	50.0341
2	8.365	VV	0.1742	9844.09570	882.00555	49.9659

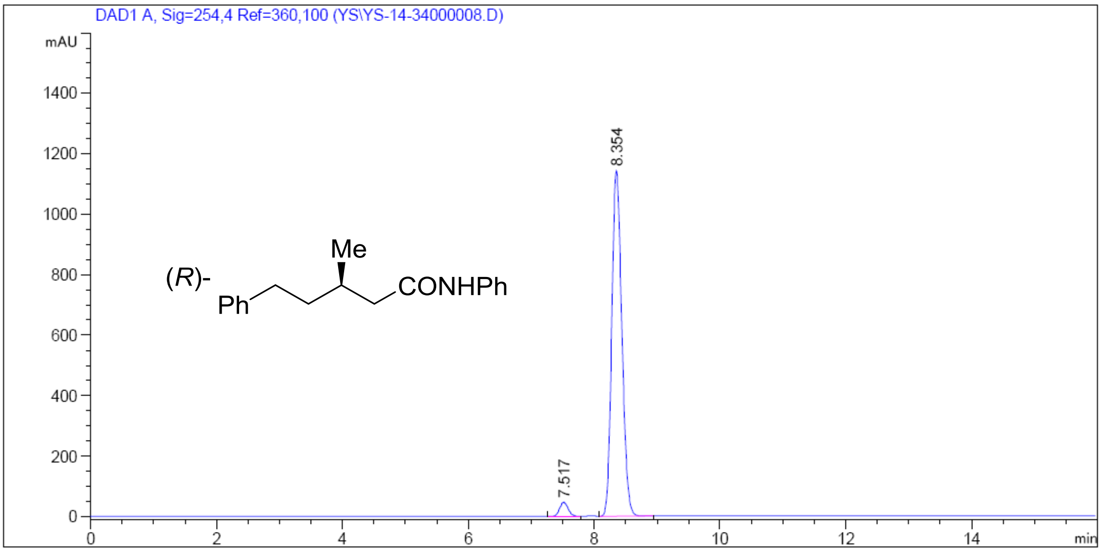

Peak #	RetTime [min]	Type	Width [min]	Area [mAU*s]	Height [mAU]	Area %
1	7.517	BB	0.1480	448.75876	46.75795	3.4106
2	8.354	VB	0.1738	1.27090e4	1142.24573	96.5894

7-Methoxy-3-methyl-N-phenylheptanamide (6i)

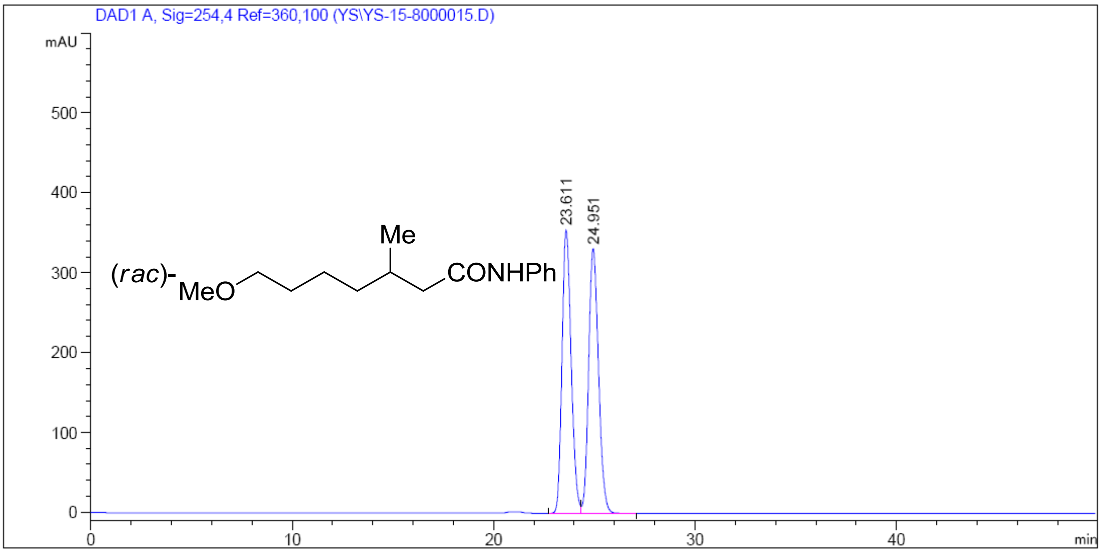

Peak #	RetTime [min]	Type	Width [min]	Area [mAU*s]	Height [mAU]	Area %
1	23.611	BV	0.4899	1.12302e4	354.21625	49.8598
2	24.951	VB	0.5299	1.12934e4	331.09943	50.1402

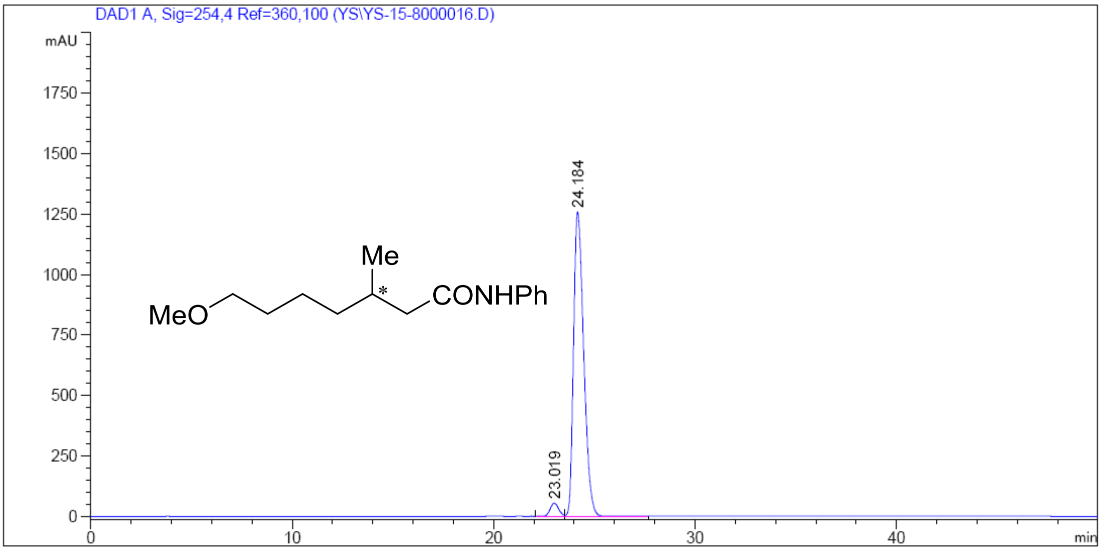

Peak #	RetTime [min]	Type	Width [min]	Area [mAU*s]	Height [mAU]	Area %
1	23.019	BV	0.4654	1639.00330	54.72998	3.5834
2	24.184	VB	0.5454	4.40996e4	1256.79285	96.4166

(*R*)-3,7-Dimethyl-N-phenyloct-6-enamide (6j)

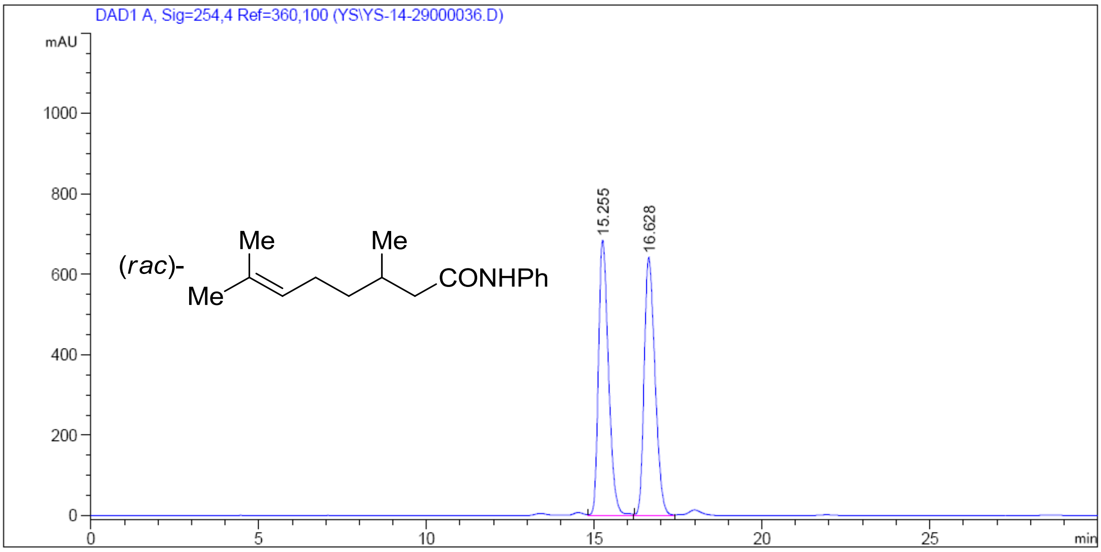

Peak #	RetTime [min]	Type	Width [min]	Area [mAU*s]	Height [mAU]	Area %
1	15.255	VV	0.3234	1.42686e4	684.06390	49.6822
2	16.628	VV	0.3549	1.44511e4	641.37976	50.3178

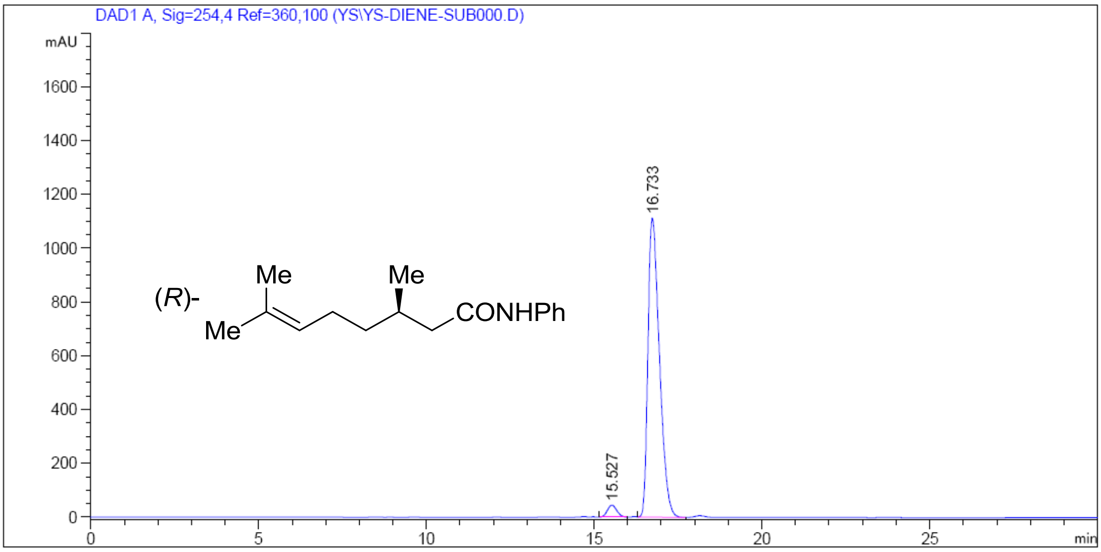

Peak #	RetTime [min]	Type	Width [min]	Area [mAU*s]	Height [mAU]	Area %
1	15.527	BV	0.3027	856.66803	44.07794	3.1317
2	16.733	VB	0.3677	2.64981e4	1113.18188	96.8683

(*S*)-2-Methyl-N,3-diphenylpropanamide (8a)

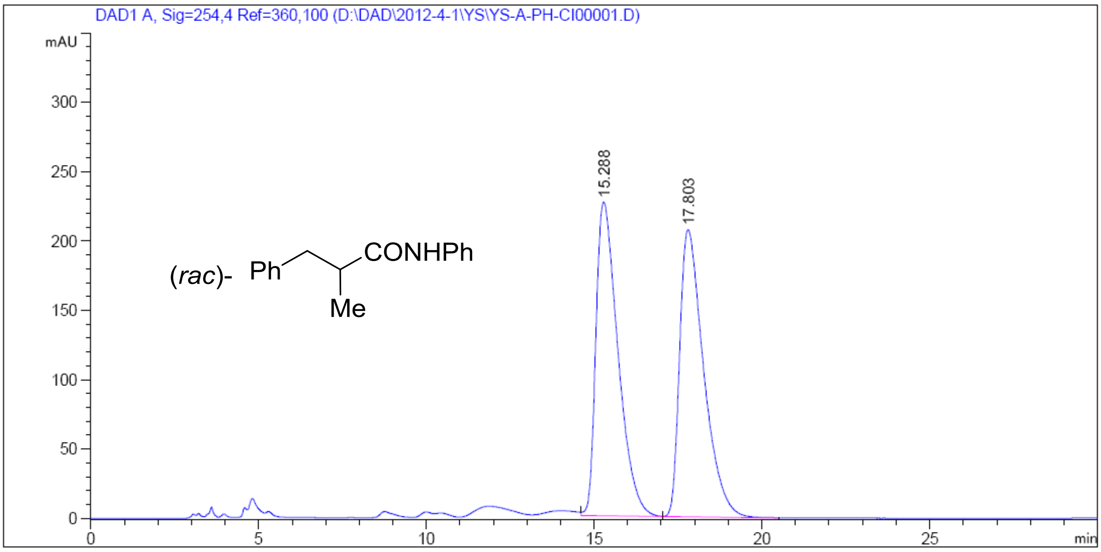

Peak #	RetTime [min]	Type	Width [min]	Area [mAU*s]	Height [mAU]	Area %
1	15.288	VB	0.7047	1.03246e4	226.10629	50.1158
2	17.803	BB	0.7557	1.02769e4	206.81168	49.8842

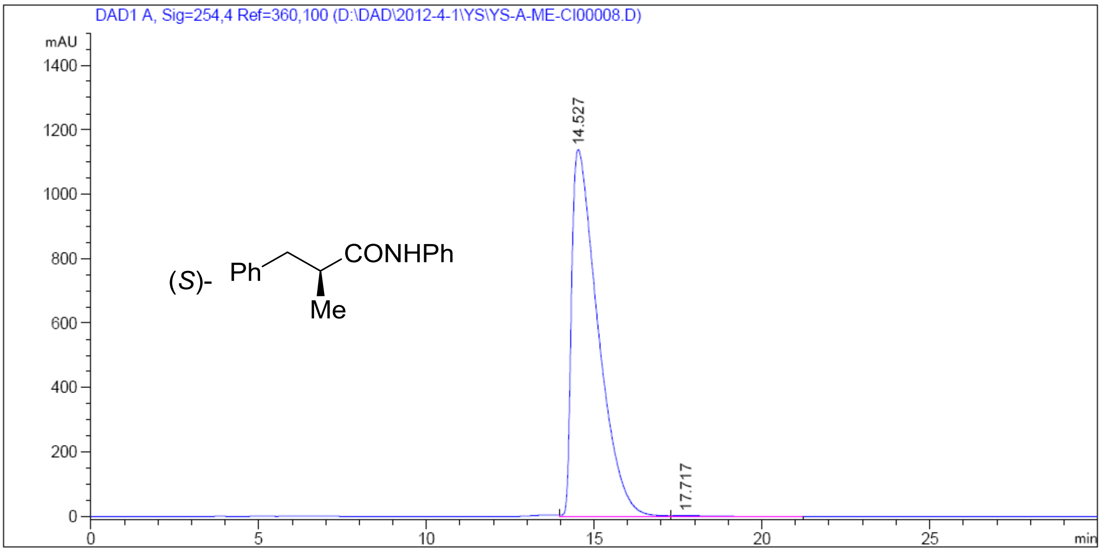

Peak #	RetTime [min]	Type	Width [min]	Area [mAU*s]	Height [mAU]	Area %
1	14.527	VV	0.8184	6.18576e4	1137.55176	99.7045
2	17.717	VB	0.8537	183.30193	2.97963	0.2955

(*S*)-2-Methyl-N-phenylbutanamide (8b)

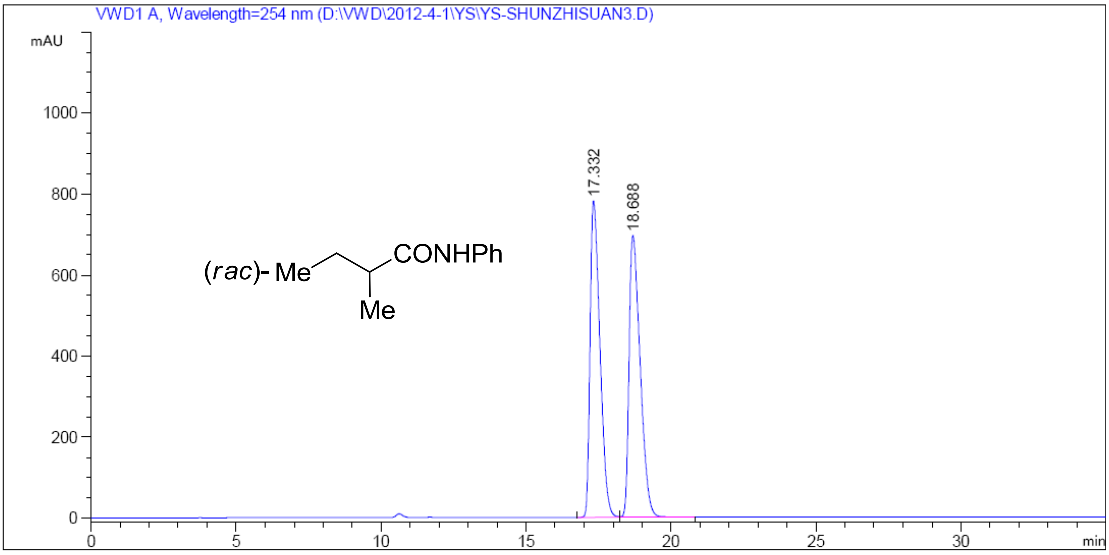

Peak #	RetTime [min]	Type	Width [min]	Area mAU *s	Height [mAU]	Area %
1	17.332	BV	0.3693	1.84984e4	781.70728	49.9414
2	18.688	VB	0.4086	1.85418e4	695.40247	50.0586

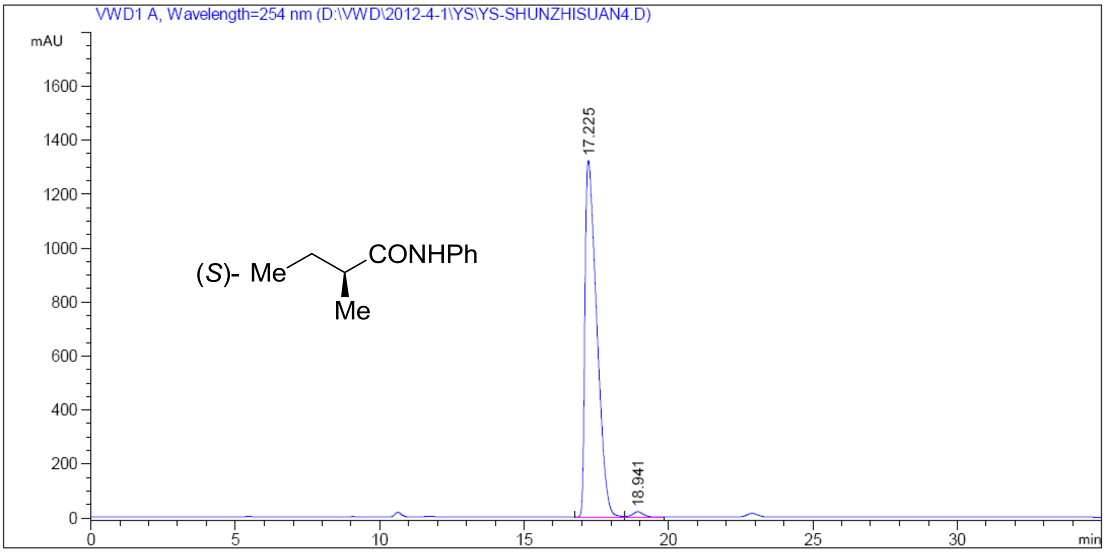

Peak #	RetTime [min]	Type	Width [min]	Area mAU *s	Height [mAU]	Area %
1	17.225	BV	0.4318	3.73694e4	1321.42676	98.6711
2	18.941	VB	0.3908	503.29144	19.63482	1.3289

(*R*)-N,2-Diphenylbutanamide (8c)

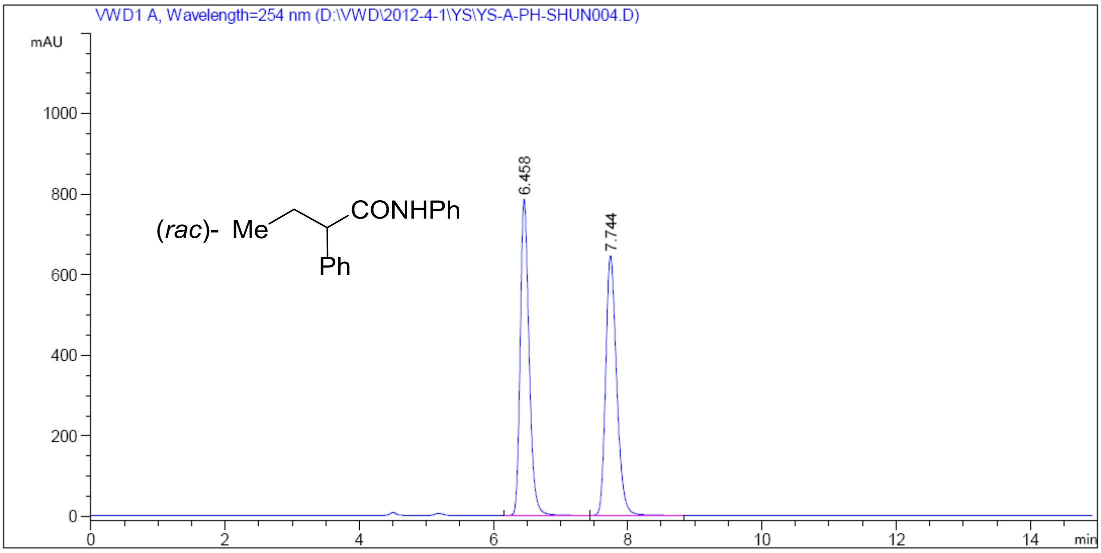

Peak #	RetTime [min]	Type	Width [min]	Area mAU *s	Height [mAU]	Area %
1	6.458	BB	0.1401	7166.04541	783.84027	49.3525
2	7.744	BB	0.1755	7354.07031	643.90826	50.6475

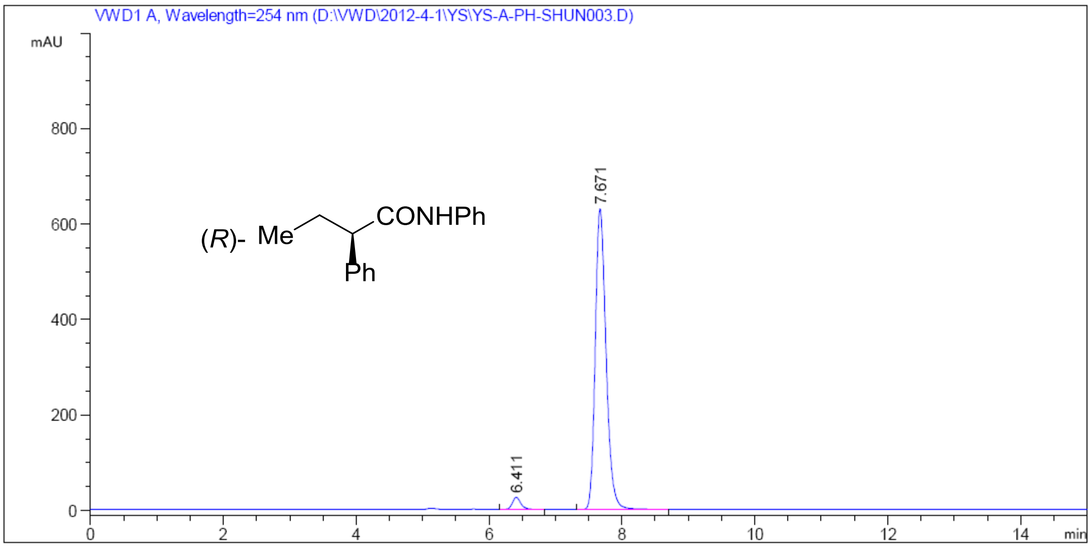

Peak #	RetTime [min]	Type	Width [min]	Area mAU *s	Height [mAU]	Area %
1	6.411	BB	0.1370	224.37556	25.29106	3.0565
2	7.671	BB	0.1741	7116.67090	629.62622	96.9435

(*S*)-2-(Benzyloxy)-N,3-diphenylpropanamide (8d)

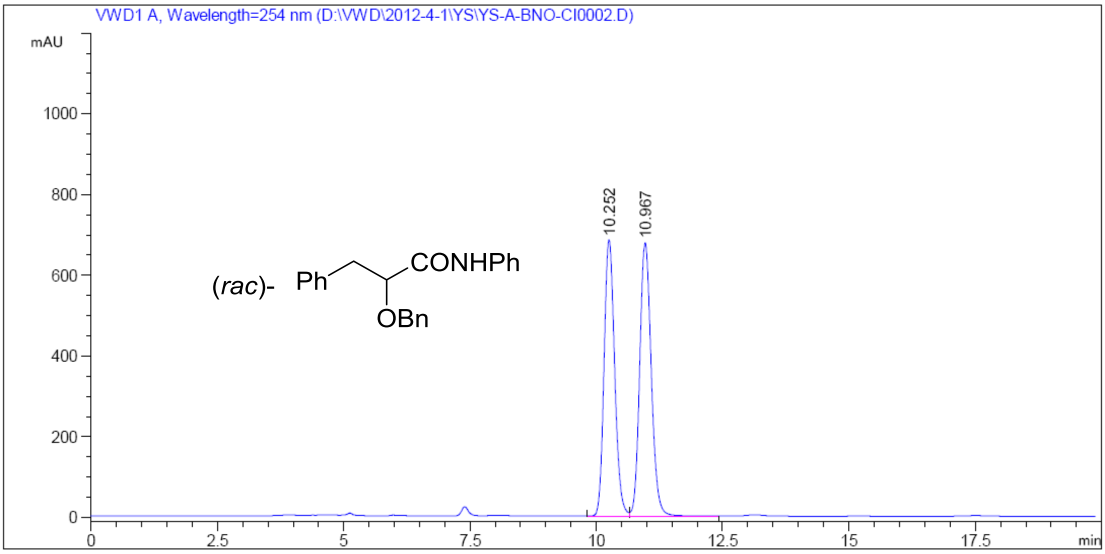

Peak #	RetTime [min]	Type	Width [min]	Area mAU *s	Height [mAU]	Area %
1	10.252	BV	0.2230	9829.23926	684.11475	48.0737
2	10.967	VB	0.2411	1.06169e4	677.37030	51.9263

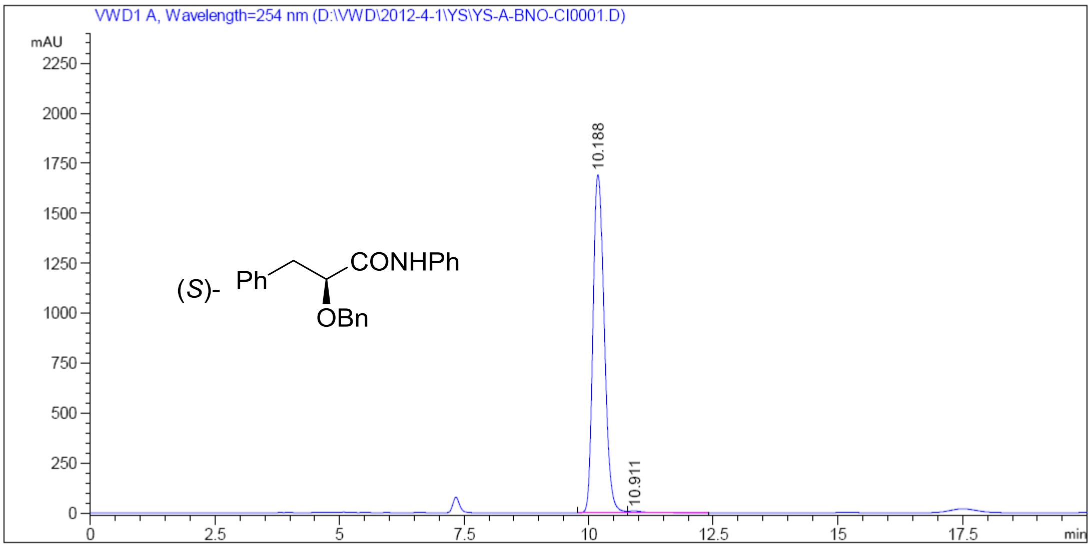

Peak #	RetTime [min]	Type	Width [min]	Area mAU *s	Height [mAU]	Area %
1	10.188	BV	0.2447	2.63625e4	1689.02222	99.2334
2	10.911	VB	0.3263	203.64937	8.72069	0.7666

(*R*)-Methyl 2-(4-isobutylphenyl)propanoate (8e)

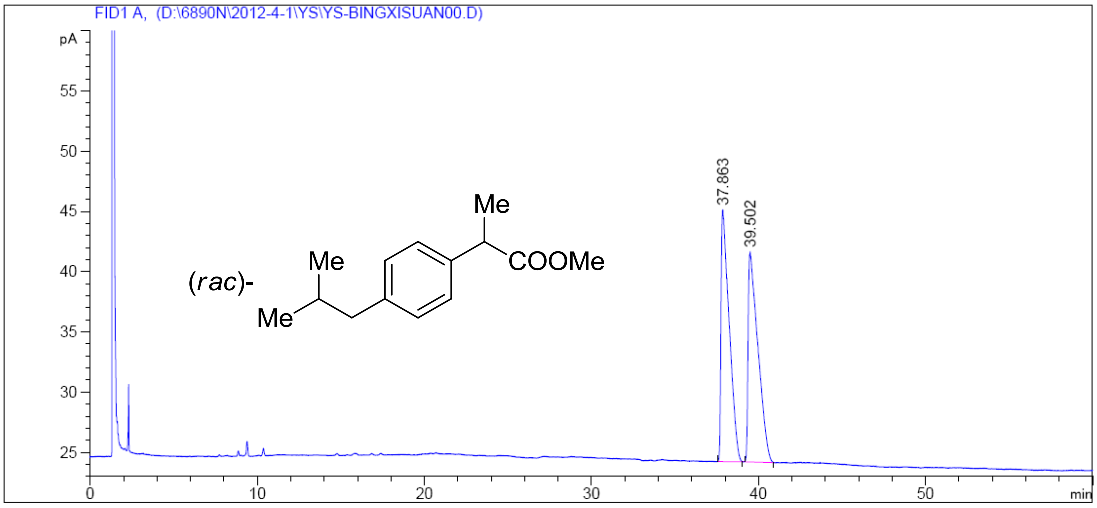

Peak #	RetTime [min]	Type	Width [min]	Area [pA*s]	Height [pA]	Area %
1	37.863	BB	0.4079	711.81049	20.87090	50.07094
2	39.502	BB	0.4835	709.79346	17.41664	49.92906

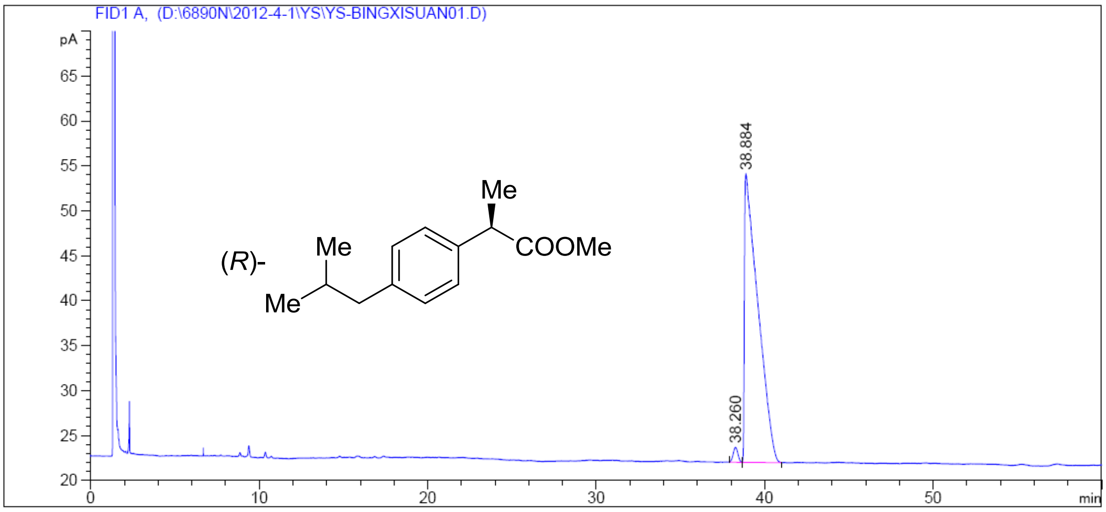

Peak #	RetTime [min]	Type	Width [min]	Area [pA*s]	Height [pA]	Area %
1	38.260	BV	0.2450	32.48083	1.65475	1.79662
2	38.884	VB	0.6741	1775.40771	32.05692	98.20338

(*R*)-2-Methyl-N-phenylheptanamide (8f)

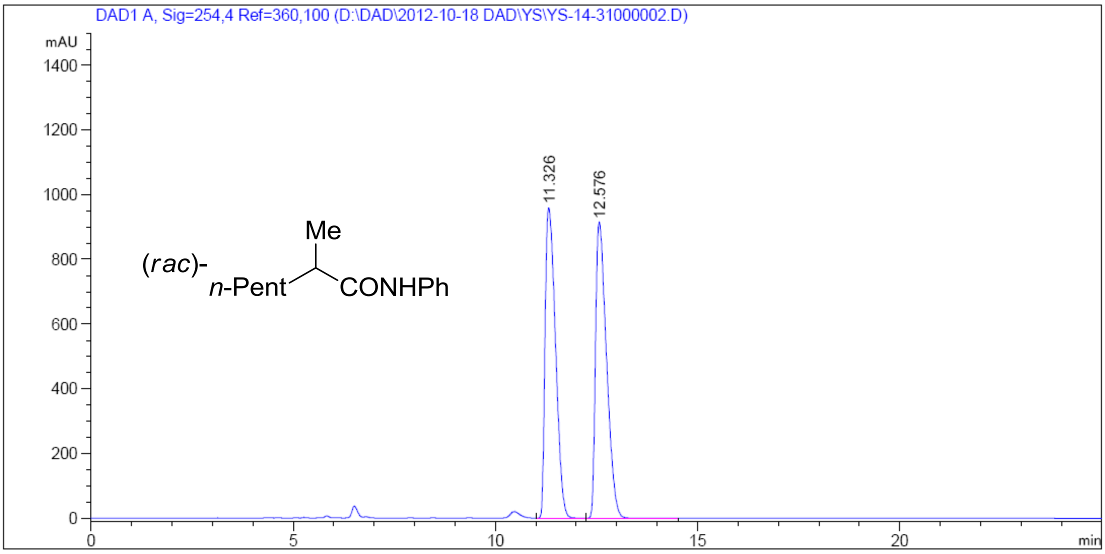

Peak #	RetTime [min]	Type	Width [min]	Area [mAU*s]	Height [mAU]	Area %
1	11.326	BB	0.2891	1.71939e4	959.26465	49.9266
2	12.576	BB	0.2921	1.72444e4	914.18628	50.0734

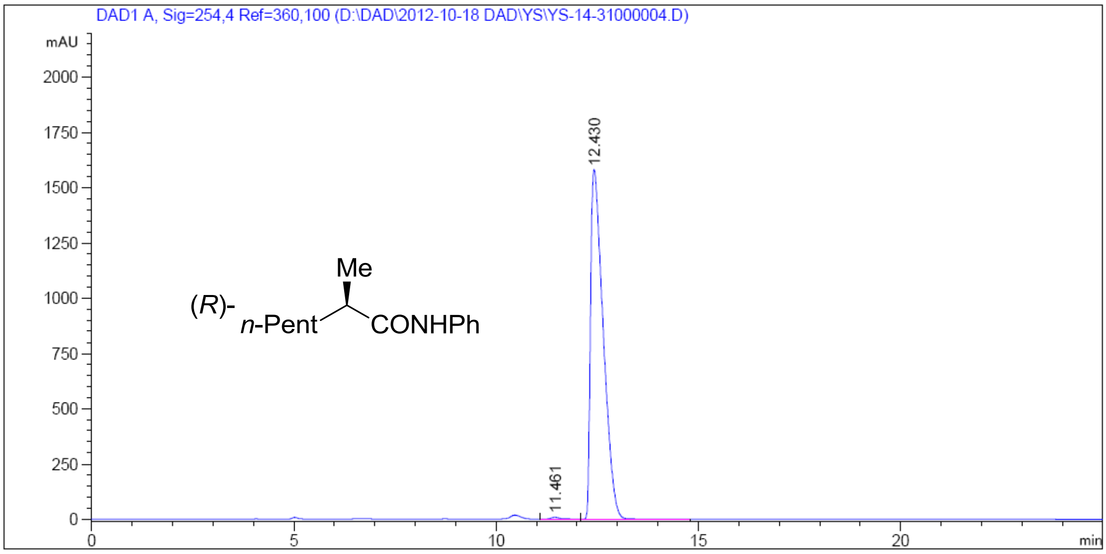

Peak #	RetTime [min]	Type	Width [min]	Area [mAU*s]	Height [mAU]	Area %
1	11.461	BB	0.2423	127.62109	7.99100	0.3694
2	12.430	BB	0.3301	3.44237e4	1580.23743	99.6306

8. Reference:

1 J. L. Herde, J. C. Lambert and C. V. Senoff, *Inorg. Synth.*, 1974, **15**, 18.

2 X. Sun, L. Zhou, C.-J. Wang and X. Zhang, *Angew. Chem. Int. Ed.*, 2007, **46**, 2623.

3 S. G. Alcock, J. E. Baldwin, R. Bohlmann, L. M. Harwood and J. I. Seeman, *J. Org. Chem.*, 1985, **50**, 3526.

4 Y. Zhang, Z.-B. Han, F.-Y. Li, K.-L. Ding and A. Zhang, *Chem. Commun.*, 2010, **46**, 156.

5 S. Li, S.-F. Zhu, J.-H. Xie, S. Song, C.-M. Zhang and Q.-L. Zhou, *J. Am. Chem. Soc.*, 2010, **132**, 1172.

6 R. R. Kurtz and D. J. Houser, *J. Org. Chem.*, 1981, **46**, 202.

7 D. V. Kummer, W. J. Chain, M. R. Morales, O. Quiroga and A. G. Myers, *J. Am. Chem. Soc.*, 2008, **130**, 13231.

8 B.-F. Li, R. M. Hughes, J. Le, K. McGee, D. J. Gallagher, R. S. Gross, D. Provencal, J. P. Reddy, P. Wang, L. Zegelman, Y. Zhao and S. E. Zook, *Org. Process Res. Dev.*, 2009, **13**, 463.

9 E. Reyes, J. L. Vicario, L. Carrillo, D. Badia, U. Uria and A. Iza, *J. Org. Chem.*, 2006, **71**, 7763.

10 K. C. Rice, *J. Org. Chem.*, 1982, **47**, 3617.

11 J. M. Garcia, A. Gonzalez, B. G. Kardak, J. M. Odriozola, M. Oiaarbide, J. Razkin and C. Palomo, *Chem. Eur. J.*, 2008, **14**, 8768.

12 S. Sugiyama and T. Satoh, *Tetrahedron: Asymmetry*, 2005, **16**, 665.

13 P. Heretsch, S. Rabe and A. Giannis, *Org. Lett.*, 2009, **11**, 5410.

14 S. G. Davies, D. J. Dixon, G. J.-M. Doisneau, J. C. Prodger and H. J. Sanganee, *Tetrahedron: Asymmetry*, 2002, **13**, 647.

15 S. Li, S.-F. Zhu, C.-M. Zhang, S. Song and Q.-L. Zhou, *J. Am. Chem. Soc.*, 2010, **132**, 1172.

16 I. Shiina, K. Nakata, K.Ono, Y. Onda and M. Itagaki, *J. Am. Chem. Soc.*, 2010, **132**, 11629.

17 V. Gopalsamuthiram, R. Huang and W. D. Wulff, *Chem. Commun.*, 2010, **46**, 8213.

18 T. Fujiwara, M. Sasaki, K. Omata, C. Kabuto, K. Kabuto and Y. Takeuchi, *Tetrahedron: Asymmetry*, 2004, **15**, 555.

19 N. Harrington-Frost, H. Leuser, M. I. Calaza, F. F. Kneisel and P. Knochel, *Org. Lett.*, 2003, **5**, 2111.

20 R. Y. Kharisov, E. R. Latypova, R. F. Talipov, R. R. Muslukhov, G. Y. Ishmuratov and G. A. Tolstikov, *Russ. Chem. Bull. Int. Ed.*, 2003, **52**, 2267.
